# Food insecurity in New York City college students during the COVID-19 pandemic: a longitudinal analysis of predictors, correlates, and trajectories

**DOI:** 10.1080/07448481.2026.2674845

**Published:** 2026-05-31

**Authors:** Craig J. Heck, Deborah A. Theodore, April Autry, Brit Sovic, Cynthia Yang, Sarah Ann Anderson-Burnett, Caroline Ray, Eloise Austin, Joshua Rotbert, Jason Zucker, Marina Catallozzi, Magdalena E. Sobieszczyk, Delivette Castor

**Affiliations:** a Division of Infectious Diseases, Department of Medicine, Columbia University Irving Medical Center, New York, New York, USA;; b Department of Epidemiology, Columbia University Mailman School of Public Health, New York, New York, USA;; c Barnard College, Health & Wellness, New York, New York, USA;; d Division of Child and Adolescent Health, Department of Pediatrics, Columbia University Irving Medical Center, New York, New York, USA;; e Heilbrunn Department of Population & Family Health, Columbia University Mailman School of Public Health, New York, New York, USA

**Keywords:** College health, disparities, pandemic preparedness, poverty, youth

## Abstract

**Objective:**

To examine predictors, correlates, and trajectories of food insecurity (FI) among students during COVID-19.

**Participants:**

Undergraduates

**Methods:**

Between 2020–2021, students completed quarterly (T1–T4) self-administered questionnaires. FI comprised food not lasting or not affording balanced meals. Trajectories were created using cumulative FI reports.

**Results:**

Across time, FI varied (T1 = 21%, T2 = 25%, T3 = 17%, T4 = 19%). FI predictors included preexisting FI, need-based aid, low social support, alcohol use, COVID-19 care-seeking behaviors, violence experience, transgender/gender non-conforming [TGNC] identity, and unsafe home perceptions. FI trajectories spanned Never (69%), Rarely (12% FI once), Frequently (14% FI twice/thrice), and Persistently (5%). Along with low social support, Persistently FI students had high psychological distress, need-based aid, unsafe home perceptions, smoking/vaping, drug use, TGNC representation, violence experience, and COVID-19 care-seeking behaviors.

**Conclusions:**

FI was associated with sociodemographic, residential, interpersonal, psychosocial, behavioral, and healthcare-related factors. In routine campus operations and emergency situations, universities must develop multi-component interventions to address multi-factorial stressors.

## Introduction

Food insecurity (FI) is an economic and structural vulnerability that undermines physical, psychosocial, and developmental health in the United States (US).^1^ The United States Department of Agriculture (USDA) defines FI as “limited or uncertain availability of nutritionally adequate and safe foods, or limited or uncertain ability to acquire acceptable foods in socially acceptable ways.”^2^ Concerningly, FI disproportionately affects college students.^3^ Two reviews examining evidence between 1995 and 2021 underscore that an estimated 33–44% of US college students experience FI, compared with 13% of households in the general population.^4–6^ A recent review on research trends and gaps highlights that FI among US college students is highly variable and affected by a myriad of social, structural, and behavioral factors.^7^ To better develop, tailor, and target interventions, it is imperative to understand factors influencing college students’ FI.

FI is a complex phenomenon underpinned by various factors. Demographically, FI is higher in historically marginalized racial, ethnic, and gender identities and among those receiving need-based financial aid, like Pell grants.^7–10^ Residential dynamics, including cohabitant relations, location, and safety, affect FI by influencing food availability and economic support.^11^ Interpersonally, social and sports groups can facilitate FI by introducing additional time and financial commitments. Relationships, romantic or casual, can also link to FI *via* violence, power differentials, and survival-related behaviors.^12,13^ FI can also arise if individuals use limited financial resources for competing priorities, such as cigarettes/vapes, alcohol, and drugs.^14–16^ Additionally, FI may complicate use or access of health necessities, like contraceptive care or gender-affirming treatment.^17,18^ Loneliness, low social support, and stress/distress are consistently related to FI.^19^

The COVID-19 pandemic magnified FI in college students by introducing new stressors while exacerbating existing ones. Compared with pre-pandemic conditions, food security respectively worsened in 15% and 20% of students enrolled at colleges in the Midwest and Southeast US.^20,21^ Disruptions to typical housing and learning structures may have contributed to these outcomes. COVID-induced shifts in learning formats, residential environments and compositions, and geographic location have been associated with FI in this population.^22,23^ Our prior research supports that during remote learning, FI and psychological distress were related in cisgender undergraduate women. In a follow-up longitudinal analysis, FI emerged as an important predictor of distress and a key characteristic of those who were persistently distressed.^24,25^ FI could have also led students to forego seeking out medical care for COVID-related symptoms and/or complications.^26^ Given the pandemic’s unique circumstances, conditions, and antagonists, explicating FI during this period could provide vital insights for designing and delivering preventative and interventional countermeasures during future emergencies and beyond.

FI among US college students during COVID-19 remains poorly understood due to a paucity of temporal insights. Current longitudinal FI studies primarily examine either nationally representative or adult samples, and most analyses with US college students assess FI cross-sectionally or across two timepoints.^7,20,21,27–30^ With FI’s temporal dynamics and associations largely unknown, this investigation aimed to elucidate FI’s longitudinal relationships with sociodemographic, residential, interpersonal, behavioral, psychosocial, and healthcare-related factors. To achieve this goal, we performed analyses across four quarterly timepoints (2020–2021) to illuminate predictors, correlates, and trajectories of FI within students enrolled at a New York City (NYC) college.

## Methods

### Cohort design

Our previous publications describe the cohort’s design in detail.^24,25,31^ Briefly, consenting students, faculty, and staff associated with a private college in NYC completed quarterly (T1: December 2020/January 2021, T2: March/April 2021, T3: July/August 2021, T4: November/December 2021) internet-based questionnaires on COVID-19 and its effect on their health and wellbeing. The college’s student population around the time of survey completion was composed of approximately 46% non-Hispanic white, 18% Asian, 12% Hispanic, 13% Other/Multiracial, and 12% international students. Affiliates aged ≥18 years who understood English and had an institutional email address were eligible to participate. Self-reported data were collected *via* Research Electronic Data Capture (REDCap).^32^

### Sample

The analytic sample was restricted to students to focus on young adulthood. We operationalized lost to follow-up (LTFU) as 1) missing a survey or 2) providing no FI data.

### Methodological framing

A previous analysis of mental health trajectories within this cohort showed that FI was associated with greater levels of psychological distress, and FI was higher among students with distress at all timepoints.^25^ Guided by these observations and the cohort’s unique design and duration, we used a similar analytic framework to investigate factors associated with FI.

### Measures

Detailed definitions of all measures are in Supplemental Material (SM) 1.

### Primary/dependent variable

The questionnaire asked two questions from USDA’s 10-item Adult Food Security Survey Module: in the last 30 days, inability to 1) buy more food when it ran out or 2) afford to eat balanced meals. Response options included Often, Sometimes, or Never True. In the general population, USDA classifies food security using the number of affirmative answers (Often/Sometimes True), encompassing highly secure (0 items), marginally (1–2 items) secure, and FI (>2 items).^2,33^

We defined FI as answering affirmatively to either question (i.e., 1–2 items) vs. Never True to both questions. We used this approach because prior research in NYC’s metro area found marginally secure and FI college students were more alike than highly and marginally secure students, facilitating recommendations to reclassify marginally secure as FI in this unique population.^34^

### Independent variables

For sociodemographic factors, we assessed age, racial and ethnic identity, school year upon enrollment, gender identity (cisgender woman vs. transgender or gender-nonconforming [TGNC]), receipt of need-based financial aid, and when reporting predictors, existing FI. Residential elements included cohabitants, location, and perceived safety in home. Interpersonal variables comprised social group and sports involvement, relationship status, experience of physical/verbal violence, and condom use. Behaviors spanned currently smoking/vaping and frequency of alcohol use and drug use. Loneliness, social support, and moderate-severe psychological distress (MS-PD) encompassed the psychosocial domain. Healthcare variables were use of hormones (for contraceptive or gender-affirming purposes) and seeking care for COVID-19 symptoms.

### Statistical analyses

We performed Chi-square and Kruskal-Wallis rank sum tests for tabular analyses. All data were managed, transformed, and analyzed using R (v.4.4.0).

#### Predictions and correlations

We estimated risk ratios using Poisson regressions with sandwich estimators to elucidate FI predictors.^35^ All simple regressions controlled for preexisting FI status because preexisting FI consistently predicted future FI; we refer to these models as minimally adjusted. To construct adjusted models, we first fit all factors with a minimally adjusted *p* ≤ 0.10 into a model. Then, we performed backward elimination by removing the variable with the largest p-value and re-running the model until all factors were statistically significant (α = 0.05). When violence experience was eligible for multivariable inclusion, we fitted it into a separate final model because its adjustment restricts the sample to partnered/cohabiting participants. We report predictor-outcome timing using T#–T# (e.g., T1–T2).

We used generalized estimating equations (GEE) models with an autoregressive matrix to estimate marginal effects among those with complete FI data. The adjusted GEE model was created using the same approach as the predictive models.

We also conducted correlative analyses because each timepoint reflected a unique pandemic period. T1 questionnaires were completed during COVID’s Wave 2, when remote learning and strict pandemic countermeasures (e.g., travel restrictions, social distancing) were in full effect. T2 corresponded to the tail-end of Wave 2, the initial vaccine rollout, and the beginning of countermeasure abatement. T3 overlapped with Wave 3, expanding vaccine eligibility, and continued relaxation of countermeasures. At T4, Wave 4 was occurring as students were back on campus for in-person learning, with minimal countermeasures in place. We use correlations to support the temporal analyses while highlighting unique period-dependent associations.

#### Trajectory analyses

In participants with complete outcome data, we used cumulative FI events to create trajectories; we characterized these groupings by examining between- and within-trajectory differences.

#### LTFU analyses

We assessed LTFU by examining T1 characteristic differences in participants 1) lost vs. included over time using GEE models (autoregressive matrix; LTFU-GEE), and 2) excluded vs. included in the trajectory sample (LTFU-Traj) using Poisson regressions (sandwich estimators).

## Results

### Sample

Of all eligible screened participants (*N* = 977), 728 (75%) consented and 668 (68%) completed the T1 survey. At T1, 556 students were included in the sample after excluding faculty (*n* = 36), staff (*n* = 69), and non-respondents to FI questions (*n* = 6). Across time, 334, 222, and 167 students respectively comprised the T2, T3, and T4 samples (SM2).

#### Sample characteristics

At T1 (*N* = 556), the sample had a median age of 20 years; most were White-identifying (63%), non-Hispanic (86%), cisgender women (97%), who did not receive need-based financial aid (56%; Table 1). Less common characteristics included unsafe home perceptions (24%), partnerships/relationships (30%), current smoking/vaping (8%), frequent use of alcohol (31%) and drugs (21%, with ≥83% only using cannabis over time), low social support (uncertain: 13%, none: 13%), and current hormone use (35%).

Over time (T1 to T4), overall changes in living situation and geographic location coincided with the academic calendar and pandemic countermeasure abatement; most students lived on/near campus at T2 (hybrid learning, waning distancing protocols) and T4 (full in-person learning for Fall semester) and at home at T3 due to summer break. Conversely, at every timepoint, most (>60%) TGNC-identifying students lived alone or with friends on/near campus (data not shown). Social group involvement fluctuated (T1: 61%, T2: 49%, T3: 39%, T4: 60%), whereas sports group engagement (13% to 8%) and violence experience (51% to 15%) decreased. Increases in condom use (19% to 30%) overlapped with decreasing loneliness (41% to 17%) and MS-PD (74% to 55%). COVID-19 health-seeking behaviors were also variable.

FI was experienced by few students and did not appreciably change over time (21% to 19%). Among those reporting FI, most reported either both FI indicators (T1 = 48%, T2 = 41%, T4 = 50%) or inability to afford balanced meals (T3 = 51%; SM3). FI among cisgender women was similar to the overall sample over time (T1: 20%, T2: 23%, T3: 16%, T4: 17%), whereas FI among TGNC-identifying students was considerably higher over time (T1: 47%, T2: 53%, T3: 25%, T4: 55%).

### FI associations

#### Predictors

**Figure 1.**
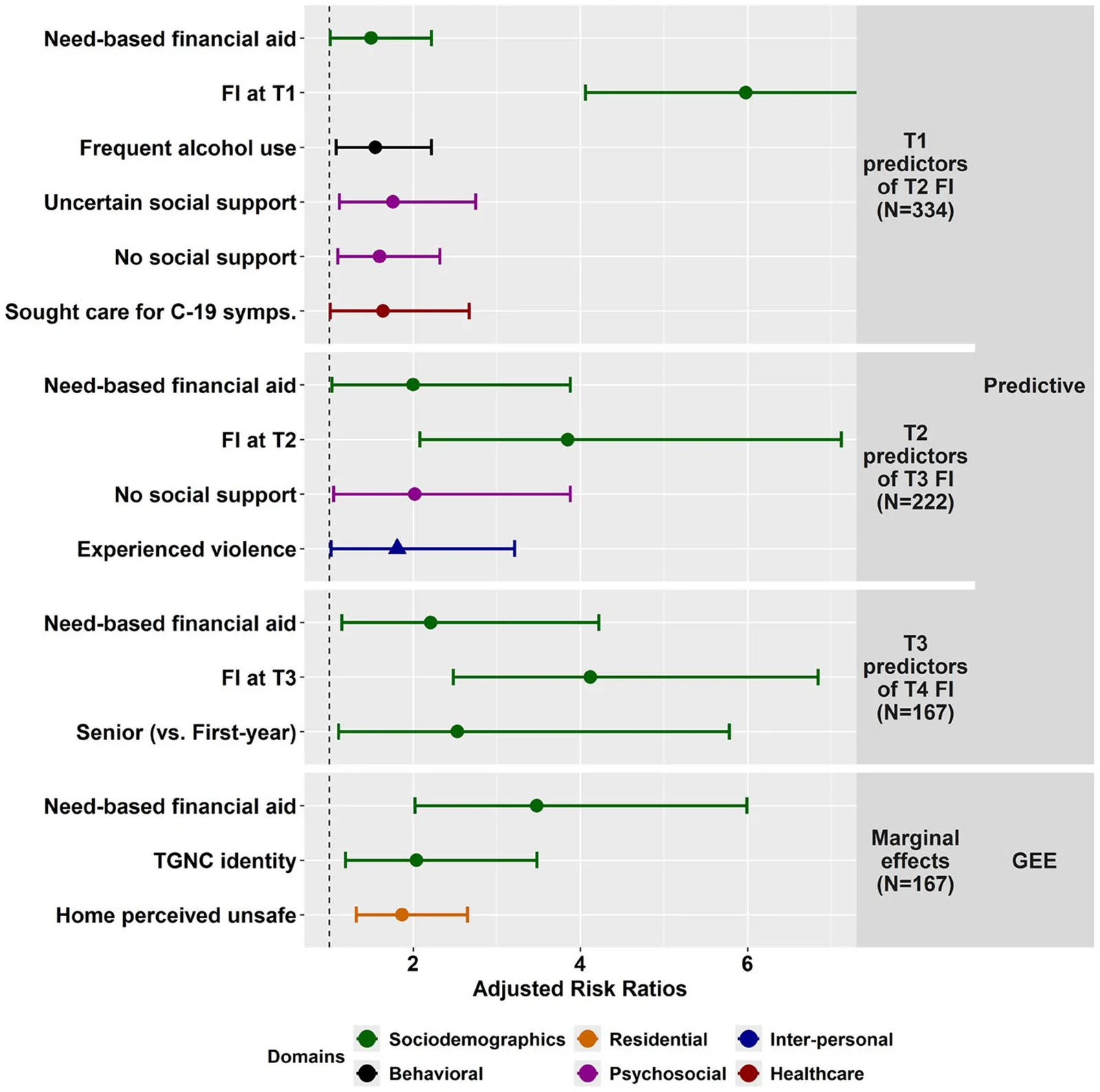
Predictors of FI. C19: COVID-19; TGNC: Transgender/Gender non-conforming; FI: Food Insecurity; GEE: Generalized Estimating Equations. Reference category for “Sought care for C-19 symps.” is No symptoms. Circle symbols indicate the factors were fitted in the same model. Triangle symbols reflect fitting into a separate model because inclusion of violence restricts the sample to those who were partnered/cohabiting. Error bars reflect 95% confidence intervals. The upper 95% confidence interval limit for FI at T1 is 8.80. Complete outputs are presented in SMs 4–7.

Figure 1 showcases the predictors of FI across time (SM4–6). At every timepoint, receipt of need-based financial aid (T1–T2: Adjusted Risk Ratio [ARR] = 1.50 [1.01–2.22], T2–T3: ARR = 2.00 [1.03–3.88], T3–T4: ARR = 2.21 [1.15–4.22]) and preexisting FI (T1–T2: ARR = 5.98 [4.06–8.80], T2–T3: ARR = 3.85 [2.08–7.12], T3–T4: ARR = 4.12 [2.48–6.84]) predicted future FI. Low social support also arose as an important predictor in the T1–T2 (uncertain: ARR = 1.76 [1.12–2.75], none: ARR = 1.60 [1.10–2.32]) and T2–T3 (none: ARR = 2.02 [1.05–3.88]) analyses.

The remaining unique predictors included frequent alcohol use (T1–T2: ARR = 1.55 [1.08–2.22]), seeking care for COVID-19 symptoms (T1–T2: ARR = 1.64 [1.01–2.67], ref: no symptoms), experiencing violence (T2–T3: ARR = 1.81 [1.02–3.21]), and being a Senior (T3–T4: ARR = 2.53 [1.11–5.78], ref: First-year).

#### Marginal effects

Figure 1 also presents findings from the GEE models (SM7). On average, risk of FI was higher in students who received need-based financial aid (ARR = 3.48 [2.02–5.99]), identified as TGNC (ARR = 2.04 [1.19–3.48]), and perceived their home as unsafe (ARR = 1.87 [1.32–2.65]).

#### Correlates

**Figure 2.**
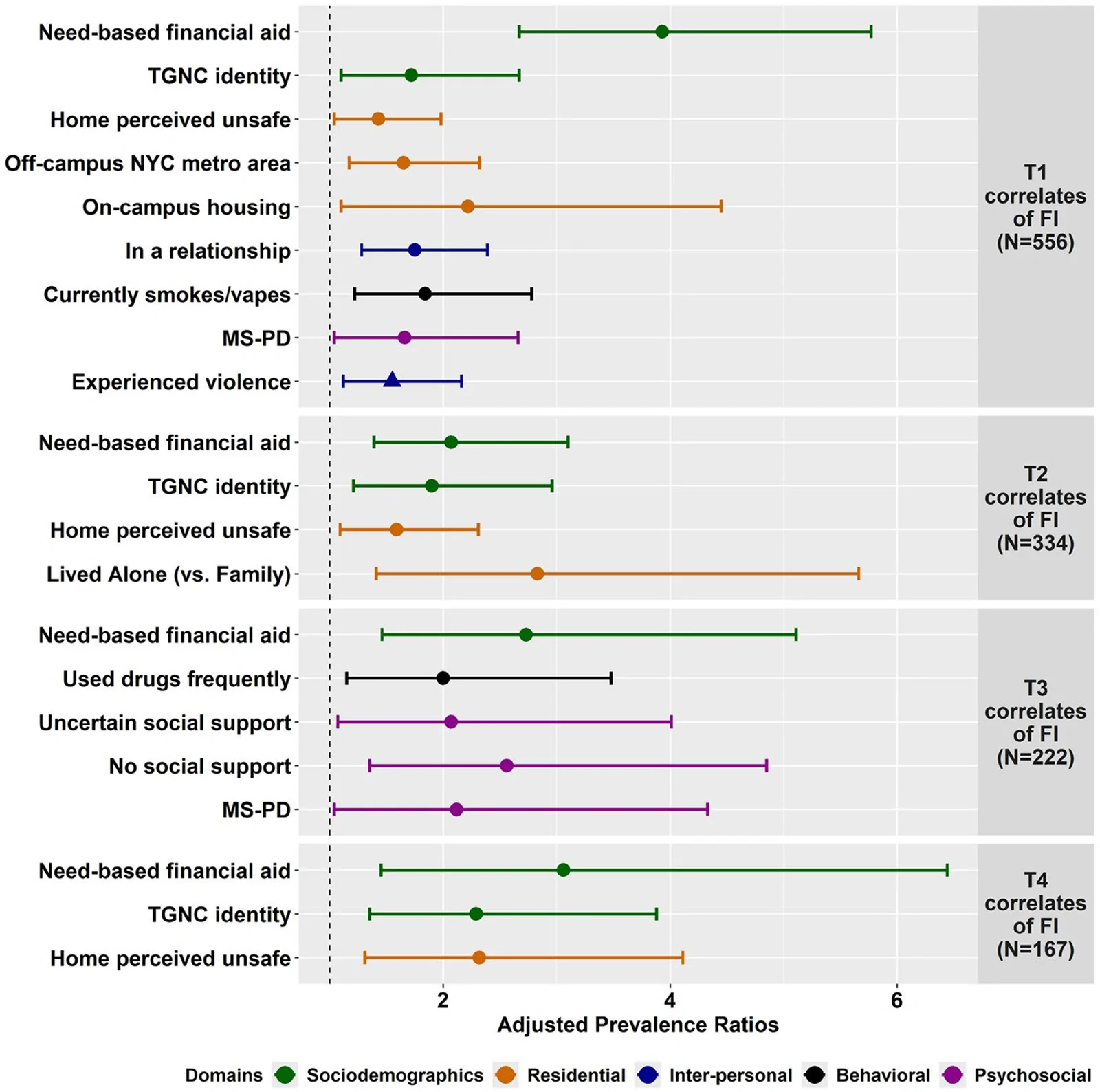
Correlates of FI. TGNC: Transgender/Gender non-conforming; NYC: New York City; MS-PD: Moderate-Severe Psychological Distress; FI: Food Insecurity. Circle symbols indicate the factors were fitted in the same model. Triangle symbols reflect fitting into a separate model because inclusion of violence restricts the sample to those who were partnered/cohabiting. Error bars reflect 95% confidence intervals. Complete outputs are presented in SMs 8–11.

Figure 2 highlights the quarter-specific correlates of FI (SM8–11). Corroborating the predictive and GEE models, need-based financial aid (T1: Adjusted Prevalence Ratio [APR] = 3.93 [2.67–5.77], T2: APR = 2.07 [1.39–3.10], T3: APR = 2.73 [1.46–5.11], T4: APR = 3.06 [1.45–6.44]), TGNC identity (T1: APR= 1.72 [1.10–2.67], T2: APR = 1.90 [1.21–2.96], T4: APR = 2.29 [1.35–3.88]), and unsafe home perceptions (T1: APR = 1.43 [1.04–1.98], T2: APR = 1.59 [1.09–2.31], T4: APR = 2.32 [1.31–4.11]) were consistently correlated with FI across timepoints. Moderate-severe psychological distress also arose as a correlate at T1 (APR = 1.66 [1.04–2.66]) and T3 (APR = 2.12 [1.04–4.33]).

Correlates specific to T1 included geographic location (lived off-campus: APR = 1.65 [1.17–2.32], lived on-campus; APR = 2.22 [1.10–4.45], ref: Outside NYC metro), being in a relationship (APR = 1.75 [1.28–2.39]), currently smoking/vaping (APR = 1.84 [1.22–2.78]), and violence experience (APR= 1.55 [1.12–2.16]). The only unique T2 correlate was living alone (APR = 2.83 [1.41–5.66], ref: Lived with family). At T3, frequent drug use (APR = 2.00 [1.15–3.48]) and low social support (uncertain: APR = 2.07 [1.07–4.01], none: APR = 2.56 [1.35–4.85]) were positively associated with FI.

### Composition and characteristics of FI trajectories

#### Sample characteristics

In total, the trajectory sub-sample included 167 respondents, and their characteristics did not appreciably differ from the overall sample (SM12).

#### Trajectory creation

Table 2 highlights the composition and creation of the FI trajectories. Most participants (69%) never reported FI, whereas 13%, 8%, 5%, and 5% respectively reported it once, twice, thrice, and persistently. We categorized trajectories as Never, Rarely (FI once), Frequently (FI twice/thrice), and Persistently FI. For the Rarely trajectory (*N* = 21), FI proportions were highest at T4 (43%), T3 (24%), and T2 (24%). The Frequently trajectory (*N* = 23) included students who primarily experienced FI at T3 and T4 (22%), T2 and T4 (17%), T1 and T2 (17%), T1 through T3 (17%), and all but T3 (13%).

#### Between-trajectory differentiations

**Figure 3.**
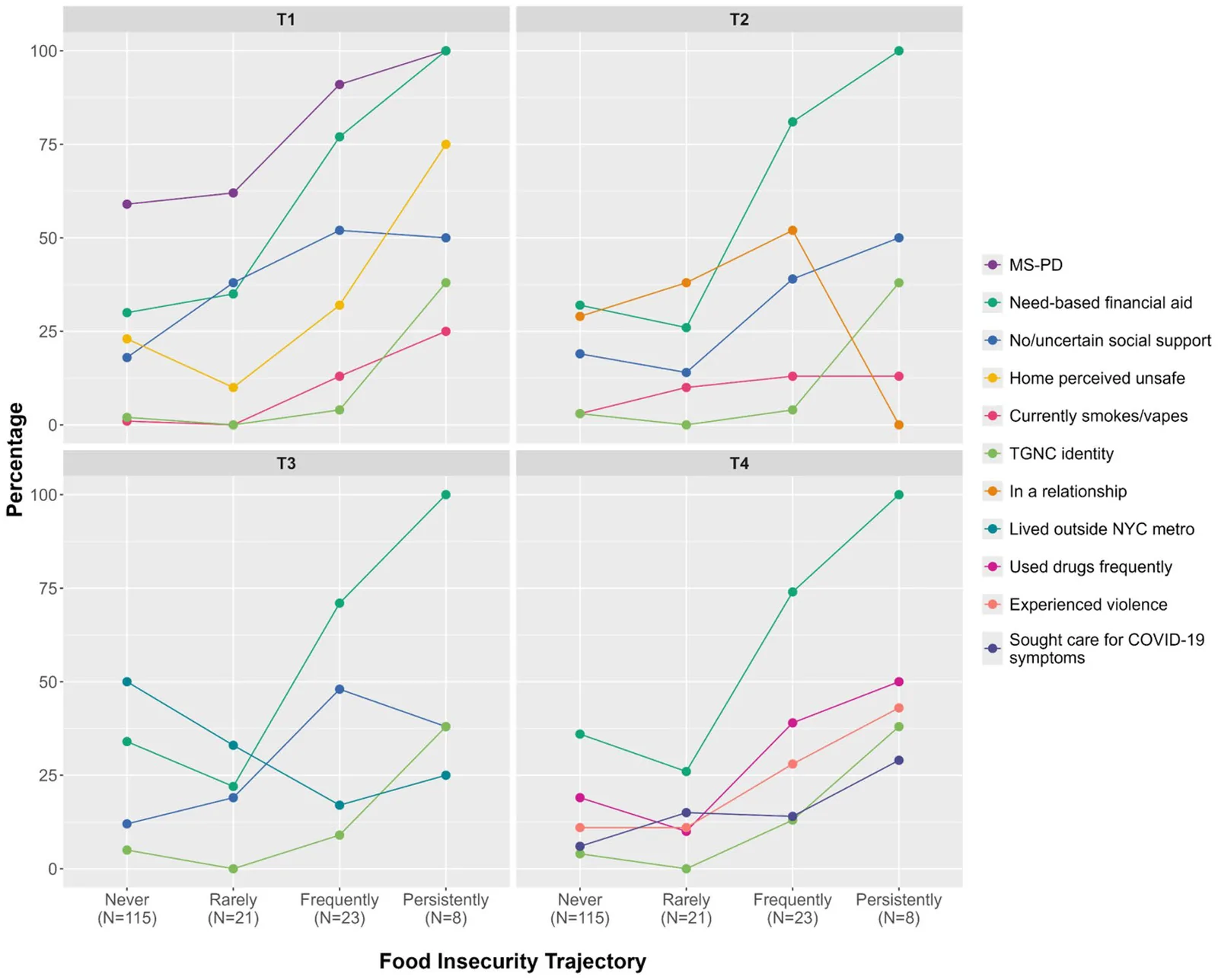
Inter-trajectory characteristic differences. MS-PD: Moderate-Severe Psychological Distress; TGNC: Transgender/Gender non-conforming; NYC: New York City. Complete outputs are presented in SM 13.

Figure 3 presents the inter-trajectory differences (SM13). Compared with other trajectories (temporal attribution reported in parentheses), the Persistently FI trajectory had high/highest MS-PD (T1), receipt of need-based financial aid (T1–T4), perceptions of an unsafe home (T1), no/uncertain social support (T1–T3), TGNC representation (T1–T4), and current smoking/vaping (T1–T2). They also had no relationships at T2, and few lived outside the NYC metro area at T3. At T4, frequent drug use, violence experience, and seeking care for COVID-19 symptoms were highest in the Persistently FI trajectory.

#### Within-trajectory shifts

Aside from variation in living arrangements and location that coincided with the academic calendar and relaxing of COVID-19 countermeasures, there were few intra-trajectory trends (SM14). Over time (T1 to T4), social group involvement varied in the Never trajectory (T1: 61%, T2: 54%, T3: 44%, T4: 61%); they also had decreasing violence experience (47% to 11%), loneliness (32% to 17%), and MS-PD (59% to 50%). Loneliness also decreased in the Frequently FI trajectory (57% to 13%).

### LTFU analyses

In both LTFU models (LTFU-GEE, LTFU-Traj; SM15), those lost were more likely to be older, Seniors (vs. First-year), current smokers/vapers, MS-PD, seekers of healthcare for COVID-19 symptoms, involved in sports, and FI at T1. Frequent drug use at T1 elevated LTFU risk in the LTFU-GEE model. Factors that uniquely increased LTFU risk in the LTFU-Traj model included living with friends/others (ref: alone), Other/Multiracial identity (ref: White), and no condom use (ref: no sexual activity) at T1.

## Discussion

Our analysis with a cohort of NYC college students during the early COVID-19 pandemic (December 2020–2021) found FI was relatively rare. Factors associated with FI highlighted instances of multi-marginalization and competing financial priorities. Positive FI predictors spanned need-based financial aid, preexisting FI, low social support, frequent alcohol use, seeking care for COVID-19 symptoms, violence experience, school year (Senior), TGNC identity, and unsafe home perceptions. Unique positive correlates comprised partnerships, smoking/vaping, psychological distress, frequent drug use, and living alone or in/near NYC. Persistently FI students had low social support and partnerships and high psychological distress, need-based aid, unsafe home perceptions, smoking/vaping, drug use, TGNC representation, violence experience, and care-seeking behaviors. FI is a complex phenomenon that requires robust, multi-level and -component interventions to address the numerous vulnerabilities contributing to its occurrence.

FI was relatively stable in our sample, with approximately one in five students (range: 17%−25%) experiencing FI at each timepoint. FI estimates within this sample are slightly lower than seen in other studies conducted during the COVID-19 pandemic. One cross-sectional study with NYC college students at a public university found that 79% experienced marginal/high FI when surveyed during March-December 2020.^36^ Another cross-sectional study with City University of New York (CUNY) students, surveyed in April 2020, found that individual FI measures ranged from 18% to 50%.^37^ Another study with CUNY students found that most students experienced marginal/high FI, but its proportion remained stable before and during (prior to vs. on or after 3 March 2020) the COVID-19 pandemic (68% to 69%).^23^ A related follow-up study examining the subgroup of community college students corroborated this finding, as FI measured pre- (66%, August 2019-March 2, 2020), early-(63%, March 3, 2020 until June 30, 2020), and mid-pandemic (68%, August 23 until December 30, 2020) was proportionally high but relatively invariable.^38^ Our FI estimates may have been lower given environmental differences, as our sample arose from a private college and many of the comparative studies sampled students at public colleges/universities. Also, most of these other studies measured FI relatively earlier on in the pandemic, when responses were swift, erratic, and maldistributed. Differing FI measures and definitions could have also contributed to variation in estimates.

Across analyses, FI was most influenced by sociodemographic factors: need-based financial aid emerged in every analysis, and preexisting FI always predicted future FI. The logical relationship between need-based aid and FI is supported by other studies.^10^ Aid packages can vary, with some only offering tuition support, while others also provide funds for living expenses. Our findings suggest institutions should consider providing additional funds or wraparound services, like food stipends or subsidies, to aid packages for living expenses, especially when located in high cost-of-living areas.^5^ Since preexisting FI consistently predicted future FI in our sample, these recommendations could disrupt FI’s positive feedback loop and improve students’ mental and physical health, sleep hygiene, sociability, and scholastic performance.^1,39^ As a health equity issue, FI must be addressed to provide students with the fullest opportunity to succeed and thrive during their education and as future professionals.

Within the sociodemographic domain, TGNC identity emerged as another important consideration: TGNC-identifying students consistently had higher FI in the GEE, correlative (T1, T2, T4), and trajectory (Persistently Trajectory [T1–T4]) analyses. TGNC individuals frequently encounter discrimination from family members. Since families often provide financial/material support, higher FI among TGNC-identifying students may indicate lower familial connectivity.^21,40,41^ Even if parents wanted to help, the co-occurrence of TGNC identity and need-based aid in the GEE and trajectory analyses suggest family support might have been difficult. TGNC identity coinciding with other findings (i.e., unsafe home perceptions, low social support, violence experience, MS-PD) further underscores instances of multi-marginalization, contributing to FI and likely other negative outcomes.^42,43^ Our findings suggest that college could offer more comprehensive, inclusive, supportive services to mitigate and/or address the unique, overlapping challenges endured by TGNC students.

Students’ school year upon entering the cohort was another sociodemographic factor affecting FI: relative to First-year students, Seniors at T3 had higher FI at T4. Since most Seniors expectedly graduated after T2, this relationship may represent a subset of Seniors continuing into a fifth year due to COVID-19’s creation/magnification of innumerable stressors.^44^ Fifth-year students may need additional formal and informal supports, as aid packages rarely exceed four years.^45^ Inversely, this association may also suggest that following graduation, students were un-/under-employed due the precarious pandemic job market.^46^ In future emergencies, career services may need to offer additional materials/services on navigating employment during these unique circumstances.

Residential factors also influenced FI, with unsafe home perceptions being the most prominent (GEE, Correlate [T1, T2, T4], Persistently Trajectory [T1]). These findings indicate safety concerns potentially prevented students from acquiring/using food. Possible safety threats include cohabitants’ controlling food supplies, withholding money, restricting movement, or threating/enacting violence (reinforced by predictive and trajectory findings); hostility from other building/resident hall denizens; and/or worry of COVID-19 exposure.^47,48^ The associative overlap with need-based aid and living in/near NYC also suggests limited financial resources may have led off-campus students to live in low-income areas, potentially characterized by lower availability of food stores with nutritious options.^49,50^ Conversely, the living alone correlation (T2) may suggest other students prioritized higher rents over other expenses, including food.^21,51^ Given their interconnectedness, university efforts to address FI should also consider housing insecurity as a likely, co-occurring reality.^20^

Psychosocial and interpersonal findings likely synergize with sociodemographic and residential factors. Low social support (Predictor [T1–T2, T2–T3]; Correlate [T3]; Persistently Trajectory [T1–T3]) and MS-PD (Correlate [T1, T3]; Persistently Trajectory [T1]) findings overlap heavily with TGNC identity, unsafe home perceptions, and living in/near NYC.^52^ Violence experience (Correlate [T1]; Predictor [T2–T3]; Persistently Trajectory [T4]) also aligns with these psychosocial, sociodemographic, and residential factors.^53^ Relationship status offered somewhat conflicting findings across analyses: it was a positive correlate at T1, potentially suggesting contribution from adverse partner behaviors (e.g., control regarding food or financial resources). However, at T2, no Persistently FI students were partnered, indicating lower social support possibly contributed to FI.^54^

The emergent behavioral and healthcare factors reflect potential sources of coping and/or competing financial priorities. Frequent alcohol use and seeking care for COVID-19 symptoms both predicted FI at T2, suggesting students possibly prioritized these purchases over food. The smoking/vaping findings (Correlate [T1], Persistently Trajectory [T1, T2]) may also indicate students may have used funds for smoking/vaping supplies rather than food.^13,14,16^ Since alcohol stimulates appetite, frequent use may have also led students to consume more food than anticipated/budgeted, reasoning that may also apply to frequent drug use (Correlate [T3]) because marijuana, another appetite stimulator, was the most common drug used.^16,49^ Conversely, alcohol, drug, and cigarette/vape use may have been used to help cope with the stress brought on by FI or other related factors.^55^ While additional research is needed to confirm the directionality and motivation of these relationships, it is evident that colleges should provide stress management services in future situations to potentially minimize these behaviors. Relatedly, the directionality of the care-seeking behaviors is unexpected, for other research indicates FI is usually negatively associated with healthcare use.^26^ Perhaps the pandemic’s unprecedented circumstances led students to financially prioritize care, indicating subsidized/free healthcare may ameliorate students’ need to decide between eating and their health.

Cohort attrition may have affected our findings in several ways. Foremost, those LTFU had higher FI at T1, possibly reflecting academic attrition due to FI, a relationship that is supported by prior evidence.^56^ This attrition pattern implies FI may be higher than measured at subsequent time points and the Frequent and/or Persistent FI trajectories are potentially larger than presented in our sample. Relatedly, the statistically significant trajectory findings necessitate cautious interpretation due to low power brought on by the relatively small size of the FI trajectories. However, since most analyses with college students examined FI across only two timepoints, our trajectory analysis, despite potential limitations, shows signals of meaningful temporal, dynamic relationships that warrant deeper investigation. Relevant for the correlative and predictive findings, more students living with friends/others at T1 were excluded from the trajectory analyses, possibly explaining why cohabitant dynamics displayed an unadjusted association but did not make it into the final GEE model. Except frequent drug use (LTFU-GEE only), both LTFU models found that Seniors, smoker/vapers, MS-PD experiencers, and healthcare seekers at T1 were more likely to be lost. Along with contextualizing their null GEE findings, this missingness may also imply that these factors may have stronger associations in the predictive, correlative, and trajectory analyses. Age, racial identity, sports involvement, and condom use were associated with missingness, potentially illuminating why these factors had null associations across all analyses.

### Strengths & limitations

Our analysis has several strengths. While the current COVID-related FI literature includes examinations of one or two timepoints, our study dynamically examines FI across and within multiple unique timepoints during the COVID-19 pandemic, providing longitudinal and time-specific insights that can inform preparedness in future circumstances and contexts.^7,20,21,27–30^ Relatedly, our unique methodological approach provides novel insights into previously examined factors and emphasizes that multi-marginalized students experience poorer outcomes, reinforcing that these students must be prioritized for supplemental social, financial, and academic support. Moreover, since NYC was the epicenter of the COVID-19 pandemic in the US, our findings offer unique insights and perspectives into factors that magnified FI in an urban setting highly affected by COVID-19 countermeasures and waves. The location shift across the academic year also offers unique glimpses into risk factors that persist across collegial and home environments. Finally, by drawing from our prior research of FI and MS-PD within this same cohort, our study advances understanding of college health during emergencies and the policies, directives, and resources necessary to keep students healthy and safe during uncertain situations in this unique context.^24,25^

Despite novel insights, this analysis has some limitations. Because the questionnaire only asked two questions from the USDA’s 10-item scale, FI was likely underestimated, and severity could not be explored. Also, the survey did not measure eligibility or receipt of Supplemental Nutrition Assistance Program (SNAP) benefits, which could have affected students’ FI experiences. Due to questionnaire limitations, we also could not examine employment status, sexual orientation, or student nationality. High attrition may have affected our findings in unseen or unmeasured ways and likely led to the small size of the FI trajectories. Although this LTFU reduced analytic power, the preliminary longitudinal associations can have important implications for student wellness and institutional policies and resources, emphasizing their value. Findings may not be fully generalizable to other colleges due to underlying differences in location, socioeconomic status, demographic composition, and food store availability. Although surveys were self-administered and web-based, social desirability bias may still have affected participants’ responses.

## Conclusion

FI, a key social and structural vulnerability, disproportionately affects college students, negatively affecting their health and wellness, particularly during the COVID-19 pandemic. Among a cohort of NYC college students followed December 2020–2021, factors associated with FI primarily spanned sociodemographic (need-based financial aid, existing FI, TGNC identity), residential (unsafe home perceptions, living alone or in/near NYC), and psychosocial (low social support, psychological distress) domains. Interpersonal (violence experience, partnerships), behavioral (smoking/vaping, frequent alcohol/drug use), and healthcare (seeking care for COVID-19 symptoms) factors were also associated with FI. Despite being the smallest trajectory, Persistently FI students had low social support and partnerships; they had high psychological distress, need-based aid, unsafe home perceptions, smoking/vaping, drug use, TGNC representation, violence experience, and care-seeking behaviors. FI is underpinned by several marginalizing factors: alleviating students’ FI requires multi-pronged interventions to address contributing and competing outcomes and stressors.

## Supplementary Material

Supp 1

Supplemental data for this article can be accessed online at https://doi.org/10.1080/07448481.2026.2674845.

## Figures and Tables

**Table 1.** Sample characteristics, over time.

Characteristic	T1, *N* = 556	T2, *N* = 334	T3, *N* = 222	T4, *N* = 167	*p*-value
Age (Continuous)	20 (19–21)	20 (19–21)	20 (19–20)	20 (19–20)	0.312
Racial identity					0.727
White	63% (335/535)	63% (203/323)	63% (136/215)	65% (105/162)	
Asian/Asian American	20% (108/535)	19% (62/323)	22% (47/215)	23% (37/162)	
Other/Multiracial	17% (92/535)	18% (58/323)	15% (32/215)	12% (20/162)	
Hispanic ethnicity					0.891
No	86% (470/547)	87% (284/327)	88% (190/216)	87% (143/164)	
Yes	14% (77/547)	13% (43/327)	12% (26/216)	13% (21/164)	
School year					0.215
First-year	26% (145/555)	28% (94/334)	30% (67/222)	31% (51/167)	
Sophomore	22% (122/555)	20% (67/334)	21% (46/222)	23% (38/167)	
Junior	27% (152/555)	30% (99/334)	32% (72/222)	32% (53/167)	
Senior	25% (136/555)	22% (74/334)	17% (37/222)	15% (25/167)	
Gender identity					0.301
Cisgender woman	97% (537/556)	96% (319/334)	95% (210/222)	93% (156/167)	
Transgender/Gender non-conforming	3% (19/556)	4% (15/334)	5% (12/222)	7% (11/167)	
Received need-based financial aid					0.946
No	56% (302/539)	58% (186/321)	58% (123/213)	57% (89/157)	
Yes	44% (237/539)	42% (135/321)	42% (90/213)	43% (68/157)	
Food insecure					0.145
No	79% (438/556)	75% (252/334)	83% (185/222)	81% (135/167)	
Yes	21% (118/556)	25% (82/334)	17% (37/222)	19% (32/167)	
Lives with…					**<0.001**
Family	54% (300/552)	19% (65/334)	51% (111/217)	8% (12/159)	
Friends/Roommate/Significant other	40% (222/552)	73% (243/334)	43% (93/217)	75% (120/159)	
Alone	5% (30/552)	8% (26/334)	6% (13/217)	17% (27/159)	
Location					**<0.001**
Outside NYC metro area	45% (251/555)	17% (56/334)	40% (89/222)	7% (11/167)	
Off-campus NYC metro area	52% (290/555)	50% (166/334)	44% (97/222)	29% (49/167)	
On-campus housing	3% (14/555)	34% (112/334)	16% (36/222)	64% (107/167)	
Home perceived unsafe					0.317
No	76% (418/549)	71% (237/332)	78% (170/219)	74% (124/167)	
Yes	24% (131/549)	29% (95/332)	22% (49/219)	26% (43/167)	
Social group involvement					**<0.001**
No	39% (216/555)	51% (168/331)	61% (133/219)	40% (66/166)	
Yes	61% (339/555)	49% (163/331)	39% (86/219)	60% (100/166)	
Sports involvement					**<0.001**
No	87% (483/556)	94% (314/334)	95% (212/222)	92% (154/167)	
Yes	13% (73/556)	6% (20/334)	5% (10/222)	8% (13/167)	
Relationship status					0.645
Single	70% (387/552)	69% (230/332)	66% (146/221)	66% (111/167)	
In some form of relationship	30% (165/552)	31% (102/332)	34% (75/221)	34% (56/167)	
Experienced physical/verbal violence					**<0.001**
No	49% (258/531)	70% (223/317)	69% (144/210)	85% (121/142)	
Yes	51% (273/531)	30% (94/317)	31% (66/210)	15% (21/142)	
Condom use status					**0.024**
No sexual activity	65% (347/535)	66% (213/323)	57% (118/207)	53% (85/161)	
No condoms	16% (84/535)	16% (51/323)	17% (35/207)	17% (28/161)	
Used condoms	19% (104/535)	18% (59/323)	26% (54/207)	30% (48/161)	
Currently smokes/vapes					0.715
No	92% (513/555)	93% (309/334)	90% (198/219)	93% (154/165)	
Yes	8% (42/555)	7% (25/334)	10% (21/219)	7% (11/165)	
Frequency of alcohol use					0.243
Never/Rare	69% (381/556)	66% (219/333)	62% (138/222)	62% (103/166)	
Moderate/High	31% (175/556)	34% (114/333)	38% (84/222)	38% (63/166)	
Frequency of drug use					0.992
Never/Rare	79% (437/556)	78% (259/332)	79% (174/221)	78% (129/166)	
Monthly/Weekly/Daily	21% (119/556)	22% (73/332)	21% (47/221)	22% (37/166)	
Since 3 months ago, feel…					**<0.001**
Less lonely	39% (216/554)	45% (150/334)	56% (124/222)	63% (106/167)	
Same	20% (111/554)	23% (76/334)	21% (46/222)	20% (33/167)	
Lonelier	41% (227/554)	32% (108/334)	23% (52/222)	17% (28/167)	
Had a social support network					0.073
Yes	74% (406/549)	75% (251/333)	78% (172/220)	82% (136/165)	
Don't know	13% (69/549)	14% (48/333)	11% (24/220)	13% (21/165)	
No	13% (74/549)	10% (34/333)	11% (24/220)	5% (8/165)	
Moderate-severe psychological distress					**<0.001**
No	26% (145/552)	34% (113/333)	46% (101/220)	45% (74/165)	
Yes	74% (407/552)	66% (220/333)	54% (119/220)	55% (91/165)	
Currently using hormones					0.147
No	65% (362/555)	63% (209/333)	60% (133/222)	56% (93/166)	
Yes	35% (193/555)	37% (124/333)	40% (89/222)	44% (73/166)	
Sought care for COVID-19 symptoms					**<0.001**
No symptoms	78% (435/555)	90% (298/332)	90% (200/222)	80% (125/157)	
Sought healthcare	11% (61/555)	6% (19/332)	4% (9/222)	10% (15/157)	
Self-isolated	7% (40/555)	3% (9/332)	1% (3/222)	6% (10/157)	
No healthcare/isolation	3% (19/555)	2% (6/332)	5% (10/222)	4% (7/157)	

**Table 2.** Creation of FI trajectories.

Detailed trajectories	Number of reported instances of food insecurity				
	Zero *N* = 115	One *N* = 21	Two *N* = 14	Three *N* = 9	Four *N* = 8
	Never food insecure	Rarely food insecure	Frequently food	insecure	Persistently food insecure
Secure - Secure - Secure - Secure	**100% (115)**	0% (0)	0% (0)	0% (0)	0% (0)
Secure - Secure - Secure - Insecure	0% (0)	**43% (9)**	0% (0)	0% (0)	0% (0)
Secure - Secure - Insecure - Secure	0% (0)	24% (5)	0% (0)	0% (0)	0% (0)
Secure - Insecure - Secure - Secure	0% (0)	24% (5)	0% (0)	0% (0)	0% (0)
Insecure - Secure - Secure - Secure	0% (0)	10% (2)	0% (0)	0% (0)	0% (0)
Secure - Secure - Insecure - Insecure	0% (0)	0% (0)	**36% (5)**	0% (0)	0% (0)
Secure - Insecure - Secure - Insecure	0% (0)	0% (0)	29% (4)	0% (0)	0% (0)
Insecure - Secure - Secure - Insecure	0% (0)	0% (0)	7% (1)	0% (0)	0% (0)
Insecure - Insecure - Secure - Secure	0% (0)	0% (0)	29% (4)	0% (0)	0% (0)
Secure - Insecure - Insecure - Insecure	0% (0)	0% (0)	0% (0)	11% (1)	0% (0)
Insecure - Secure - Insecure - Insecure	0% (0)	0% (0)	0% (0)	11% (1)	0% (0)
Insecure - Insecure - Secure - Insecure	0% (0)	0% (0)	0% (0)	33% (3)	0% (0)
Insecure - Insecure - Insecure - Secure	0% (0)	0% (0)	0% (0)	**44% (4)**	0% (0)
Insecure - Insecure - Insecure - Insecure	0% (0)	0% (0)	0% (0)	0% (0)	**100% (8)**

Bolded values represent the majority for that column.

## Data Availability

The data are not publicly available given their sensitive nature. Requests to access the data used within this study should be directed to the corresponding author.
