# Supplementary material for: Food insecurity in New York City college students during the COVID-19 pandemic: a longitudinal analysis of predictors, correlates, and trajectories
Source: J Am Coll Health. Author manuscript; Available in PMC 2026 Jun 8. (PMC13245376; doi:10.1080/07448481.2026.2674845)
Supplement: Supp 1 [file NIHMS2181859-supplement-Supp_1.docx]

Supplemental Materials

Contents

[Supplemental Material 1. Measure Definitions 2](#_Toc195622754)

[Supplemental Material 2. CONSORT diagram of analytic sample 4](#_Toc195622755)

[Supplemental Material 3. Distribution of responses to individual food insecurity (FI) measures among those reporting FI 5](#_Toc195622756)

[Supplemental Material 4. All performed analyses to identify T1 predictors of food insecurity at T2 6](#_Toc195622757)

[Supplemental Material 5. All performed analyses to identify T2 predictors of food insecurity at T3 8](#_Toc195622758)

[Supplemental Material 6. All performed analyses to identify T3 predictors of food insecurity at T4 10](#_Toc195622759)

[Supplemental Material 7. All performed analyses to identify factors marginally associated with food insecurity in a Generalized Estimating Equations (GEE) model (N=167) 12](#_Toc195622760)

[Supplemental Material 8. All performed analyses to identify correlates of food insecurity at T1 14](#_Toc195622761)

[Supplemental Material 8A. Without violence fitted in model 14](#_Toc195622762)

[Supplemental Material 8B. With violence fitted in model 16](#_Toc195622763)

[Supplemental Material 9. All performed analyses to identify correlates of food insecurity at T2 18](#_Toc195622764)

[Supplemental Material 10. All performed analyses to identify correlates of food insecurity at T3 20](#_Toc195622765)

[Supplemental Material 11. All performed analyses to identify correlates of food insecurity at T4 22](#_Toc195622766)

[Supplemental Material 12. Trajectory sample characteristics, over time 24](#_Toc195622767)

[Supplemental Material 13. Characteristics of Food Insecurity Trajectories, by Time 26](#_Toc195622768)

[Supplemental Material 14. Characteristics of Food Insecurity Trajectories, across Time 28](#_Toc195622769)

[Supplemental Material 15. Lost-to-follow-up (LTFU) analyses 30](#_Toc195622770)

# Supplemental Material 1. Measure Definitions

| **Variable** | **Type** | **Temporality** | **Definition** |
| --- | --- | --- | --- |
| ***Sociodemographic domain^†^*** | | | |
| Age | Discrete | T1 only | Age in years |
| Racial identity | Polytomous | T1 only | White vs. Asian/Asian American vs. Multiracial/Other  White and Asian/Asian American included exclusive responses to these self-reported categorizations.  Multiracial/Other captures responses to 1) more than one racial category, 2) Black/African American (due to low representation), or 3) Other (e.g., Middle Eastern, Jewish). Of those within this category at T1 (N=92), 50% (n=46) were multiracial, 26% (n=24) were Black/African only, and 24% (n=22) were Other. |
| Hispanic ethnicity | Binary | T1 only | Hispanic ethnicity vs. Not. |
| School year | Polytomous | T1 only | Year of matriculation when enrolled into the cohort: First-year vs. Sophomore vs. Junior vs. Senior |
| Gender identity | Binary | Contemporaneous | Cisgender women vs. Transgender/Gender non-conforming students  Due to low representation (<5%), transgender and gender non-conforming respondents were combined. This response category included non-binary, transgender, gender non-conforming, questioning, agender, and demigender identities. |
| Need-based financial aid | Binary | Contemporaneous | Received need-based financial aid vs. Not. |
| Food not lasting | Binary | Contemporaneous | In the last 30 days, the food they bought often or sometimes (vs. never) didn’t last, and they didn’t have money to get more. |
| No balanced meals | Binary | Contemporaneous | In the last 30 days, they often or sometimes (vs. never) could not afford to eat balanced meals. |
| Food insecurity | Binary | Contemporaneous | Positive indication for either food not lasting or no balanced meals. The reference category only included those responding “never” to both factors.^(1-3)^ |
| ***Residential domain*** | | | |
| Living situation | Polytomous | Contemporaneous | Living with Family vs. Friends, Roommate(s)s, Suitemates, or Significant Others vs. Alone. If a respondent said they lived with both friends and family, we randomized them to either category. |
| Location | Polytomous | Contemporaneous | On-campus vs. Off-campus in the New York City metro area vs. Outside the New York City metro area. |
| Home perceived unsafe | Binary | Contemporaneous | Responded Somewhat/Not very/Not Safe when asked, “How safe do you feel in your current home?” The reference category included responses of Very Safe. |
| **Interpersonal domain** | | | |
| Social group involvement | Binary | Contemporaneous | Involved in >1 social group (Peer educator/advisor, Resident assistant, Student government, Dance/theater group, Musical group, Teaching assistant, Other) vs. Not |
| Sports group involvement | Binary | Contemporaneous | Involved in >1 sports group (Club sports, Intramurals, Varsity) vs. Not |
| Relationship status | Binary | Contemporaneous | In a relationship vs. Single.  Relationships included married or engaged respondents, as well as those in one, multiple, or complex relationships. Complex relationships comprised situationships, casual dating, exclusive and non-exclusive partnerships, and "it's complicated." |
| Experience of violence | Binary | Contemporaneous | In the last 30 days, a partner, spouse, or cohabitant verbally (yelled or said things that made them feel bad about themselves, embarrassed in front of others, or frightened them) or physically (push, grab, hit, slap, kick, or throw things at them) attacked them. Respondents were only asked these questions if they were in a relationship or lived with someone. |
| Condom use | Polytomous | Contemporaneous | Those sexually active in the last 3 months were asked if they used or did not use condoms. We included Not sexually active as the reference category to include the entire sample.  Transformed categories: Used condoms vs. Did not use condoms vs. Not sexually active |
| ***Behavioral domain^‡^*** | | | |
| Current smoking/vaping | Binary | Contemporaneous | Currently smoked or vaped. |
| Alcohol consumption | Binary | Contemporaneous | Alcohol consumption in the past 30 days, coded as low (never or <1-2 days a week), moderate (1-2 days a week), and high (3-4 days a week, daily or almost every day, more than once a day). The moderate and high were combined because high always had low representation. |
| Drug use | Binary | Contemporaneous | Drug use in the past 30 days, coded as none/rare (never or less than once a month), moderate (1-3 days a month), and high (1-2 days a week, 3-4 days a week, daily or almost every day). We collapsed moderate and high because each always had low representation. |
| ***Psychosocial domain*** | | | |
| Loneliness | Polytomous | Contemporaneous | Compared to 3 months ago, felt Lonelier vs. Same vs. Less lonely. |
| Social support | Polytomous / Binary | Contemporaneous | Believing they had a social support network where they lived: No vs. Uncertain vs. Yes. The trajectory analyses used a dichotomous version (No/Uncertain vs. Yes) to streamline interpretations. |
| Moderate-Severe psychological distress | Binary | Contemporaneous | We measured psychological distress using the Kessler-6 (K6) scale. The K6 captures feelings of nervousness, hopelessness, restlessness, sadness, lethargy, and worthlessness over the last 30 days. For each measure, participants noted the frequency of these feelings using a 0-4 scale (0 = None, 1 = A little, 2 = Some, 3 = Most, and 4 = All of the time). When summed, the scale’s score ranged from 0-24.^(4)^ The K6 scale exhibited good reliability in the overall sample (Cronbach’s α= 0.84) and trajectory sample (Cronbach’s α= 0.84).  We created a binary variable to indicate those who scored as mild/moderate/severe (scores: ≥8) vs. no/low (scores: <8) distress.^(5)^ |
| ***Healthcare domain*** | | | |
| Currently using hormones | Binary | Contemporaneous | Currently using hormones (birth control, gender affirming therapy, hormone replacement therapy) vs. Not |
| Sought care for COVID-19 symptoms | Binary | Contemporaneous | Those who had experienced COVID-19 symptoms were asked what responsive actions they took. We categorized actions as Sought healthcare (Telemedicine, Non-urgent care visit, Urgent care visit, Emergency room, Hospital admission, ICU admission), Self-isolated, and No healthcare / isolation. We also included a No COVID-19 symptoms category. The reference category was No symptoms because the other responses had financial implications attached to them.  Transformed categories: No healthcare/isolation vs. Self-isolated vs. Sought healthcare vs. No symptoms |

Unless specified, Don’t know responses and Non-responses were coded as missing.

† Questions were drawn from NIH’s typical reporting guidelines.

‡ Alcohol and drug use behavior reflect standardized questions used by the National Institute on Alcohol Abuse and Alcoholism (NIAAA) and National Institute on Drug Abuse (NIDA), respectively.

# Supplemental Material 2. CONSORT diagram of analytic sample


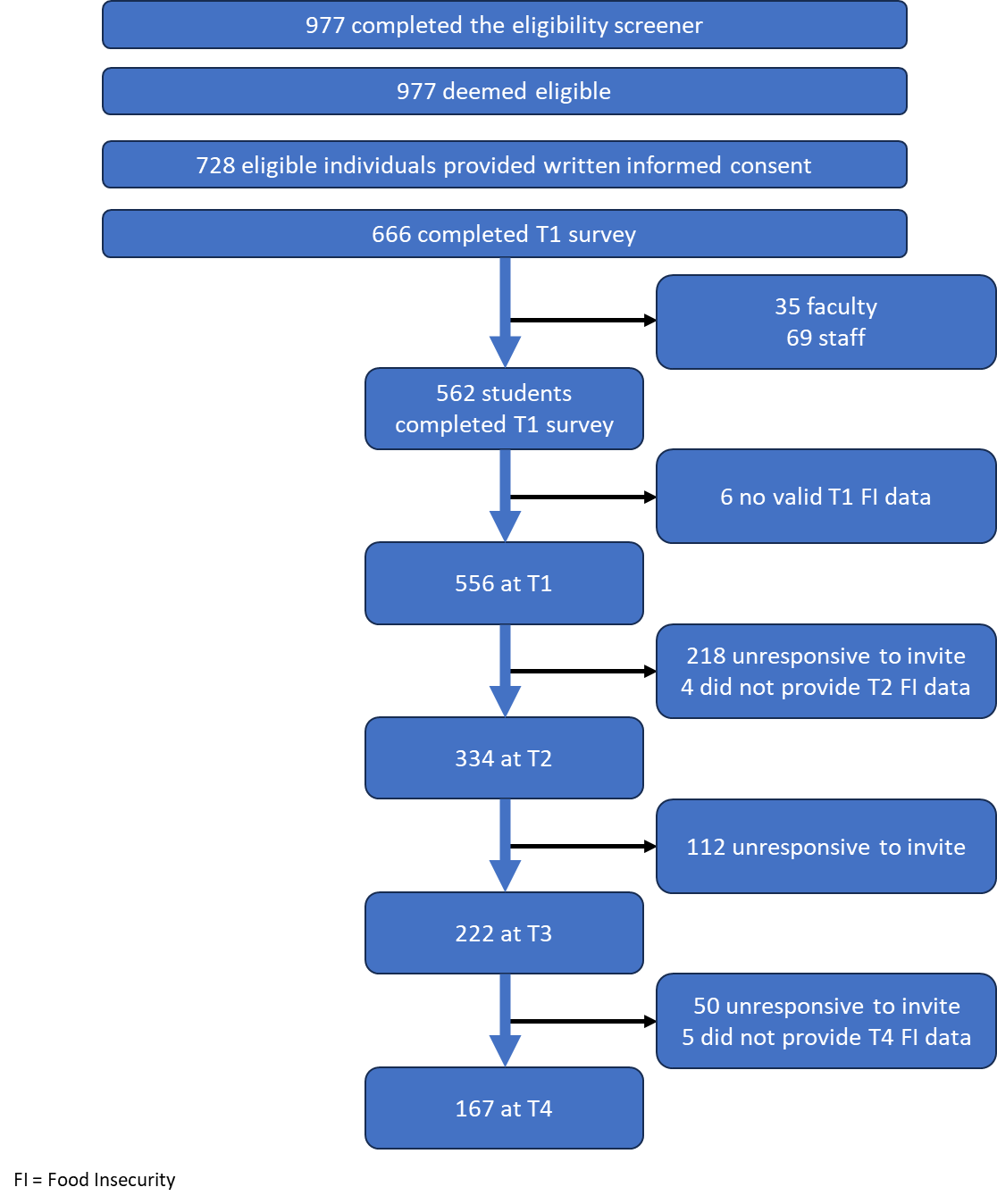


# Supplemental Material 3. Distribution of responses to individual food insecurity (FI) measures among those reporting FI


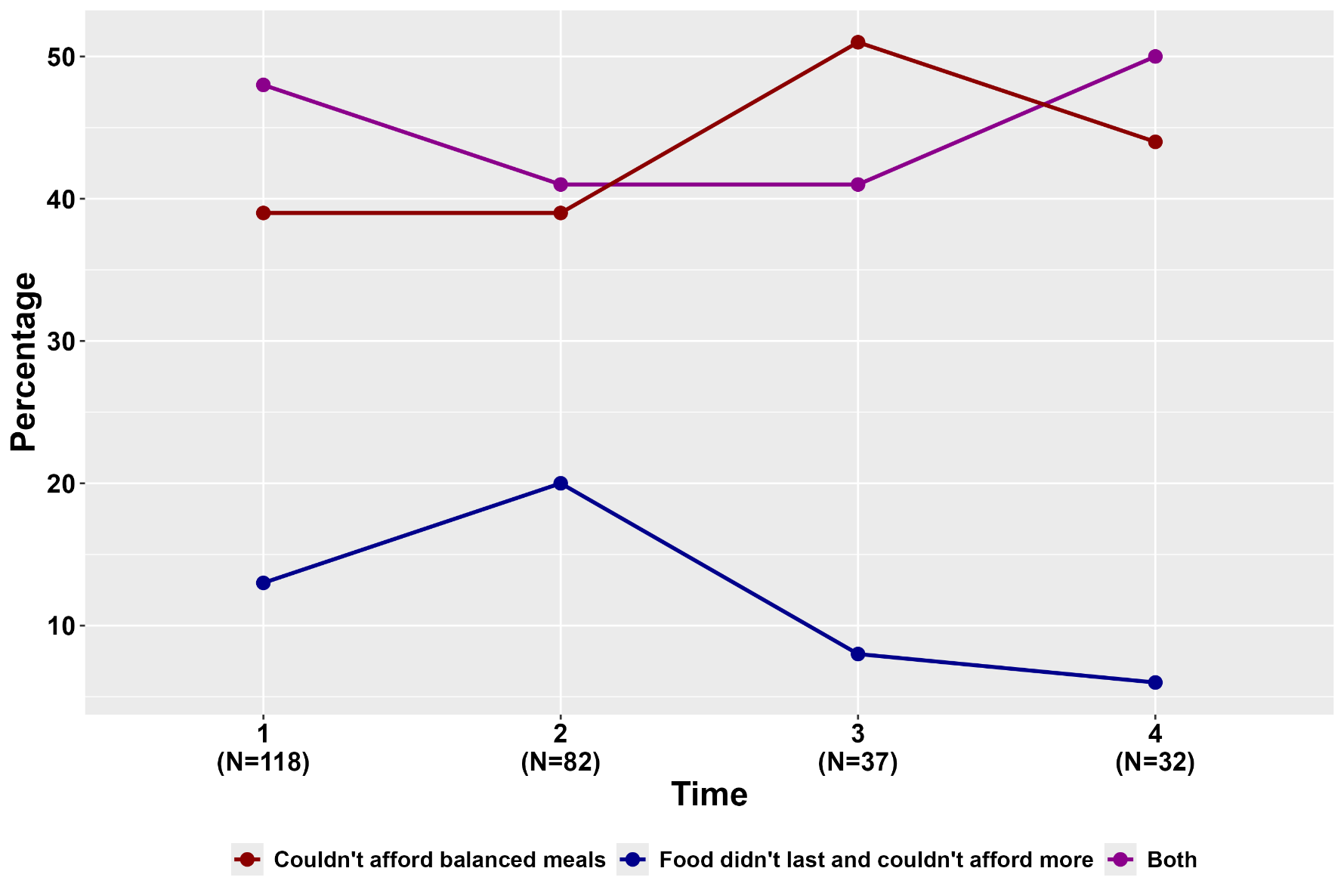


Mutually exclusive proportions are reported. Sample sizes reflect those affirmatively coded for FI at the respective timepoints

# Supplemental Material 4. All performed analyses to identify T1 predictors of food insecurity at T2

|  | **Food insecure** | | **Minimally Adjusted** | | **PAM 1** | | **PAM 2** | | **PAM 3** | | **PAM 4** | | **Final model** | |
| --- | --- | --- | --- | --- | --- | --- | --- | --- | --- | --- | --- | --- | --- | --- |
| **Characteristic** | **No, N = 252** | **Yes, N = 82** | **aRR [95% CI]** | **p-value** | **ARR [95% CI]** | **p-value** | **ARR [95% CI]** | **p-value** | **ARR [95% CI]** | **p-value** | **ARR [95% CI]** | **p-value** | **ARR [95% CI]** | **p-value** |
| **Age (Continuous)** | 20 (19-21) | 20 (19-21) | 1.07 [0.95-1.20] | 0.288 |  |  |  |  |  |  |  |  |  |  |
| **Racial identity** |  |  |  |  |  |  |  |  |  |  |  |  |  |  |
| *White* | 78% (158/203) | 22% (45/203) | 1.00 [ref] |  |  |  |  |  |  |  |  |  |  |  |
| *Asian/Asian American* | 74% (46/62) | 26% (16/62) | 1.26 [0.81-1.95] | 0.308 |  |  |  |  |  |  |  |  |  |  |
| *Other/Multiracial* | 67% (39/58) | 33% (19/58) | 0.95 [0.70-1.30] | 0.767 |  |  |  |  |  |  |  |  |  |  |
| **Hispanic ethnicity** |  |  |  |  |  |  |  |  |  |  |  |  |  |  |
| *No* | 76% (215/284) | 24% (69/284) | 1.00 [ref] |  |  |  |  |  |  |  |  |  |  |  |
| *Yes* | 74% (32/43) | 26% (11/43) | 0.89 [0.59-1.33] | 0.555 |  |  |  |  |  |  |  |  |  |  |
| **School year** |  |  |  |  |  |  |  |  |  |  |  |  |  |  |
| *First-year* | 79% (74/94) | 21% (20/94) | 1.00 [ref] |  |  |  |  |  |  |  |  |  |  |  |
| *Sophomore* | 78% (52/67) | 22% (15/67) | 1.18 [0.74-1.88] | 0.489 |  |  |  |  |  |  |  |  |  |  |
| *Junior* | 75% (74/99) | 25% (25/99) | 1.11 [0.74-1.67] | 0.599 |  |  |  |  |  |  |  |  |  |  |
| *Senior* | 70% (52/74) | 30% (22/74) | 1.23 [0.82-1.85] | 0.326 |  |  |  |  |  |  |  |  |  |  |
| **Gender Identity** |  |  |  |  |  |  |  |  |  |  |  |  |  |  |
| *Cisgender woman* | 77% (248/323) | 23% (75/323) | 1.00 [ref] |  |  |  |  |  |  |  |  |  |  |  |
| *Transgender/Gender non-conforming* | 36% (4/11) | 64% (7/11) | 1.29 [0.95-1.74] | 0.105 |  |  |  |  |  |  |  |  |  |  |
| **Received need-based financial aid** |  |  |  |  |  |  |  |  |  |  |  |  |  |  |
| *No* | 86% (160/186) | 14% (26/186) | 1.00 [ref] |  | 1.00 [ref] |  | 1.00 [ref] |  | 1.00 [ref] |  | 1.00 [ref] |  | 1.00 [ref] |  |
| *Yes* | 60% (83/138) | 40% (55/138) | 1.47 [0.99-2.17] | 0.055 | 1.51 [0.99-2.31] | 0.055 | 1.52 [1.03-2.25] | **0.035** | 1.52 [1.03-2.25] | **0.035** | 1.52 [1.03-2.26] | **0.036** | 1.50 [1.01-2.22] | **0.044** |
| **Food insecure** |  |  |  |  |  |  |  |  |  |  |  |  |  |  |
| *No* | 88% (242/274) | 12% (32/274) |  |  | 1.00 [ref] |  | 1.00 [ref] |  | 1.00 [ref] |  | 1.00 [ref] |  | 1.00 [ref] |  |
| *Yes* | 17% (10/60) | 83% (50/60) |  |  | 5.77 [3.94-8.45] | **<0.001** | 5.67 [3.90-8.25] | **<0.001** | 5.94 [4.07-8.67] | **<0.001** | 5.85 [3.98-8.59] | **<0.001** | 5.98 [4.06-8.80] | **<0.001** |
| **Lives with...** |  |  |  |  |  |  |  |  |  |  |  |  |  |  |
| *Family* | 79% (145/184) | 21% (39/184) | 1.00 [ref] |  | 1.00 [ref] |  | 1.00 [ref] |  | 1.00 [ref] |  | 1.00 [ref] |  |  |  |
| *Friends/Roommate(s)/Significant other* | 74% (93/126) | 26% (33/126) | 1.10 [0.81-1.50] | 0.553 | 1.14 [0.81-1.61] | 0.453 | 1.17 [0.84-1.63] | 0.356 | 1.12 [0.80-1.57] | 0.499 | 1.10 [0.79-1.54] | 0.566 |  |  |
| *Alone* | 57% (12/21) | 43% (9/21) | 1.62 [1.02-2.58] | **0.041** | 2.02 [1.15-3.56] | **0.015** | 2.01 [1.15-3.50] | **0.014** | 1.83 [1.07-3.15] | **0.028** | 1.62 [0.98-2.67] | 0.059 |  |  |
| **Location** |  |  |  |  |  |  |  |  |  |  |  |  |  |  |
| *Outside NYC metro area* | 81% (130/161) | 19% (31/161) | 1.00 [ref] |  |  |  |  |  |  |  |  |  |  |  |
| *Off-campus NYC metro area* | 72% (118/164) | 28% (46/164) | 1.10 [0.80-1.52] | 0.567 |  |  |  |  |  |  |  |  |  |  |
| *On-campus housing* | 44% (4/9) | 56% (5/9) | 1.38 [0.88-2.17] | 0.165 |  |  |  |  |  |  |  |  |  |  |
| **Home perceived unsafe** |  |  |  |  |  |  |  |  |  |  |  |  |  |  |
| *No* | 81% (199/246) | 19% (47/246) | 1.00 [ref] |  |  |  |  |  |  |  |  |  |  |  |
| *Yes* | 61% (51/83) | 39% (32/83) | 1.23 [0.91-1.66] | 0.178 |  |  |  |  |  |  |  |  |  |  |
| **Social group involvement** |  |  |  |  |  |  |  |  |  |  |  |  |  |  |
| *No* | 75% (96/128) | 25% (32/128) | 1.00 [ref] |  |  |  |  |  |  |  |  |  |  |  |
| *Yes* | 76% (155/205) | 24% (50/205) | 0.97 [0.72-1.31] | 0.859 |  |  |  |  |  |  |  |  |  |  |
| **Sports involvement** |  |  |  |  |  |  |  |  |  |  |  |  |  |  |
| *No* | 75% (220/293) | 25% (73/293) | 1.00 [ref] |  |  |  |  |  |  |  |  |  |  |  |
| *Yes* | 78% (32/41) | 22% (9/41) | 0.84 [0.56-1.26] | 0.394 |  |  |  |  |  |  |  |  |  |  |
| **Relationship status** |  |  |  |  |  |  |  |  |  |  |  |  |  |  |
| *Single* | 77% (177/230) | 23% (53/230) | 1.00 [ref] |  |  |  |  |  |  |  |  |  |  |  |
| *In some form of relationship* | 73% (74/102) | 27% (28/102) | 1.02 [0.76-1.37] | 0.898 |  |  |  |  |  |  |  |  |  |  |
| **Experienced physical/verbal violence** |  |  |  |  |  |  |  |  |  |  |  |  |  |  |
| *No* | 79% (127/161) | 21% (34/161) | 1.00 [ref] |  |  |  |  |  |  |  |  |  |  |  |
| *Yes* | 73% (114/156) | 27% (42/156) | 0.93 [0.68-1.27] | 0.635 |  |  |  |  |  |  |  |  |  |  |
| **Condom use status** |  |  |  |  |  |  |  |  |  |  |  |  |  |  |
| *No sexual activity* | 79% (171/216) | 21% (45/216) | 1.00 [ref] |  | 1.00 [ref] |  |  |  |  |  |  |  |  |  |
| *No condoms* | 74% (37/50) | 26% (13/50) | 1.05 [0.70-1.56] | 0.822 | 1.09 [0.66-1.79] | 0.740 |  |  |  |  |  |  |  |  |
| *Used condoms* | 64% (37/58) | 36% (21/58) | 1.44 [1.00-2.08] | **0.050** | 1.17 [0.78-1.76] | 0.451 |  |  |  |  |  |  |  |  |
| **Currently smokes/vapes** |  |  |  |  |  |  |  |  |  |  |  |  |  |  |
| *No* | 77% (244/316) | 23% (72/316) | 1.00 [ref] |  |  |  |  |  |  |  |  |  |  |  |
| *Yes* | 41% (7/17) | 59% (10/17) | 1.35 [0.89-2.04] | 0.155 |  |  |  |  |  |  |  |  |  |  |
| **Frequency of alcohol use** |  |  |  |  |  |  |  |  |  |  |  |  |  |  |
| *Never/Rare* | 77% (178/231) | 23% (53/231) | 1.00 [ref] |  | 1.00 [ref] |  | 1.00 [ref] |  | 1.00 [ref] |  | 1.00 [ref] |  | 1.00 [ref] |  |
| *Moderate/High* | 72% (74/103) | 28% (29/103) | 1.31 [0.97-1.76] | 0.077 | 1.39 [0.93-2.08] | 0.107 | 1.47 [1.01-2.14] | **0.044** | 1.54 [1.04-2.27] | **0.03** | 1.49 [1.02-2.18] | **0.039** | 1.55 [1.08-2.22] | **0.018** |
| **Frequency of drug use** |  |  |  |  |  |  |  |  |  |  |  |  |  |  |
| *Never/Rare* | 78% (209/269) | 22% (60/269) | 1.00 [ref] |  |  |  |  |  |  |  |  |  |  |  |
| *Monthly/Weekly/Daily* | 66% (43/65) | 34% (22/65) | 1.21 [0.90-1.63] | 0.203 |  |  |  |  |  |  |  |  |  |  |
| **Since 3 months ago, feel...** |  |  |  |  |  |  |  |  |  |  |  |  |  |  |
| *Less lonely* | 78% (106/136) | 22% (30/136) | 1.00 [ref] |  | 1.00 [ref] |  | 1.00 [ref] |  | 1.00 [ref] |  |  |  |  |  |
| *Same* | 82% (50/61) | 18% (11/61) | 0.87 [0.55-1.36] | 0.534 | 0.92 [0.55-1.53] | 0.75 | 0.86 [0.53-1.39] | 0.548 | 0.92 [0.56-1.49] | 0.725 |  |  |  |  |
| *Lonelier* | 70% (95/136) | 30% (41/136) | 1.37 [1.00-1.87] | 0.051 | 1.41 [0.95-2.10] | 0.089 | 1.35 [0.91-1.99] | 0.131 | 1.37 [0.93-2.01] | 0.110 |  |  |  |  |
| **Had a social support network** |  |  |  |  |  |  |  |  |  |  |  |  |  |  |
| *Yes* | 81% (190/235) | 19% (45/235) | 1.00 [ref] |  | 1.00 [ref] |  | 1.00 [ref] |  | 1.00 [ref] |  | 1.00 [ref] |  | 1.00 [ref] |  |
| *Uncertain* | 62% (29/47) | 38% (18/47) | 1.58 [1.06-2.36] | **0.025** | 1.60 [0.98-2.62] | 0.063 | 1.59 [1.00-2.51] | **0.049** | 1.58 [0.99-2.52] | 0.057 | 1.74 [1.12-2.70] | **0.013** | 1.76 [1.12-2.75] | **0.013** |
| *No* | 63% (30/48) | 38% (18/48) | 1.35 [0.95-1.94] | 0.098 | 1.38 [0.92-2.06] | 0.119 | 1.37 [0.92-2.06] | 0.123 | 1.46 [0.98-2.19] | 0.063 | 1.58 [1.08-2.30] | **0.019** | 1.60 [1.10-2.32] | **0.015** |
| **Moderate-severe psychological distress** |  |  |  |  |  |  |  |  |  |  |  |  |  |  |
| *No* | 87% (87/100) | 13% (13/100) | 1.00 [ref] |  | 1.00 [ref] |  | 1.00 [ref] |  |  |  |  |  |  |  |
| *Yes* | 71% (164/232) | 29% (68/232) | 1.54 [0.93-2.53] | 0.093 | 1.37 [0.81-2.31] | 0.238 | 1.42 [0.87-2.33] | 0.158 |  |  |  |  |  |  |
| **Currently using hormones** |  |  |  |  |  |  |  |  |  |  |  |  |  |  |
| *No* | 76% (165/218) | 24% (53/218) | 1.00 [ref] |  |  |  |  |  |  |  |  |  |  |  |
| *Yes* | 75% (87/116) | 25% (29/116) | 1.15 [0.84-1.58] | 0.390 |  |  |  |  |  |  |  |  |  |  |
| **Sought care for COVID-19 symptoms** |  |  |  |  |  |  |  |  |  |  |  |  |  |  |
| *No symptoms* | 78% (206/263) | 22% (57/263) | 1.00 [ref] |  | 1.00 [ref] |  | 1.00 [ref] |  | 1.00 [ref] |  | 1.00 [ref] |  | 1.00 [ref] |  |
| *Sought healthcare* | 71% (24/34) | 29% (10/34) | 1.65 [1.03-2.64] | **0.035** | 1.76 [1.11-2.78] | **0.016** | 1.72 [1.07-2.76] | **0.025** | 1.77 [1.09-2.86] | **0.02** | 1.70 [1.05-2.76] | **0.032** | 1.64 [1.01-2.67] | **0.046** |
| *Self-isolated* | 58% (14/24) | 42% (10/24) | 1.77 [1.04-3.01] | **0.035** | 1.37 [0.76-2.46] | 0.3 | 1.33 [0.75-2.36] | 0.323 | 1.41 [0.80-2.47] | 0.232 | 1.52 [0.88-2.64] | 0.134 | 1.47 [0.84-2.59] | 0.18 |
| *No healthcare/isolation* | 67% (8/12) | 33% (4/12) | 1.27 [0.77-2.09] | 0.344 | 1.37 [0.65-2.86] | 0.406 | 1.47 [0.77-2.79] | 0.24 | 1.17 [0.64-2.13] | 0.615 | 1.24 [0.71-2.16] | 0.45 | 1.20 [0.69-2.08] | 0.526 |

PAM = Preliminary Adjusted Model, aRR = Minimally Adjusted Risk Ratio, ARR = Adjusted Risk Ratio

Minimally adjusted means only controlling for current food insecurity

Green coloring used to highlight factors with p≤0.05

# Supplemental Material 5. All performed analyses to identify T2 predictors of food insecurity at T3

|  | **Food insecure** | | **Minimally Adjusted** | | **Without violence fitted in model** | | **With violence fitted in model** | | | |
| --- | --- | --- | --- | --- | --- | --- | --- | --- | --- | --- |
|  |  |  |  |  | **Final Model** | | **PAM 1** | **PAM 1 (with Violence)** | **Final model** | |
| **Characteristic** | **No, N = 185** | **Yes, N = 37** | **aRR [95% CI]** | **p-value** | **ARR [95% CI]** | **p-value** | **ARR [95% CI]** | **p-value** | **ARR [95% CI]** | **p-value** |
| **Age (Continuous)** | 20 (19-20) | 20 (19-20) | 0.92 [0.72-1.17] | 0.501 |  |  |  |  |  |  |
| **Racial identity** |  |  |  |  |  |  |  |  |  |  |
| *White* | 85% (115/136) | 15% (21/136) | 1.00 [ref] |  |  |  |  |  |  |  |
| *Asian/Asian American* | 87% (41/47) | 13% (6/47) | 0.75 [0.35-1.62] | 0.469 |  |  |  |  |  |  |
| *Other/Multiracial* | 72% (23/32) | 28% (9/32) | 1.54 [0.79-3.00] | 0.204 |  |  |  |  |  |  |
| **Hispanic ethnicity** |  |  |  |  |  |  |  |  |  |  |
| *No* | 84% (160/190) | 16% (30/190) | 1.00 [ref] |  |  |  |  |  |  |  |
| *Yes* | 77% (20/26) | 23% (6/26) | 1.61 [0.90-2.87] | 0.109 |  |  |  |  |  |  |
| **School year** |  |  |  |  |  |  |  |  |  |  |
| *First-year* | 82% (55/67) | 18% (12/67) | 1.00 [ref] |  |  |  |  |  |  |  |
| *Sophomore* | 85% (39/46) | 15% (7/46) | 0.83 [0.37-1.88] | 0.655 |  |  |  |  |  |  |
| *Junior* | 83% (60/72) | 17% (12/72) | 0.84 [0.44-1.60] | 0.595 |  |  |  |  |  |  |
| *Senior* | 84% (31/37) | 16% (6/37) | 0.80 [0.34-1.89] | 0.615 |  |  |  |  |  |  |
| **Gender Identity** |  |  |  |  |  |  |  |  |  |  |
| *Cisgender woman* | 84% (178/211) | 16% (33/211) | 1.00 [ref] |  |  |  |  |  |  |  |
| *Transgender/Gender non-conforming* | 64% (7/11) | 36% (4/11) | 1.41 [0.80-2.51] | 0.235 |  |  |  |  |  |  |
| **Received need-based financial aid** |  |  |  |  |  |  |  |  |  |  |
| *No* | 91% (112/123) | 9% (11/123) | 1.00 [ref] |  | 1.00 [ref] |  | 1.00 [ref] |  | 1.00 [ref] |  |
| *Yes* | 73% (66/90) | 27% (24/90) | 1.97 [1.00-3.91] | 0.051 | 2.00 [1.03-3.88] | **0.041** | 2.20 [1.07-4.52] | **0.032** | 2.30 [1.11-4.76] | **0.025** |
| **Food insecure** |  |  |  |  |  |  |  |  |  |  |
| *No* | 91% (163/180) | 9% (17/180) |  |  | 1.00 [ref] |  | 1.00 [ref] |  | 1.00 [ref] |  |
| *Yes* | 52% (22/42) | 48% (20/42) |  |  | 3.85 [2.08-7.12] | **<0.001** | 3.45 [1.85-6.43] | **<0.001** | 3.56 [1.92-6.61] | **<0.001** |
| **Lives with...** |  |  |  |  |  |  |  |  |  |  |
| *Family* | 81% (38/47) | 19% (9/47) | 1.00 [ref] |  |  |  |  |  |  |  |
| *Friends/Roommate(s)/Significant other* | 86% (136/159) | 14% (23/159) | 0.72 [0.38-1.36] | 0.310 |  |  |  |  |  |  |
| *Alone* | 69% (11/16) | 31% (5/16) | 0.87 [0.36-2.08] | 0.750 |  |  |  |  |  |  |
| **Location** |  |  |  |  |  |  |  |  |  |  |
| *Outside NYC metro area* | 87% (34/39) | 13% (5/39) | 1.00 [ref] |  |  |  |  |  |  |  |
| *Off-campus NYC metro area* | 84% (87/104) | 16% (17/104) | 1.00 [0.41-2.41] | 0.997 |  |  |  |  |  |  |
| *On-campus housing* | 81% (64/79) | 19% (15/79) | 1.35 [0.56-3.24] | 0.503 |  |  |  |  |  |  |
| **Home perceived unsafe** |  |  |  |  |  |  |  |  |  |  |
| *No* | 85% (132/156) | 15% (24/156) | 1.00 [ref] |  |  |  |  |  |  |  |
| *Yes* | 82% (53/65) | 18% (12/65) | 1.04 [0.59-1.83] | 0.886 |  |  |  |  |  |  |
| **Social group involvement** |  |  |  |  |  |  |  |  |  |  |
| *No* | 84% (96/114) | 16% (18/114) | 1.00 [ref] |  |  |  |  |  |  |  |
| *Yes* | 83% (87/105) | 17% (18/105) | 1.00 [0.58-1.73] | 0.993 |  |  |  |  |  |  |
| **Sports involvement** |  |  |  |  |  |  |  |  |  |  |
| *No* | 83% (172/208) | 17% (36/208) | 1.00 [ref] |  |  |  |  |  |  |  |
| *Yes* | 93% (13/14) | 7% (1/14) | 0.57 [0.12-2.68] | 0.478 |  |  |  |  |  |  |
| **Relationship status** |  |  |  |  |  |  |  |  |  |  |
| *Single* | 81% (123/151) | 19% (28/151) | 1.00 [ref] |  |  |  |  |  |  |  |
| *In some form of relationship* | 89% (62/70) | 11% (8/70) | 0.57 [0.28-1.14] | 0.111 |  |  |  |  |  |  |
| **Experienced physical/verbal violence** |  |  |  |  |  |  |  |  |  |  |
| *No* | 88% (130/147) | 12% (17/147) | 1.00 [ref] |  |  |  | 1.00 [ref] |  | 1.00 [ref] |  |
| *Yes* | 76% (51/67) | 24% (16/67) | 1.79 [1.01-3.15] | **0.045** |  |  | 1.65 [0.92-2.96] | 0.095 | 1.81 [1.02-3.21] | **0.044** |
| **Condom use status** |  |  |  |  |  |  |  |  |  |  |
| *No sexual activity* | 83% (119/144) | 17% (25/144) | 1.00 [ref] |  |  |  |  |  |  |  |
| *No condoms* | 87% (26/30) | 13% (4/30) | 0.54 [0.22-1.34] | 0.181 |  |  |  |  |  |  |
| *Used condoms* | 90% (38/42) | 10% (4/42) | 0.58 [0.22-1.58] | 0.291 |  |  |  |  |  |  |
|  |  |  |  |  |  |  |  |  |  |  |
| **Currently smokes/vapes** |  |  |  |  |  |  |  |  |  |  |
| *No* | 85% (175/207) | 15% (32/207) | 1.00 [ref] |  |  |  |  |  |  |  |
| *Yes* | 67% (10/15) | 33% (5/15) | 1.42 [0.84-2.38] | 0.188 |  |  |  |  |  |  |
| **Frequency of alcohol use** |  |  |  |  |  |  |  |  |  |  |
| *Never/Rare* | 82% (127/155) | 18% (28/155) | 1.00 [ref] |  |  |  |  |  |  |  |
| *Moderate/High* | 86% (57/66) | 14% (9/66) | 0.81 [0.43-1.54] | 0.529 |  |  |  |  |  |  |
| **Frequency of drug use** |  |  |  |  |  |  |  |  |  |  |
| *Never/Rare* | 84% (153/182) | 16% (29/182) | 1.00 [ref] |  |  |  |  |  |  |  |
| *Monthly/Weekly/Daily* | 79% (31/39) | 21% (8/39) | 1.16 [0.62-2.16] | 0.652 |  |  |  |  |  |  |
| **Since 3 months ago, feel...** |  |  |  |  |  |  |  |  |  |  |
| *Less lonely* | 83% (84/101) | 17% (17/101) | 1.00 [ref] |  |  |  |  |  |  |  |
| *Same* | 82% (42/51) | 18% (9/51) | 0.92 [0.49-1.75] | 0.809 |  |  |  |  |  |  |
| *Lonelier* | 84% (59/70) | 16% (11/70) | 0.95 [0.50-1.81] | 0.873 |  |  |  |  |  |  |
| **Had a social support network** |  |  |  |  |  |  |  |  |  |  |
| *Yes* | 86% (146/169) | 14% (23/169) | 1.00 [ref] |  | 1.00 [ref] |  | 1.00 [ref] |  |  |  |
| *Uncertain* | 84% (26/31) | 16% (5/31) | 1.04 [0.49-2.21] | 0.912 | 0.97 [0.48-1.99] | 0.941 | 1.00 [0.48-2.06] | 0.992 |  |  |
| *No* | 59% (13/22) | 41% (9/22) | 2.24 [1.15-4.36] | **0.018** | 2.02 [1.05-3.88] | **0.035** | 1.78 [0.87-3.64] | 0.111 |  |  |
| **Moderate-severe psychological distress** |  |  |  |  |  |  |  |  |  |  |
| *No* | 89% (64/72) | 11% (8/72) | 1.00 [ref] |  |  |  |  |  |  |  |
| *Yes* | 81% (121/150) | 19% (29/150) | 1.40 [0.69-2.82] | 0.352 |  |  |  |  |  |  |
| **Currently using hormones** |  |  |  |  |  |  |  |  |  |  |
| *No* | 82% (113/138) | 18% (25/138) | 1.00 [ref] |  |  |  |  |  |  |  |
| *Yes* | 87% (72/83) | 13% (11/83) | 0.74 [0.40-1.40] | 0.357 |  |  |  |  |  |  |
| **Sought care for COVID-19 symptoms** |  |  |  |  |  |  |  |  |  |  |
| *No symptoms* | 84% (166/198) | 16% (32/198) | † |  |  |  |  |  |  |  |
| *Sought healthcare* | 75% (9/12) | 25% (3/12) | † |  |  |  |  |  |  |  |
| *Self-isolated* | 100% (5/5) | 0% (0/5) | † |  |  |  |  |  |  |  |
| *No healthcare/isolation* | 60% (3/5) | 40% (2/5) | † |  |  |  |  |  |  |  |

PAM = Preliminary Adjusted Model, aRR = Minimally Adjusted Risk Ratio, ARR = Adjusted Risk Ratio

Minimally adjusted means only controlling for current food insecurity

Green coloring used to highlight factors with p≤0.05

Violence model was fitted separately because inclusion of the violence variable then restricts the underlying sample to those in a relationship or cohabitating with others (family or friends).

† Minimally adjusted model not fitted due to presence of cells with 0 values

# Supplemental Material 6. All performed analyses to identify T3 predictors of food insecurity at T4

|  | **Food insecure** | | **Minimally Adjusted** | | **PAM 1** | | **PAM 2** | | **Final model** | |
| --- | --- | --- | --- | --- | --- | --- | --- | --- | --- | --- |
| **Characteristic** | **No, N = 135** | **Yes, N = 32** | **aRR [95% CI]** | **p-value** | **ARR [95% CI]** | **p-value** | **ARR [95% CI]** | **p-value** | **ARR [95% CI]** | **p-value** |
| **Age (Continuous)** | 19 (19-20) | 20 (19-21) | 1.19 [0.90-1.57] | 0.211 |  |  |  |  |  |  |
| **Racial identity** |  |  |  |  |  |  |  |  |  |  |
| *White* | 80% (84/105) | 20% (21/105) | 1.00 [ref] |  |  |  |  |  |  |  |
| *Asian/Asian American* | 81% (30/37) | 19% (7/37) | 1.04 [0.53-2.05] | 0.911 |  |  |  |  |  |  |
| *Other/Multiracial* | 85% (17/20) | 15% (3/20) | 0.65 [0.25-1.74] | 0.393 |  |  |  |  |  |  |
| **Hispanic ethnicity** |  |  |  |  |  |  |  |  |  |  |
| *No* | 80% (115/143) | 20% (28/143) | 1.00 [ref] |  |  |  |  |  |  |  |
| *Yes* | 81% (17/21) | 19% (4/21) | 0.67 [0.34-1.32] | 0.245 |  |  |  |  |  |  |
| **School year** |  |  |  |  |  |  |  |  |  |  |
| *First-year* | 88% (45/51) | 12% (6/51) | 1.00 [ref] |  | 1.00 [ref] |  | 1.00 [ref] |  | 1.00 [ref] |  |
| *Sophomore* | 79% (30/38) | 21% (8/38) | 1.82 [0.77-4.30] | 0.175 | 1.56 [0.69-3.50] | 0.285 | 1.59 [0.71-3.57] | 0.258 | 1.56 [0.70-3.46] | 0.275 |
| *Junior* | 83% (44/53) | 17% (9/53) | 1.39 [0.59-3.31] | 0.453 | 1.20 [0.52-2.78] | 0.671 | 1.24 [0.54-2.83] | 0.609 | 1.21 [0.55-2.68] | 0.631 |
| *Senior* | 64% (16/25) | 36% (9/25) | 2.89 [1.21-6.89] | **0.017** | 2.42 [0.99-5.89] | 0.052 | 2.57 [1.10-6.02] | **0.03** | 2.53 [1.11-5.78] | **0.028** |
| **Gender Identity** |  |  |  |  |  |  |  |  |  |  |
| *Cisgender woman* | 83% (129/156) | 17% (27/156) | 1.00 [ref] |  | 1.00 [ref] |  |  |  |  |  |
| *Transgender/Gender non-conforming* | 55% (6/11) | 45% (5/11) | 1.94 [1.06-3.54] | **0.032** | 1.33 [0.65-2.71] | 0.438 |  |  |  |  |
| **Received need-based financial aid** |  |  |  |  |  |  |  |  |  |  |
| *No* | 89% (84/94) | 11% (10/94) | 1.00 [ref] |  | 1.00 [ref] |  | 1.00 [ref] |  | 1.00 [ref] |  |
| *Yes* | 66% (43/65) | 34% (22/65) | 2.23 [1.13-4.39] | **0.021** | 2.06 [1.09-3.86] | **0.025** | 2.13 [1.13-4.01] | **0.019** | 2.21 [1.15-4.22] | **0.017** |
| **Food insecure** |  |  |  |  |  |  |  |  |  |  |
| *No* | 88% (126/143) | 12% (17/143) |  |  | 1.00 [ref] |  | 1.00 [ref] |  | 1.00 [ref] |  |
| *Yes* | 38% (9/24) | 63% (15/24) |  |  | 3.33 [1.91-5.81] | **<0.001** | 3.40 [1.95-5.94] | **<0.001** | 4.12 [2.48-6.84] | **<0.001** |
| **Lives with...** |  |  |  |  |  |  |  |  |  |  |
| *Family* | 86% (76/88) | 14% (12/88) | 1.00 [ref] |  |  |  |  |  |  |  |
| *Friends/Roommate(s)/Significant other* | 74% (50/68) | 26% (18/68) | 1.57 [0.84-2.94] | 0.160 |  |  |  |  |  |  |
| *Alone* | 88% (7/8) | 13% (1/8) | 0.86 [0.23-3.31] | 0.831 |  |  |  |  |  |  |
| **Location** |  |  |  |  |  |  |  |  |  |  |
| *Outside NYC metro area* | 87% (61/70) | 13% (9/70) | 1.00 [ref] |  |  |  |  |  |  |  |
| *Off-campus NYC metro area* | 79% (56/71) | 21% (15/71) | 1.18 [0.56-2.48] | 0.672 |  |  |  |  |  |  |
| *On-campus housing* | 69% (18/26) | 31% (8/26) | 1.43 [0.68-3.02] | 0.344 |  |  |  |  |  |  |
| **Home perceived unsafe** |  |  |  |  |  |  |  |  |  |  |
| *No* | 80% (103/128) | 20% (25/128) | 1.00 [ref] |  |  |  |  |  |  |  |
| *Yes* | 84% (32/38) | 16% (6/38) | 0.69 [0.35-1.40] | 0.305 |  |  |  |  |  |  |
| **Social group involvement** |  |  |  |  |  |  |  |  |  |  |
| *No* | 84% (75/89) | 16% (14/89) | 1.00 [ref] |  |  |  |  |  |  |  |
| *Yes* | 76% (58/76) | 24% (18/76) | 1.04 [0.57-1.88] | 0.905 |  |  |  |  |  |  |
| **Sports involvement** |  |  |  |  |  |  |  |  |  |  |
| *No* | 81% (129/160) | 19% (31/160) | 1.00 [ref] |  |  |  |  |  |  |  |
| *Yes* | 86% (6/7) | 14% (1/7) | 0.74 [0.23-2.37] | 0.611 |  |  |  |  |  |  |
| **Relationship status** |  |  |  |  |  |  |  |  |  |  |
| *Single* | 79% (85/108) | 21% (23/108) | 1.00 [ref] |  |  |  |  |  |  |  |
| *In some form of relationship* | 84% (49/58) | 16% (9/58) | 0.75 [0.39-1.45] | 0.392 |  |  |  |  |  |  |
| **Experienced physical/verbal violence** |  |  |  |  |  |  |  |  |  |  |
| *No* | 81% (87/108) | 19% (21/108) | 1.00 [ref] |  |  |  |  |  |  |  |
| *Yes* | 81% (42/52) | 19% (10/52) | 1.08 [0.60-1.93] | 0.794 |  |  |  |  |  |  |
| **Condom use status** |  |  |  |  |  |  |  |  |  |  |
| *No sexual activity* | 82% (72/88) | 18% (16/88) | 1.00 [ref] |  |  |  |  |  |  |  |
| *No condoms* | 83% (20/24) | 17% (4/24) | 0.85 [0.31-2.32] | 0.753 |  |  |  |  |  |  |
| *Used condoms* | 82% (36/44) | 18% (8/44) | 1.06 [0.51-2.19] | 0.873 |  |  |  |  |  |  |
| **Currently smokes/vapes** |  |  |  |  |  |  |  |  |  |  |
| *No* | 81% (122/150) | 19% (28/150) | 1.00 [ref] |  |  |  |  |  |  |  |
| *Yes* | 71% (10/14) | 29% (4/14) | 1.25 [0.47-3.37] | 0.656 |  |  |  |  |  |  |
| **Frequency of alcohol use** |  |  |  |  |  |  |  |  |  |  |
| *Never/Rare* | 80% (89/111) | 20% (22/111) | 1.00 [ref] |  |  |  |  |  |  |  |
| *Moderate/High* | 82% (46/56) | 18% (10/56) | 1.36 [0.71-2.62] | 0.357 |  |  |  |  |  |  |
| **Frequency of drug use** |  |  |  |  |  |  |  |  |  |  |
| *Never/Rare* | 82% (108/132) | 18% (24/132) | 1.00 [ref] |  |  |  |  |  |  |  |
| *Monthly/Weekly/Daily* | 76% (26/34) | 24% (8/34) | 1.17 [0.59-2.30] | 0.651 |  |  |  |  |  |  |
| **Since 3 months ago, feel...** |  |  |  |  |  |  |  |  |  |  |
| *Less lonely* | 85% (79/93) | 15% (14/93) | 1.00 [ref] |  |  |  |  |  |  |  |
| *Same* | 77% (30/39) | 23% (9/39) | 1.28 [0.65-2.52] | 0.484 |  |  |  |  |  |  |
| *Lonelier* | 74% (26/35) | 26% (9/35) | 1.36 [0.72-2.57] | 0.350 |  |  |  |  |  |  |
| **Had a social support network** |  |  |  |  |  |  |  |  |  |  |
| *Yes* | 86% (115/134) | 14% (19/134) | 1.00 [ref] |  | 1.00 [ref] |  | 1.00 [ref] |  |  |  |
| *Uncertain* | 69% (9/13) | 31% (4/13) | 1.67 [0.61-4.58] | 0.322 | 1.77 [0.68-4.61] | 0.242 | 1.71 [0.66-4.42] | 0.269 |  |  |
| *No* | 50% (9/18) | 50% (9/18) | 2.10 [1.19-3.70] | **0.010** | 1.68 [0.95-2.97] | 0.076 | 1.68 [0.96-2.94] | 0.069 |  |  |
| **Moderate-severe psychological distress** |  |  |  |  |  |  |  |  |  |  |
| *No* | 87% (73/84) | 13% (11/84) | 1.00 [ref] |  |  |  |  |  |  |  |
| *Yes* | 74% (61/82) | 26% (21/82) | 1.53 [0.82-2.86] | 0.183 |  |  |  |  |  |  |
| **Currently using hormones** |  |  |  |  |  |  |  |  |  |  |
| *No* | 78% (73/94) | 22% (21/94) | 1.00 [ref] |  |  |  |  |  |  |  |
| *Yes* | 85% (62/73) | 15% (11/73) | 0.74 [0.41-1.35] | 0.330 |  |  |  |  |  |  |
| **Sought care for COVID-19 symptoms** |  |  |  |  |  |  |  |  |  |  |
| *No symptoms* | 80% (121/151) | 20% (30/151) | † |  |  |  |  |  |  |  |
| *Sought healthcare* | 100% (6/6) | 0% (0/6) | † |  |  |  |  |  |  |  |
| *Self-isolated* | 67% (2/3) | 33% (1/3) | † |  |  |  |  |  |  |  |
| *No healthcare/isolation* | 86% (6/7) | 14% (1/7) | † |  |  |  |  |  |  |  |

PAM = Preliminary Adjusted Model, aRR = Minimally Adjusted Risk Ratio, ARR = Adjusted Risk Ratio

Minimally adjusted means only controlling for current food insecurity

Green coloring used to highlight factors with p≤0.05

† Minimally adjusted model not fitted due to presence of cells with 0 values

# Supplemental Material 7. All performed analyses to identify factors marginally associated with food insecurity in a Generalized Estimating Equations (GEE) model (N=167)

|  | **Unadjusted** | | **PAM 1** | | **PAM 2** | | **PAM 3** | | **Final model** | |
| --- | --- | --- | --- | --- | --- | --- | --- | --- | --- | --- |
| **Characteristic** | **RR [95% CI]** | **p-value** | **ARR [95% CI]** | **p-value** | **ARR [95% CI]** | **p-value** | **ARR [95% CI]** | **p-value** | **ARR [95% CI]** | **p-value** |
| **Age (Continuous)** | 1.05 [0.86-1.29] | 0.606 |  |  |  |  |  |  |  |  |
| **Racial identity** |  |  |  |  |  |  |  |  |  |  |
| *White* | 1.00 [ref] |  |  |  |  |  |  |  |  |  |
| *Asian/Asian American* | 0.80 [0.40-1.59] | 0.519 |  |  |  |  |  |  |  |  |
| *Other/Multiracial* | 1.59 [0.79-3.18] | 0.195 |  |  |  |  |  |  |  |  |
| **Hispanic ethnicity** |  |  |  |  |  |  |  |  |  |  |
| *No* | 1.00 [ref] |  |  |  |  |  |  |  |  |  |
| *Yes* | 1.40 [0.65-3.00] | 0.385 |  |  |  |  |  |  |  |  |
| **School year** |  |  |  |  |  |  |  |  |  |  |
| *First-year* | 1.00 [ref] |  |  |  |  |  |  |  |  |  |
| *Sophomore* | 1.13 [0.52-2.45] | 0.761 |  |  |  |  |  |  |  |  |
| *Junior* | 1.16 [0.57-2.37] | 0.674 |  |  |  |  |  |  |  |  |
| *Senior* | 1.55 [0.73-3.30] | 0.25 |  |  |  |  |  |  |  |  |
| **Gender Identity** |  |  |  |  |  |  |  |  |  |  |
| *Cisgender woman* | 1.00 [ref] |  | 1.00 [ref] |  | 1.00 [ref] |  | 1.00 [ref] |  | 1.00 [ref] |  |
| *Transgender/Gender non-conforming* | 2.36 [1.19-4.70] | **0.014** | 2.00 [1.14-3.50] | **0.016** | 2.00 [1.17-3.40] | **0.011** | 2.06 [1.21-3.49] | **0.008** | 2.04 [1.19-3.48] | **0.009** |
| **Received need-based financial aid** |  |  |  |  |  |  |  |  |  |  |
| *No* | 1.00 [ref] |  | 1.00 [ref] |  | 1.00 [ref] |  | 1.00 [ref] |  | 1.00 [ref] |  |
| *Yes* | 3.99 [2.31-6.87] | **<0.001** | 3.56 [2.04-6.22] | **<0.001** | 3.55 [2.01-6.25] | **<0.001** | 3.36 [1.96-5.76] | **<0.001** | 3.48 [2.02-5.99] | **<0.001** |
| **Lives with...** |  |  |  |  |  |  |  |  |  |  |
| *Alone* | 1.00 [ref] |  | 1.00 [ref] |  | 1.00 [ref] |  |  |  |  |  |
| *Friends/Roommate(s)/Significant other* | 0.63 [0.40-0.99] | **0.045** | 0.84 [0.52-1.36] | 0.482 | 0.85 [0.52-1.37] | 0.498 |  |  |  |  |
| *Family* | 0.43 [0.24-0.77] | **0.004** | 0.63 [0.36-1.12] | 0.114 | 0.63 [0.35-1.12] | 0.116 |  |  |  |  |
| **Location** |  |  |  |  |  |  |  |  |  |  |
| *Outside NYC metro area* | 1.00 [ref] |  |  |  |  |  |  |  |  |  |
| *Off-campus NYC metro area* | 1.13 [0.69-1.86] | 0.629 |  |  |  |  |  |  |  |  |
| *On-campus housing* | 1.37 [0.93-2.02] | 0.112 |  |  |  |  |  |  |  |  |
| **Home perceived unsafe** |  |  |  |  |  |  |  |  |  |  |
| *No* | 1.00 [ref] |  | 1.00 [ref] |  | 1.00 [ref] |  | 1.00 [ref] |  | 1.00 [ref] |  |
| *Yes* | 2.09 [1.50-2.92] | **<0.001** | 1.76 [1.27-2.44] | **<0.001** | 1.77 [1.27-2.46] | **<0.001** | 1.84 [1.31-2.60] | **<0.001** | 1.87 [1.32-2.65] | **<0.001** |
| **Social group involvement** |  |  |  |  |  |  |  |  |  |  |
| *No* | 1.00 [ref] |  |  |  |  |  |  |  |  |  |
| *Yes* | 1.29 [0.85-1.95] | 0.231 |  |  |  |  |  |  |  |  |
| **Sports involvement** |  |  |  |  |  |  |  |  |  |  |
| *No* | 1.00 [ref] |  |  |  |  |  |  |  |  |  |
| *Yes* | 0.98 [0.49-1.96] | 0.963 |  |  |  |  |  |  |  |  |
| **Relationship status** |  |  |  |  |  |  |  |  |  |  |
| *Single* | 1.00 [ref] |  | 1.00 [ref] |  |  |  |  |  |  |  |
| *In some form of relationship* | 1.42 [0.94-2.14] | 0.092 | 1.25 [0.82-1.90] | 0.295 |  |  |  |  |  |  |
| **Experienced physical/verbal violence** |  |  |  |  |  |  |  |  |  |  |
| *No* | 1.00 [ref] |  |  |  |  |  |  |  |  |  |
| *Yes* | 0.99 [0.74-1.32] | 0.94 |  |  |  |  |  |  |  |  |
| **Condom use status** |  |  |  |  |  |  |  |  |  |  |
| *No sexual activity* | 1.00 [ref] |  |  |  |  |  |  |  |  |  |
| *No condoms* | 1.53 [0.92-2.56] | 0.1 |  |  |  |  |  |  |  |  |
| *Used condoms* | 0.99 [0.55-1.79] | 0.984 |  |  |  |  |  |  |  |  |
| **Currently smokes/vapes** |  |  |  |  |  |  |  |  |  |  |
| *No* | 1.00 [ref] |  |  |  |  |  |  |  |  |  |
| *Yes* | 1.09 [0.46-2.57] | 0.843 |  |  |  |  |  |  |  |  |
| **Frequency of alcohol use** |  |  |  |  |  |  |  |  |  |  |
| *Never/Rare* | 1.00 [ref] |  | 1.00 [ref] |  | 1.00 [ref] |  | 1.00 [ref] |  |  |  |
| *Moderate/High* | 0.64 [0.38-1.07] | 0.089 | 0.68 [0.44-1.04] | 0.076 | 0.68 [0.44-1.05] | 0.081 | 0.70 [0.45-1.08] | 0.109 |  |  |
| **Frequency of drug use** |  |  |  |  |  |  |  |  |  |  |
| *Never/Rare* | 1.00 [ref] |  |  |  |  |  |  |  |  |  |
| *Monthly/Weekly/Daily* | 1.05 [0.70-1.58] | 0.824 |  |  |  |  |  |  |  |  |
| **Since 3 months ago, feel...** |  |  |  |  |  |  |  |  |  |  |
| *Less lonely* | 1.00 [ref] |  |  |  |  |  |  |  |  |  |
| *Same* | 1.20 [0.81-1.76] | 0.366 |  |  |  |  |  |  |  |  |
| *Lonelier* | 1.07 [0.75-1.52] | 0.696 |  |  |  |  |  |  |  |  |
| **Had a social support network** |  |  |  |  |  |  |  |  |  |  |
| *Yes* | 1.00 [ref] |  |  |  |  |  |  |  |  |  |
| *Uncertain* | 0.98 [0.62-1.53] | 0.919 |  |  |  |  |  |  |  |  |
| *No* | 1.03 [0.53-1.98] | 0.938 |  |  |  |  |  |  |  |  |
| **Moderate-severe psychological distress** |  |  |  |  |  |  |  |  |  |  |
| *No* | 1.00 [ref] |  |  |  |  |  |  |  |  |  |
| *Yes* | 1.30 [0.89-1.90] | 0.176 |  |  |  |  |  |  |  |  |
| **Currently using hormones** |  |  |  |  |  |  |  |  |  |  |
| *No* | 1.00 [ref] |  |  |  |  |  |  |  |  |  |
| *Yes* | 1.39 [0.89-2.17] | 0.153 |  |  |  |  |  |  |  |  |
| **Sought care for COVID-19 symptoms** |  |  |  |  |  |  |  |  |  |  |
| *No symptoms* | 1.00 [ref] |  |  |  |  |  |  |  |  |  |
| *Sought healthcare* | 0.75 [0.36-1.55] | 0.434 |  |  |  |  |  |  |  |  |
| *Self-isolated* | 1.84 [0.86-3.94] | 0.117 |  |  |  |  |  |  |  |  |
| *No healthcare/isolation* | 0.56 [0.19-1.66] | 0.295 |  |  |  |  |  |  |  |  |

PAM = Preliminary Adjusted Model, ARR = Adjusted Risk Ratio

Green coloring used to highlight factors with p≤0.05

# Supplemental Material 8. All performed analyses to identify correlates of food insecurity at T1

## Supplemental Material 8A. Without violence fitted in model

|  | **Food insecure** | | **Unadjusted** | | | **PAM 1** | | **PAM 2** | | **PAM 3** | | **PAM 4** | | **PAM 5** | | **Final model** | |
| --- | --- | --- | --- | --- | --- | --- | --- | --- | --- | --- | --- | --- | --- | --- | --- | --- | --- |
| **Characteristic** | **No, N = 438** | **Yes, N = 118** | | **PR [95% CI]** | **p-value** | **APR [95% CI]** | **p-value** | **APR [95% CI]** | **p-value** | **APR [95% CI]** | **p-value** | **APR [95% CI]** | **p-value** | **APR [95% CI]** | **p-value** | **APR [95% CI]** | **p-value** |
| **Age (Continuous)** | 20 (19-21) | 20 (19-21) | | 1.07 [0.94-1.21] | 0.295 |  |  |  |  |  |  |  |  |  |  |  |  |
| **Racial identity** |  |  | |  |  |  |  |  |  |  |  |  |  |  |  |  |  |
| *White* | 81% (272/335) | 19% (63/335) | | 1.00 [ref] |  | 1.00 [ref] |  | 1.00 [ref] |  | 1.00 [ref] |  | 1.00 [ref] |  | 1.00 [ref] |  |  |  |
| *Asian/Asian American* | 83% (90/108) | 17% (18/108) | | 0.89 [0.55-1.43] | 0.620 | 0.87 [0.51-1.50] | 0.614 | 0.85 [0.50-1.43] | 0.535 | 0.83 [0.50-1.39] | 0.478 | 0.81 [0.49-1.36] | 0.435 | 0.80 [0.49-1.31] | 0.374 |  |  |
| *Other/Multiracial* | 71% (65/92) | 29% (27/92) | | 1.56 [1.06-2.30] | **0.024** | 1.02 [0.68-1.54] | 0.917 | 1.02 [0.69-1.53] | 0.913 | 1.01 [0.67-1.50] | 0.980 | 1.00 [0.67-1.49] | 0.997 | 1.04 [0.72-1.53] | 0.822 |  |  |
| **Hispanic ethnicity** |  |  | |  |  |  |  |  |  |  |  |  |  |  |  |  |  |
| *No* | 81% (380/470) | 19% (90/470) | | 1.00 [ref] |  | 1.00 [ref] |  |  |  |  |  |  |  |  |  |  |  |
| *Yes* | 69% (53/77) | 31% (24/77) | | 1.63 [1.11-2.38] | **0.012** | 1.07 [0.67-1.71] | 0.769 |  |  |  |  |  |  |  |  |  |  |
| **School year** |  |  | |  |  |  |  |  |  |  |  |  |  |  |  |  |  |
| *First-year* | 80% (116/145) | 20% (29/145) | | 1.00 [ref] |  |  |  |  |  |  |  |  |  |  |  |  |  |
| *Sophomore* | 83% (101/122) | 17% (21/122) | | 0.86 [0.52-1.43] | 0.562 |  |  |  |  |  |  |  |  |  |  |  |  |
| *Junior* | 79% (120/152) | 21% (32/152) | | 1.05 [0.67-1.65] | 0.822 |  |  |  |  |  |  |  |  |  |  |  |  |
| *Senior* | 74% (101/136) | 26% (35/136) | | 1.29 [0.83-1.98] | 0.254 |  |  |  |  |  |  |  |  |  |  |  |  |
| **Gender Identity** |  |  | |  |  |  |  |  |  |  |  |  |  |  |  |  |  |
| *Cisgender woman* | 80% (428/537) | 20% (109/537) | | 1.00 [ref] |  | 1.00 [ref] |  | 1.00 [ref] |  | 1.00 [ref] |  | 1.00 [ref] |  | 1.00 [ref] |  | 1.00 [ref] |  |
| *Transgender/Gender non-conforming* | 53% (10/19) | 47% (9/19) | | 2.33 [1.41-3.86] | **<0.001** | 1.51 [0.85-2.71] | 0.162 | 1.47 [0.84-2.56] | 0.180 | 1.45 [0.84-2.49] | 0.182 | 1.49 [0.88-2.53] | 0.142 | 1.50 [0.90-2.52] | 0.121 | 1.72 [1.10-2.67] | **0.017** |
| **Received need-based financial aid** |  |  | |  |  |  |  |  |  |  |  |  |  |  |  |  |  |
| *No* | 90% (273/302) | 10% (29/302) | | 1.00 [ref] |  | 1.00 [ref] |  | 1.00 [ref] |  | 1.00 [ref] |  | 1.00 [ref] |  | 1.00 [ref] |  | 1.00 [ref] |  |
| *Yes* | 65% (155/237) | 35% (82/237) | | 3.60 [2.44-5.31] | **<0.001** | 3.39 [2.27-5.05] | **<0.001** | 3.45 [2.31-5.16] | **<0.001** | 3.51 [2.35-5.25] | **<0.001** | 3.51 [2.35-5.25] | **<0.001** | 3.56 [2.39-5.31] | **<0.001** | 3.93 [2.67-5.77] | **<0.001** |
| **Lives with...** |  |  | |  |  |  |  |  |  |  |  |  |  |  |  |  |  |
| *Family* | 81% (242/300) | 19% (58/300) | | 1.00 [ref] |  |  |  |  |  |  |  |  |  |  |  |  |  |
| *Friends/Roommate(s)/Significant other* | 77% (172/222) | 23% (50/222) | | 1.16 [0.83-1.63] | 0.373 |  |  |  |  |  |  |  |  |  |  |  |  |
| *Alone* | 73% (22/30) | 27% (8/30) | | 1.38 [0.73-2.61] | 0.322 |  |  |  |  |  |  |  |  |  |  |  |  |
| **Location** |  |  | |  |  |  |  |  |  |  |  |  |  |  |  |  |  |
| *Outside NYC metro area* | 85% (213/251) | 15% (38/251) | | 1.00 [ref] |  | 1.00 [ref] |  | 1.00 [ref] |  | 1.00 [ref] |  | 1.00 [ref] |  | 1.00 [ref] |  | 1.00 [ref] |  |
| *Off-campus NYC metro area* | 75% (217/290) | 25% (73/290) | | 1.66 [1.17-2.37] | **0.005** | 1.69 [1.15-2.48] | **0.008** | 1.67 [1.14-2.44] | **0.008** | 1.67 [1.15-2.43] | **0.007** | 1.67 [1.15-2.43] | **0.007** | 1.63 [1.13-2.36] | **0.009** | 1.65 [1.17-2.32] | **0.004** |
| *On-campus housing* | 57% (8/14) | 43% (6/14) | | 2.83 [1.45-5.54] | **0.002** | 1.93 [0.67-5.55] | 0.221 | 2.26 [0.96-5.35] | 0.062 | 2.65 [1.27-5.55] | **0.01** | 2.62 [1.26-5.45] | **0.01** | 2.66 [1.36-5.21] | **0.004** | 2.22 [1.10-4.45] | **0.025** |
| **Home perceived unsafe** |  |  | |  |  |  |  |  |  |  |  |  |  |  |  |  |  |
| *No* | 83% (347/418) | 17% (71/418) | | 1.00 [ref] |  | 1.00 [ref] |  | 1.00 [ref] |  | 1.00 [ref] |  | 1.00 [ref] |  | 1.00 [ref] |  | 1.00 [ref] |  |
| *Yes* | 67% (88/131) | 33% (43/131) | | 1.93 [1.40-2.67] | **<0.001** | 1.57 [1.08-2.27] | **0.017** | 1.57 [1.09-2.25] | **0.015** | 1.51 [1.07-2.14] | **0.018** | 1.52 [1.07-2.14] | **0.018** | 1.52 [1.08-2.15] | **0.017** | 1.43 [1.04-1.98] | **0.027** |
| **Social group involvement** |  |  | |  |  |  |  |  |  |  |  |  |  |  |  |  |  |
| *No* | 80% (173/216) | 20% (43/216) | | 1.00 [ref] |  |  |  |  |  |  |  |  |  |  |  |  |  |
| *Yes* | 78% (264/339) | 22% (75/339) | | 1.11 [0.80-1.55] | 0.535 |  |  |  |  |  |  |  |  |  |  |  |  |
| **Sports involvement** |  |  | |  |  |  |  |  |  |  |  |  |  |  |  |  |  |
| *No* | 78% (376/483) | 22% (107/483) | | 1.00 [ref] |  |  |  |  |  |  |  |  |  |  |  |  |  |
| *Yes* | 85% (62/73) | 15% (11/73) | | 0.68 [0.38-1.20] | 0.185 |  |  |  |  |  |  |  |  |  |  |  |  |
| **Relationship status** |  |  | |  |  |  |  |  |  |  |  |  |  |  |  |  |  |
| *Single* | 82% (318/387) | 18% (69/387) | | 1.00 [ref] |  | 1.00 [ref] |  | 1.00 [ref] |  | 1.00 [ref] |  | 1.00 [ref] |  | 1.00 [ref] |  | 1.00 [ref] |  |
| *In some form of relationship* | 72% (118/165) | 28% (47/165) | | 1.60 [1.16-2.21] | **0.004** | 1.84 [1.19-2.84] | **0.006** | 1.77 [1.15-2.73] | **0.009** | 1.81 [1.18-2.79] | **0.007** | 1.78 [1.17-2.71] | **0.007** | 1.78 [1.28-2.48] | **<0.001** | 1.75 [1.28-2.39] | **<0.001** |
| **Experienced physical/verbal violence** |  |  | |  |  |  |  |  |  |  |  |  |  |  |  |  |  |
| *No* | 83% (215/258) | 17% (43/258) | | 1.00 [ref] |  |  |  |  |  |  |  |  |  |  |  |  |  |
| *Yes* | 75% (204/273) | 25% (69/273) | | 1.52 [1.08-2.13] | **0.017** |  |  |  |  |  |  |  |  |  |  |  |  |
| **Condom use status** |  |  | |  |  |  |  |  |  |  |  |  |  |  |  |  |  |
| *No sexual activity* | 82% (286/347) | 18% (61/347) | | 1.00 [ref] |  | 1.00 [ref] |  | 1.00 [ref] |  | 1.00 [ref] |  | 1.00 [ref] |  |  |  |  |  |
| *No condoms* | 67% (56/84) | 33% (28/84) | | 1.90 [1.30-2.77] | **<0.001** | 1.13 [0.64-2.00] | 0.672 | 1.19 [0.68-2.09] | 0.545 | 1.16 [0.67-2.03] | 0.593 | 1.19 [0.70-2.04] | 0.523 |  |  |  |  |
| *Used condoms* | 77% (80/104) | 23% (24/104) | | 1.31 [0.86-1.99] | 0.202 | 0.85 [0.51-1.44] | 0.549 | 0.85 [0.50-1.43] | 0.529 | 0.81 [0.48-1.37] | 0.437 | 0.83 [0.50-1.38] | 0.470 |  |  |  |  |
| **Currently smokes/vapes** |  |  | |  |  |  |  |  |  |  |  |  |  |  |  |  |  |
| *No* | 80% (412/513) | 20% (101/513) | | 1.00 [ref] |  | 1.00 [ref] |  | 1.00 [ref] |  | 1.00 [ref] |  | 1.00 [ref] |  | 1.00 [ref] |  | 1.00 [ref] |  |
| *Yes* | 60% (25/42) | 40% (17/42) | | 2.06 [1.37-3.09] | **<0.001** | 1.62 [0.92-2.86] | 0.093 | 1.73 [1.01-2.97] | **0.047** | 1.76 [1.04-2.98] | **0.036** | 1.83 [1.12-3.00] | **0.016** | 1.85 [1.19-2.88] | **0.007** | 1.84 [1.22-2.78] | **0.004** |
| **Frequency of alcohol use** |  |  | |  |  |  |  |  |  |  |  |  |  |  |  |  |  |
| *Never/Rare* | 77% (295/381) | 23% (86/381) | | 1.00 [ref] |  |  |  |  |  |  |  |  |  |  |  |  |  |
| *Moderate/High* | 82% (143/175) | 18% (32/175) | | 0.81 [0.56-1.17] | 0.257 |  |  |  |  |  |  |  |  |  |  |  |  |
|  |  |  | |  |  |  |  |  |  |  |  |  |  |  |  |  |  |
|  |  |  | |  |  |  |  |  |  |  |  |  |  |  |  |  |  |
| **Frequency of drug use** |  |  | |  |  |  |  |  |  |  |  |  |  |  |  |  |  |
| *Never/Rare* | 81% (352/437) | 19% (85/437) | | 1.00 [ref] |  | 1.00 [ref] |  | 1.00 [ref] |  | 1.00 [ref] |  |  |  |  |  |  |  |
| *Monthly/Weekly/Daily* | 72% (86/119) | 28% (33/119) | | 1.43 [1.01-2.02] | **0.045** | 1.12 [0.72-1.74] | 0.626 | 1.13 [0.73-1.75] | 0.594 | 1.11 [0.72-1.71] | 0.646 |  |  |  |  |  |  |
| **Since 3 months ago, feel...** |  |  | |  |  |  |  |  |  |  |  |  |  |  |  |  |  |
| *Less lonely* | 81% (176/216) | 19% (40/216) | | 1.00 [ref] |  |  |  |  |  |  |  |  |  |  |  |  |  |
| *Same* | 77% (85/111) | 23% (26/111) | | 1.26 [0.82-1.96] | 0.292 |  |  |  |  |  |  |  |  |  |  |  |  |
| *Lonelier* | 77% (175/227) | 23% (52/227) | | 1.24 [0.86-1.79] | 0.257 |  |  |  |  |  |  |  |  |  |  |  |  |
| **Had a social support network** |  |  | |  |  |  |  |  |  |  |  |  |  |  |  |  |  |
| *Yes* | 81% (330/406) | 19% (76/406) | | 1.00 [ref] |  | 1.00 [ref] |  | 1.00 [ref] |  |  |  |  |  |  |  |  |  |
| *Uncertain* | 72% (50/69) | 28% (19/69) | | 1.47 [0.95-2.27] | 0.081 | 1.14 [0.66-1.96] | 0.644 | 1.11 [0.64-1.91] | 0.710 |  |  |  |  |  |  |  |  |
| *No* | 70% (52/74) | 30% (22/74) | | 1.59 [1.06-2.38] | **0.025** | 0.93 [0.57-1.51] | 0.774 | 0.93 [0.58-1.49] | 0.752 |  |  |  |  |  |  |  |  |
| **Moderate-severe psychological distress** |  |  | |  |  |  |  |  |  |  |  |  |  |  |  |  |  |
| *No* | 87% (126/145) | 13% (19/145) | | 1.00 [ref] |  | 1.00 [ref] |  | 1.00 [ref] |  | 1.00 [ref] |  | 1.00 [ref] |  | 1.00 [ref] |  | 1.00 [ref] |  |
| *Yes* | 76% (310/407) | 24% (97/407) | | 1.82 [1.16-2.86] | **0.010** | 1.62 [0.99-2.65] | 0.055 | 1.65 [1.01-2.70] | **0.046** | 1.65 [1.01-2.69] | **0.047** | 1.65 [1.01-2.70] | **0.044** | 1.71 [1.05-2.79] | **0.031** | 1.66 [1.04-2.66] | **0.035** |
| **Currently using hormones** |  |  | |  |  |  |  |  |  |  |  |  |  |  |  |  |  |
| *No* | 78% (283/362) | 22% (79/362) | | 1.00 [ref] |  |  |  |  |  |  |  |  |  |  |  |  |  |
| *Yes* | 80% (154/193) | 20% (39/193) | | 0.93 [0.66-1.30] | 0.659 |  |  |  |  |  |  |  |  |  |  |  |  |
| **Sought care for COVID-19 symptoms** |  |  | |  |  |  |  |  |  |  |  |  |  |  |  |  |  |
| *No symptoms* | 80% (347/435) | 20% (88/435) | | 1.00 [ref] |  |  |  |  |  |  |  |  |  |  |  |  |  |
| *Sought healthcare* | 80% (49/61) | 20% (12/61) | | 0.97 [0.57-1.67] | 0.919 |  |  |  |  |  |  |  |  |  |  |  |  |
| *Self-isolated* | 70% (28/40) | 30% (12/40) | | 1.48 [0.89-2.47] | 0.129 |  |  |  |  |  |  |  |  |  |  |  |  |
| *No healthcare/isolation* | 74% (14/19) | 26% (5/19) | | 1.30 [0.60-2.82] | 0.506 |  |  |  |  |  |  |  |  |  |  |  |  |

PAM = Preliminary Adjusted Model, PR = Prevalence Ratio, APR = Adjusted Prevalence Ratio

Green coloring used to highlight factors with p≤0.05

## Supplemental Material 8B. With violence fitted in model

|  | **Food insecure** | | **Unadjusted** | | | **PAM 1** | | **PAM 2** | | **PAM 3** | | **PAM 4** | | **PAM 5** | | **PAM 6** | | **Final model** | |
| --- | --- | --- | --- | --- | --- | --- | --- | --- | --- | --- | --- | --- | --- | --- | --- | --- | --- | --- | --- |
| **Characteristic** | **No, N = 438** | **Yes, N = 118** | | **PR [95% CI]** | **p-value** | **APR [95% CI]** | **p-value** | **APR [95% CI]** | **p-value** | **APR [95% CI]** | **p-value** | **APR [95% CI]** | **p-value** | **APR [95% CI]** | **p-value** | **APR [95% CI]** | **p-value** | **APR [95% CI]** | **p-value** |
| **Age (Continuous)** | 20 (19-21) | 20 (19-21) | | 1.07 [0.94-1.21] | 0.295 |  |  |  |  |  |  |  |  |  |  |  |  |  |  |
| **Racial identity** |  |  | |  |  |  |  |  |  |  |  |  |  |  |  |  |  |  |  |
| *White* | 81% (272/335) | 19% (63/335) | | 1.00 [ref] |  | 1.00 [ref] |  | 1.00 [ref] |  | 1.00 [ref] |  | 1.00 [ref] |  | 1.00 [ref] |  |  |  |  |  |
| *Asian/Asian American* | 83% (90/108) | 17% (18/108) | | 0.89 [0.55-1.43] | 0.620 | 0.82 [0.48-1.41] | 0.477 | 0.81 [0.48-1.37] | 0.426 | 0.79 [0.47-1.33] | 0.375 | 0.77 [0.45-1.31] | 0.333 | 0.75 [0.45-1.25] | 0.268 |  |  |  |  |
| *Other/Multiracial* | 71% (65/92) | 29% (27/92) | | 1.56 [1.06-2.30] | **0.024** | 0.90 [0.59-1.37] | 0.617 | 0.90 [0.60-1.36] | 0.615 | 0.90 [0.59-1.35] | 0.606 | 0.89 [0.59-1.34] | 0.568 | 0.94 [0.64-1.40] | 0.773 |  |  |  |  |
| **Hispanic ethnicity** |  |  | |  |  |  |  |  |  |  |  |  |  |  |  |  |  |  |  |
| *No* | 81% (380/470) | 19% (90/470) | | 1.00 [ref] |  | 1.00 [ref] |  |  |  |  |  |  |  |  |  |  |  |  |  |
| *Yes* | 69% (53/77) | 31% (24/77) | | 1.63 [1.11-2.38] | **0.012** | 0.98 [0.60-1.60] | 0.930 |  |  |  |  |  |  |  |  |  |  |  |  |
| **School year** |  |  | |  |  |  |  |  |  |  |  |  |  |  |  |  |  |  |  |
| *First-year* | 80% (116/145) | 20% (29/145) | | 1.00 [ref] |  |  |  |  |  |  |  |  |  |  |  |  |  |  |  |
| *Sophomore* | 83% (101/122) | 17% (21/122) | | 0.86 [0.52-1.43] | 0.562 |  |  |  |  |  |  |  |  |  |  |  |  |  |  |
| *Junior* | 79% (120/152) | 21% (32/152) | | 1.05 [0.67-1.65] | 0.822 |  |  |  |  |  |  |  |  |  |  |  |  |  |  |
| *Senior* | 74% (101/136) | 26% (35/136) | | 1.29 [0.83-1.98] | 0.254 |  |  |  |  |  |  |  |  |  |  |  |  |  |  |
| **Gender Identity** |  |  | |  |  |  |  |  |  |  |  |  |  |  |  |  |  |  |  |
| *Cisgender woman* | 80% (428/537) | 20% (109/537) | | 1.00 [ref] |  | 1.00 [ref] |  | 1.00 [ref] |  | 1.00 [ref] |  | 1.00 [ref] |  | 1.00 [ref] |  | 1.00 [ref] |  | 1.00 [ref] |  |
| *Transgender/Gender non-conforming* | 53% (10/19) | 47% (9/19) | | 2.33 [1.41-3.86] | **<0.001** | 1.55 [0.88-2.72] | 0.131 | 1.54 [0.89-2.64] | 0.122 | 1.59 [0.94-2.71] | 0.086 | 1.56 [0.93-2.61] | 0.094 | 1.55 [0.93-2.59] | 0.089 | 1.73 [1.12-2.68] | **0.013** | 1.69 [1.07-2.67] | **0.025** |
| **Received need-based financial aid** |  |  | |  |  |  |  |  |  |  |  |  |  |  |  |  |  |  |  |
| *No* | 90% (273/302) | 10% (29/302) | | 1.00 [ref] |  | 1.00 [ref] |  | 1.00 [ref] |  | 1.00 [ref] |  | 1.00 [ref] |  | 1.00 [ref] |  | 1.00 [ref] |  | 1.00 [ref] |  |
| *Yes* | 65% (155/237) | 35% (82/237) | | 3.60 [2.44-5.31] | **<0.001** | 3.20 [2.15-4.76] | **<0.001** | 3.21 [2.15-4.80] | **<0.001** | 3.21 [2.15-4.79] | **<0.001** | 3.30 [2.21-4.93] | **<0.001** | 3.36 [2.25-5.02] | **<0.001** | 3.64 [2.48-5.36] | **<0.001** | 3.70 [2.56-5.37] | **<0.001** |
| **Lives with...** |  |  | |  |  |  |  |  |  |  |  |  |  |  |  |  |  |  |  |
| *Family* | 81% (242/300) | 19% (58/300) | | 1.00 [ref] |  |  |  |  |  |  |  |  |  |  |  |  |  |  |  |
| *Friends/Roommate(s)/Significant other* | 77% (172/222) | 23% (50/222) | | 1.16 [0.83-1.63] | 0.373 |  |  |  |  |  |  |  |  |  |  |  |  |  |  |
| *Alone* | 73% (22/30) | 27% (8/30) | | 1.38 [0.73-2.61] | 0.322 |  |  |  |  |  |  |  |  |  |  |  |  |  |  |
| **Location** |  |  | |  |  |  |  |  |  |  |  |  |  |  |  |  |  |  |  |
| *Outside NYC metro area* | 85% (213/251) | 15% (38/251) | | 1.00 [ref] |  | 1.00 [ref] |  | 1.00 [ref] |  | 1.00 [ref] |  | 1.00 [ref] |  | 1.00 [ref] |  | 1.00 [ref] |  | 1.00 [ref] |  |
| *Off-campus NYC metro area* | 75% (217/290) | 25% (73/290) | | 1.66 [1.17-2.37] | **0.005** | 1.80 [1.23-2.65] | **0.003** | 1.81 [1.24-2.64] | **0.002** | 1.80 [1.24-2.63] | **0.002** | 1.79 [1.23-2.60] | **0.002** | 1.73 [1.20-2.49] | **0.003** | 1.73 [1.23-2.43] | **0.002** | 1.81 [1.29-2.55] | **<0.001** |
| *On-campus housing* | 57% (8/14) | 43% (6/14) | | 2.83 [1.45-5.54] | **0.002** | 1.51 [0.40-5.66] | 0.542 | 1.98 [0.72-5.47] | 0.186 | 1.96 [0.71-5.39] | 0.191 | 2.49 [1.05-5.91] | **0.038** | 2.68 [1.24-5.79] | **0.012** | 2.33 [1.08-5.03] | **0.031** | 2.51 [1.18-5.35] | **0.017** |
| **Home perceived unsafe** |  |  | |  |  |  |  |  |  |  |  |  |  |  |  |  |  |  |  |
| *No* | 83% (347/418) | 17% (71/418) | | 1.00 [ref] |  | 1.00 [ref] |  | 1.00 [ref] |  | 1.00 [ref] |  | 1.00 [ref] |  | 1.00 [ref] |  | 1.00 [ref] |  |  |  |
| *Yes* | 67% (88/131) | 33% (43/131) | | 1.93 [1.40-2.67] | **<0.001** | 1.50 [1.02-2.21] | **0.037** | 1.48 [1.01-2.16] | **0.042** | 1.48 [1.02-2.16] | **0.042** | 1.42 [0.98-2.05] | 0.065 | 1.43 [0.98-2.06] | 0.061 | 1.36 [0.96-1.93] | 0.082 |  |  |
| **Social group involvement** |  |  | |  |  |  |  |  |  |  |  |  |  |  |  |  |  |  |  |
| *No* | 80% (173/216) | 20% (43/216) | | 1.00 [ref] |  |  |  |  |  |  |  |  |  |  |  |  |  |  |  |
| *Yes* | 78% (264/339) | 22% (75/339) | | 1.11 [0.80-1.55] | 0.535 |  |  |  |  |  |  |  |  |  |  |  |  |  |  |
| **Sports involvement** |  |  | |  |  |  |  |  |  |  |  |  |  |  |  |  |  |  |  |
| *No* | 78% (376/483) | 22% (107/483) | | 1.00 [ref] |  |  |  |  |  |  |  |  |  |  |  |  |  |  |  |
| *Yes* | 85% (62/73) | 15% (11/73) | | 0.68 [0.38-1.20] | 0.185 |  |  |  |  |  |  |  |  |  |  |  |  |  |  |
| **Relationship status** |  |  | |  |  |  |  |  |  |  |  |  |  |  |  |  |  |  |  |
| *Single* | 82% (318/387) | 18% (69/387) | | 1.00 [ref] |  | 1.00 [ref] |  | 1.00 [ref] |  | 1.00 [ref] |  | 1.00 [ref] |  | 1.00 [ref] |  | 1.00 [ref] |  | 1.00 [ref] |  |
| *In some form of relationship* | 72% (118/165) | 28% (47/165) | | 1.60 [1.16-2.21] | **0.004** | 1.80 [1.16-2.80] | **0.009** | 1.75 [1.13-2.70] | **0.012** | 1.71 [1.11-2.62] | **0.014** | 1.77 [1.16-2.70] | **0.009** | 1.75 [1.26-2.44] | **<0.001** | 1.71 [1.25-2.33] | **<0.001** | 1.76 [1.30-2.39] | **<0.001** |
| **Experienced physical/verbal violence** |  |  | |  |  |  |  |  |  |  |  |  |  |  |  |  |  |  |  |
| *No* | 83% (215/258) | 17% (43/258) | | 1.00 [ref] |  | 1.00 [ref] |  | 1.00 [ref] |  | 1.00 [ref] |  | 1.00 [ref] |  | 1.00 [ref] |  | 1.00 [ref] |  | 1.00 [ref] |  |
| *Yes* | 75% (204/273) | 25% (69/273) | | 1.52 [1.08-2.13] | **0.017** | 1.62 [1.07-2.44] | **0.023** | 1.65 [1.10-2.47] | **0.016** | 1.64 [1.09-2.46] | **0.017** | 1.59 [1.07-2.37] | **0.023** | 1.51 [1.01-2.24] | **0.042** | 1.42 [0.99-2.04] | 0.054 | 1.55 [1.12-2.16] | **0.009** |
| **Condom use status** |  |  | |  |  |  |  |  |  |  |  |  |  |  |  |  |  |  |  |
| *No sexual activity* | 82% (286/347) | 18% (61/347) | | 1.00 [ref] |  | 1.00 [ref] |  | 1.00 [ref] |  | 1.00 [ref] |  | 1.00 [ref] |  |  |  |  |  |  |  |
| *No condoms* | 67% (56/84) | 33% (28/84) | | 1.90 [1.30-2.77] | **<0.001** | 1.15 [0.66-2.02] | 0.625 | 1.21 [0.70-2.09] | 0.503 | 1.24 [0.73-2.12] | 0.425 | 1.20 [0.70-2.04] | 0.505 |  |  |  |  |  |  |
| *Used condoms* | 77% (80/104) | 23% (24/104) | | 1.31 [0.86-1.99] | 0.202 | 0.84 [0.49-1.43] | 0.518 | 0.84 [0.49-1.43] | 0.513 | 0.86 [0.51-1.45] | 0.567 | 0.81 [0.48-1.37] | 0.440 |  |  |  |  |  |  |
| **Currently smokes/vapes** |  |  | |  |  |  |  |  |  |  |  |  |  |  |  |  |  |  |  |
| *No* | 80% (412/513) | 20% (101/513) | | 1.00 [ref] |  | 1.00 [ref] |  | 1.00 [ref] |  | 1.00 [ref] |  | 1.00 [ref] |  | 1.00 [ref] |  | 1.00 [ref] |  | 1.00 [ref] |  |
| *Yes* | 60% (25/42) | 40% (17/42) | | 2.06 [1.37-3.09] | **<0.001** | 1.72 [0.94-3.13] | 0.076 | 1.81 [1.03-3.20] | **0.04** | 1.91 [1.12-3.27] | **0.017** | 1.91 [1.14-3.22] | **0.015** | 1.94 [1.21-3.11] | **0.006** | 1.88 [1.22-2.91] | **0.004** | 1.92 [1.25-2.95] | **0.003** |
| **Frequency of alcohol use** |  |  | |  |  |  |  |  |  |  |  |  |  |  |  |  |  |  |  |
| *Never/Rare* | 77% (295/381) | 23% (86/381) | | 1.00 [ref] |  |  |  |  |  |  |  |  |  |  |  |  |  |  |  |
| *Moderate/High* | 82% (143/175) | 18% (32/175) | | 0.81 [0.56-1.17] | 0.257 |  |  |  |  |  |  |  |  |  |  |  |  |  |  |
| **Frequency of drug use** |  |  | |  |  |  |  |  |  |  |  |  |  |  |  |  |  |  |  |
| *Never/Rare* | 81% (352/437) | 19% (85/437) | | 1.00 [ref] |  | 1.00 [ref] |  | 1.00 [ref] |  |  |  |  |  |  |  |  |  |  |  |
| *Monthly/Weekly/Daily* | 72% (86/119) | 28% (33/119) | | 1.43 [1.01-2.02] | **0.045** | 1.14 [0.73-1.78] | 0.562 | 1.15 [0.74-1.78] | 0.540 |  |  |  |  |  |  |  |  |  |  |
| **Since 3 months ago, feel...** |  |  | |  |  |  |  |  |  |  |  |  |  |  |  |  |  |  |  |
| *Less lonely* | 81% (176/216) | 19% (40/216) | | 1.00 [ref] |  |  |  |  |  |  |  |  |  |  |  |  |  |  |  |
| *Same* | 77% (85/111) | 23% (26/111) | | 1.26 [0.82-1.96] | 0.292 |  |  |  |  |  |  |  |  |  |  |  |  |  |  |
| *Lonelier* | 77% (175/227) | 23% (52/227) | | 1.24 [0.86-1.79] | 0.257 |  |  |  |  |  |  |  |  |  |  |  |  |  |  |
|  |  |  | |  |  |  |  |  |  |  |  |  |  |  |  |  |  |  |  |
|  |  |  | |  |  |  |  |  |  |  |  |  |  |  |  |  |  |  |  |
|  |  |  | |  |  |  |  |  |  |  |  |  |  |  |  |  |  |  |  |
| **Had a social support network** |  |  | |  |  |  |  |  |  |  |  |  |  |  |  |  |  |  |  |
| *Yes* | 81% (330/406) | 19% (76/406) | | 1.00 [ref] |  | 1.00 [ref] |  | 1.00 [ref] |  | 1.00 [ref] |  |  |  |  |  |  |  |  |  |
| *Uncertain* | 72% (50/69) | 28% (19/69) | | 1.47 [0.95-2.27] | 0.081 | 1.22 [0.71-2.09] | 0.471 | 1.19 [0.69-2.03] | 0.536 | 1.16 [0.68-1.98] | 0.577 |  |  |  |  |  |  |  |  |
| *No* | 70% (52/74) | 30% (22/74) | | 1.59 [1.06-2.38] | **0.025** | 0.91 [0.57-1.47] | 0.712 | 0.91 [0.57-1.45] | 0.699 | 0.91 [0.57-1.45] | 0.686 |  |  |  |  |  |  |  |  |
| **Moderate-severe psychological distress** |  |  | |  |  |  |  |  |  |  |  |  |  |  |  |  |  |  |  |
| *No* | 87% (126/145) | 13% (19/145) | | 1.00 [ref] |  | 1.00 [ref] |  | 1.00 [ref] |  | 1.00 [ref] |  | 1.00 [ref] |  | 1.00 [ref] |  | 1.00 [ref] |  | 1.00 [ref] |  |
| *Yes* | 76% (310/407) | 24% (97/407) | | 1.82 [1.16-2.86] | **0.010** | 1.50 [0.90-2.50] | 0.123 | 1.54 [0.92-2.57] | 0.102 | 1.55 [0.92-2.59] | 0.097 | 1.55 [0.93-2.59] | 0.096 | 1.64 [0.98-2.74] | 0.060 | 1.63 [1.00-2.66] | 0.052 | 1.66 [1.03-2.66] | **0.036** |
| **Currently using hormones** |  |  | |  |  |  |  |  |  |  |  |  |  |  |  |  |  |  |  |
| *No* | 78% (283/362) | 22% (79/362) | | 1.00 [ref] |  |  |  |  |  |  |  |  |  |  |  |  |  |  |  |
| *Yes* | 80% (154/193) | 20% (39/193) | | 0.93 [0.66-1.30] | 0.659 |  |  |  |  |  |  |  |  |  |  |  |  |  |  |
| **Sought care for COVID-19 symptoms** |  |  | |  |  |  |  |  |  |  |  |  |  |  |  |  |  |  |  |
| *No symptoms* | 80% (347/435) | 20% (88/435) | | 1.00 [ref] |  |  |  |  |  |  |  |  |  |  |  |  |  |  |  |
| *Sought healthcare* | 80% (49/61) | 20% (12/61) | | 0.97 [0.57-1.67] | 0.919 |  |  |  |  |  |  |  |  |  |  |  |  |  |  |
| *Self-isolated* | 70% (28/40) | 30% (12/40) | | 1.48 [0.89-2.47] | 0.129 |  |  |  |  |  |  |  |  |  |  |  |  |  |  |
| *No healthcare/isolation* | 74% (14/19) | 26% (5/19) | | 1.30 [0.60-2.82] | 0.506 | ` |  |  |  |  |  |  |  |  |  |  |  |  |  |

PAM = Preliminary Adjusted Model, PR = Prevalence Ratio, APR = Adjusted Prevalence Ratio

Violence models were fitted separately because inclusion of the violence variable then restricts the underlying sample to those in a relationship or cohabitating with others (family or friends).

Green coloring used to highlight factors with p≤0.05

# Supplemental Material 9. All performed analyses to identify correlates of food insecurity at T2

|  | **Food insecure** | | **Unadjusted** | | **PAM 1** | | **PAM 2** | | **PAM 3** | | **PAM 4** | | **PAM 5** | | **PAM 6** | | **Final model** | |
| --- | --- | --- | --- | --- | --- | --- | --- | --- | --- | --- | --- | --- | --- | --- | --- | --- | --- | --- |
| **Characteristic** | **No, N = 252** | **Yes, N = 82** | **PR [95% CI]** | **p-value** | **APR [95% CI]** | **p-value** | **APR [95% CI]** | **p-value** | **APR [95% CI]** | **p-value** | **APR [95% CI]** | **p-value** | **APR [95% CI]** | **p-value** | **APR [95% CI]** | **p-value** | **APR [95% CI]** | **p-value** |
| **Age (Continuous)** | 20 (19-21) | 20 (19-21) | 1.13 [0.97-1.30] | 0.109 |  |  |  |  |  |  |  |  |  |  |  |  |  |  |
| **Racial identity** |  |  |  |  |  |  |  |  |  |  |  |  |  |  |  |  |  |  |
| *White* | 78% (158/203) | 22% (45/203) | 1.00 [ref] |  | 1.00 [ref] |  | 1.00 [ref] |  | 1.00 [ref] |  | 1.00 [ref] |  |  |  |  |  |  |  |
| *Asian/Asian American* | 74% (46/62) | 26% (16/62) | 1.16 [0.71-1.91] | 0.547 | 1.38 [0.76-2.50] | 0.284 | 1.33 [0.75-2.39] | 0.331 | 1.38 [0.79-2.44] | 0.260 | 1.36 [0.79-2.35] | 0.267 |  |  |  |  |  |  |
| *Other/Multiracial* | 67% (39/58) | 33% (19/58) | 1.48 [0.94-2.32] | 0.089 | 1.67 [1.01-2.77] | **0.046** | 1.54 [0.95-2.49] | 0.077 | 1.57 [0.98-2.52] | 0.061 | 1.44 [0.90-2.30] | 0.127 |  |  |  |  |  |  |
| **Hispanic ethnicity** |  |  |  |  |  |  |  |  |  |  |  |  |  |  |  |  |  |  |
| *No* | 76% (215/284) | 24% (69/284) | 1.00 [ref] |  |  |  |  |  |  |  |  |  |  |  |  |  |  |  |
| *Yes* | 74% (32/43) | 26% (11/43) | 1.05 [0.61-1.82] | 0.854 |  |  |  |  |  |  |  |  |  |  |  |  |  |  |
| **School year** |  |  |  |  |  |  |  |  |  |  |  |  |  |  |  |  |  |  |
| *First-year* | 79% (74/94) | 21% (20/94) | 1.00 [ref] |  |  |  |  |  |  |  |  |  |  |  |  |  |  |  |
| *Sophomore* | 78% (52/67) | 22% (15/67) | 1.05 [0.58-1.90] | 0.866 |  |  |  |  |  |  |  |  |  |  |  |  |  |  |
| *Junior* | 75% (74/99) | 25% (25/99) | 1.19 [0.71-1.99] | 0.515 |  |  |  |  |  |  |  |  |  |  |  |  |  |  |
| *Senior* | 70% (52/74) | 30% (22/74) | 1.40 [0.83-2.36] | 0.210 |  |  |  |  |  |  |  |  |  |  |  |  |  |  |
| **Gender Identity** |  |  |  |  |  |  |  |  |  |  |  |  |  |  |  |  |  |  |
| *Cisgender woman* | 77% (245/319) | 23% (74/319) | 1.00 [ref] |  | 1.00 [ref] |  | 1.00 [ref] |  | 1.00 [ref] |  | 1.00 [ref] |  | 1.00 [ref] |  | 1.00 [ref] |  | 1.00 [ref] |  |
| *Transgender/Gender non-conforming* | 47% (7/15) | 53% (8/15) | 2.30 [1.38-3.84] | **0.001** | 1.86 [1.08-3.21] | **0.026** | 1.65 [0.93-2.92] | 0.084 | 1.82 [1.11-2.99] | **0.017** | 1.83 [1.16-2.88] | **0.009** | 1.82 [1.18-2.81] | **0.007** | 1.96 [1.27-3.02] | **0.002** | 1.90 [1.21-2.96] | **0.005** |
| **Received need-based financial aid** |  |  |  |  |  |  |  |  |  |  |  |  |  |  |  |  |  |  |
| *No* | 84% (156/186) | 16% (30/186) | 1.00 [ref] |  | 1.00 [ref] |  | 1.00 [ref] |  | 1.00 [ref] |  | 1.00 [ref] |  | 1.00 [ref] |  | 1.00 [ref] |  | 1.00 [ref] |  |
| *Yes* | 64% (86/135) | 36% (49/135) | 2.25 [1.51-3.35] | **<0.001** | 1.93 [1.27-2.95] | **0.002** | 1.91 [1.26-2.91] | **0.002** | 1.88 [1.23-2.87] | **0.003** | 1.96 [1.29-2.98] | **0.002** | 2.11 [1.40-3.19] | **<0.001** | 2.16 [1.44-3.23] | **<0.001** | 2.07 [1.39-3.10] | **<0.001** |
| **Lives with...** |  |  |  |  |  |  |  |  |  |  |  |  |  |  |  |  |  |  |
| *Family* | 85% (55/65) | 15% (10/65) | 1.00 [ref] |  | 1.00 [ref] |  | 1.00 [ref] |  | 1.00 [ref] |  | 1.00 [ref] |  | 1.00 [ref] |  | 1.00 [ref] |  | 1.00 [ref] |  |
| *Friends/Roommate(s)/Significant other* | 76% (184/243) | 24% (59/243) | 1.58 [0.86-2.91] | 0.144 | 1.43 [0.66-3.09] | 0.367 | 1.40 [0.64-3.03] | 0.397 | 1.45 [0.68-3.08] | 0.338 | 1.58 [0.79-3.15] | 0.195 | 1.28 [0.67-2.42] | 0.455 | 1.31 [0.68-2.49] | 0.419 | 1.56 [0.86-2.83] | 0.139 |
| *Alone* | 50% (13/26) | 50% (13/26) | 3.25 [1.63-6.46] | **<0.001** | 2.71 [1.18-6.21] | **0.019** | 2.67 [1.17-6.13] | **0.020** | 2.70 [1.18-6.17] | **0.018** | 3.00 [1.42-6.35] | **0.004** | 2.37 [1.20-4.71] | **0.013** | 2.38 [1.20-4.72] | **0.013** | 2.83 [1.41-5.66] | **0.003** |
| **Location** |  |  |  |  |  |  |  |  |  |  |  |  |  |  |  |  |  |  |
| *Outside NYC metro area* | 86% (48/56) | 14% (8/56) | 1.00 [ref] |  | 1.00 [ref] |  | 1.00 [ref] |  | 1.00 [ref] |  | 1.00 [ref] |  | 1.00 [ref] |  | 1.00 [ref] |  |  |  |
| *Off-campus NYC metro area* | 72% (119/166) | 28% (47/166) | 1.98 [1.00-3.93] | 0.051 | 2.34 [0.94-5.83] | 0.068 | 2.32 [0.93-5.78] | 0.072 | 2.31 [0.94-5.69] | 0.069 | 2.02 [0.88-4.61] | 0.095 | 1.89 [0.87-4.12] | 0.107 | 1.88 [0.86-4.13] | 0.113 |  |  |
| *On-campus housing* | 76% (85/112) | 24% (27/112) | 1.69 [0.82-3.47] | 0.155 | 1.72 [0.61-4.87] | 0.307 | 1.75 [0.62-4.93] | 0.288 | 1.71 [0.62-4.68] | 0.299 | 1.37 [0.54-3.49] | 0.506 | 1.41 [0.60-3.30] | 0.431 | 1.38 [0.58-3.26] | 0.462 |  |  |
| **Home perceived unsafe** |  |  |  |  |  |  |  |  |  |  |  |  |  |  |  |  |  |  |
| *No* | 79% (188/237) | 21% (49/237) | 1.00 [ref] |  | 1.00 [ref] |  | 1.00 [ref] |  | 1.00 [ref] |  | 1.00 [ref] |  | 1.00 [ref] |  | 1.00 [ref] |  | 1.00 [ref] |  |
| *Yes* | 67% (64/95) | 33% (31/95) | 1.58 [1.08-2.31] | **0.019** | 1.43 [0.95-2.14] | 0.085 | 1.39 [0.94-2.05] | 0.096 | 1.40 [0.95-2.07] | 0.085 | 1.41 [0.98-2.05] | 0.068 | 1.48 [1.03-2.14] | **0.036** | 1.53 [1.06-2.20] | **0.022** | 1.59 [1.09-2.31] | **0.015** |
| **Social group involvement** |  |  |  |  |  |  |  |  |  |  |  |  |  |  |  |  |  |  |
| *No* | 78% (131/168) | 22% (37/168) | 1.00 [ref] |  |  |  |  |  |  |  |  |  |  |  |  |  |  |  |
| *Yes* | 72% (118/163) | 28% (45/163) | 1.25 [0.86-1.83] | 0.241 |  |  |  |  |  |  |  |  |  |  |  |  |  |  |
| **Sports involvement** |  |  |  |  |  |  |  |  |  |  |  |  |  |  |  |  |  |  |
| *No* | 75% (236/314) | 25% (78/314) | 1.00 [ref] |  |  |  |  |  |  |  |  |  |  |  |  |  |  |  |
| *Yes* | 80% (16/20) | 20% (4/20) | 0.81 [0.33-1.98] | 0.636 |  |  |  |  |  |  |  |  |  |  |  |  |  |  |
| **Relationship status** |  |  |  |  |  |  |  |  |  |  |  |  |  |  |  |  |  |  |
| *Single* | 77% (178/230) | 23% (52/230) | 1.00 [ref] |  |  |  |  |  |  |  |  |  |  |  |  |  |  |  |
| *In some form of relationship* | 72% (73/102) | 28% (29/102) | 1.26 [0.85-1.86] | 0.249 |  |  |  |  |  |  |  |  |  |  |  |  |  |  |
| **Experienced physical/verbal violence** |  |  |  |  |  |  |  |  |  |  |  |  |  |  |  |  |  |  |
| *No* | 79% (176/223) | 21% (47/223) | 1.00 [ref] |  |  |  |  |  |  |  |  |  |  |  |  |  |  |  |
| *Yes* | 71% (67/94) | 29% (27/94) | 1.36 [0.91-2.05] | 0.136 |  |  |  |  |  |  |  |  |  |  |  |  |  |  |
| **Condom use status** |  |  |  |  |  |  |  |  |  |  |  |  |  |  |  |  |  |  |
| *No sexual activity* | 79% (168/213) | 21% (45/213) | 1.00 [ref] |  | 1.00 [ref] |  | 1.00 [ref] |  | 1.00 [ref] |  |  |  |  |  |  |  |  |  |
| *No condoms* | 67% (34/51) | 33% (17/51) | 1.58 [0.99-2.52] | 0.056 | 1.33 [0.78-2.28] | 0.292 | 1.37 [0.82-2.28] | 0.229 | 1.44 [0.88-2.37] | 0.150 |  |  |  |  |  |  |  |  |
| *Used condoms* | 75% (44/59) | 25% (15/59) | 1.20 [0.72-2.00] | 0.475 | 1.36 [0.79-2.34] | 0.265 | 1.27 [0.75-2.15] | 0.383 | 1.31 [0.77-2.22] | 0.313 |  |  |  |  |  |  |  |  |
| **Currently smokes/vapes** |  |  |  |  |  |  |  |  |  |  |  |  |  |  |  |  |  |  |
| *No* | 77% (238/309) | 23% (71/309) | 1.00 [ref] |  | 1.00 [ref] |  | 1.00 [ref] |  |  |  |  |  |  |  |  |  |  |  |
| *Yes* | 56% (14/25) | 44% (11/25) | 1.91 [1.18-3.12] | **0.009** | 1.52 [0.81-2.87] | 0.193 | 1.37 [0.73-2.59] | 0.327 |  |  |  |  |  |  |  |  |  |  |
| **Frequency of alcohol use** |  |  |  |  |  |  |  |  |  |  |  |  |  |  |  |  |  |  |
| *Never/Rare* | 77% (169/219) | 23% (50/219) | 1.00 [ref] |  |  |  |  |  |  |  |  |  |  |  |  |  |  |  |
| *Moderate/High* | 72% (82/114) | 28% (32/114) | 1.23 [0.84-1.80] | 0.289 |  |  |  |  |  |  |  |  |  |  |  |  |  |  |
| **Frequency of drug use** |  |  |  |  |  |  |  |  |  |  |  |  |  |  |  |  |  |  |
| *Never/Rare* | 78% (201/259) | 22% (58/259) | 1.00 [ref] |  |  |  |  |  |  |  |  |  |  |  |  |  |  |  |
| *Monthly/Weekly/Daily* | 68% (50/73) | 32% (23/73) | 1.41 [0.94-2.11] | 0.1 |  |  |  |  |  |  |  |  |  |  |  |  |  |  |
| **Since 3 months ago, feel...** |  |  |  |  |  |  |  |  |  |  |  |  |  |  |  |  |  |  |
| *Less lonely* | 77% (115/150) | 23% (35/150) | 1.00 [ref] |  |  |  |  |  |  |  |  |  |  |  |  |  |  |  |
| *Same* | 74% (56/76) | 26% (20/76) | 1.13 [0.70-1.81] | 0.62 |  |  |  |  |  |  |  |  |  |  |  |  |  |  |
| *Lonelier* | 75% (81/108) | 25% (27/108) | 1.07 [0.69-1.66] | 0.757 |  |  |  |  |  |  |  |  |  |  |  |  |  |  |
| **Had a social support network** |  |  |  |  |  |  |  |  |  |  |  |  |  |  |  |  |  |  |
| *Yes* | 78% (197/251) | 22% (54/251) | 1.00 [ref] |  | 1.00 [ref] |  |  |  |  |  |  |  |  |  |  |  |  |  |
| *Uncertain* | 67% (32/48) | 33% (16/48) | 1.55 [0.97-2.47] | 0.065 | 1.06 [0.64-1.74] | 0.821 |  |  |  |  |  |  |  |  |  |  |  |  |
| *No* | 68% (23/34) | 32% (11/34) | 1.50 [0.88-2.58] | 0.139 | 1.09 [0.61-1.96] | 0.777 |  |  |  |  |  |  |  |  |  |  |  |  |
| **Moderate-severe psychological distress** |  |  |  |  |  |  |  |  |  |  |  |  |  |  |  |  |  |  |
| *No* | 83% (94/113) | 17% (19/113) | 1.00 [ref] |  | 1.00 [ref] |  | 1.00 [ref] |  | 1.00 [ref] |  | 1.00 [ref] |  | 1.00 [ref] |  |  |  |  |  |
| *Yes* | 72% (158/220) | 28% (62/220) | 1.68 [1.06-2.66] | **0.028** | 1.49 [0.93-2.37] | 0.096 | 1.49 [0.93-2.38] | 0.094 | 1.50 [0.94-2.40] | 0.088 | 1.52 [0.96-2.40] | 0.075 | 1.39 [0.88-2.17] | 0.154 |  |  |  |  |
| **Currently using hormones** |  |  |  |  |  |  |  |  |  |  |  |  |  |  |  |  |  |  |
| *No* | 75% (156/209) | 25% (53/209) | 1.00 [ref] |  |  |  |  |  |  |  |  |  |  |  |  |  |  |  |
| *Yes* | 77% (96/124) | 23% (28/124) | 0.89 [0.60-1.33] | 0.57 |  |  |  |  |  |  |  |  |  |  |  |  |  |  |
| **Sought care for COVID-19 symptoms** |  |  |  |  |  |  |  |  |  |  |  |  |  |  |  |  |  |  |
| *No symptoms* | 75% (224/298) | 25% (74/298) | 1.00 [ref] |  |  |  |  |  |  |  |  |  |  |  |  |  |  |  |
| *Sought healthcare* | 79% (15/19) | 21% (4/19) | 0.85 [0.35-2.07] | 0.717 |  |  |  |  |  |  |  |  |  |  |  |  |  |  |
| *Self-isolated* | 67% (6/9) | 33% (3/9) | 1.34 [0.52-3.45] | 0.541 |  |  |  |  |  |  |  |  |  |  |  |  |  |  |
| *No healthcare/isolation* | 83% (5/6) | 17% (1/6) | 0.67 [0.11-4.06] | 0.664 |  |  |  |  |  |  |  |  |  |  |  |  |  |  |

PAM = Preliminary Adjusted Model, PR = Prevalence Ratio, APR = Adjusted Prevalence Ratio

Green coloring used to highlight factors with p≤0.05

# Supplemental Material 10. All performed analyses to identify correlates of food insecurity at T3

|  | **Food insecure** | | **Unadjusted** | | **PAM 1** | | **PAM 2** | | **PAM 3** | | **PAM 4** | | **PAM 5** | | **Final model** | |
| --- | --- | --- | --- | --- | --- | --- | --- | --- | --- | --- | --- | --- | --- | --- | --- | --- |
| **Characteristic** | **No, N = 185** | **Yes, N = 37** | **PR [95% CI]** | **p-value** | **APR [95% CI]** | **p-value** | **APR [95% CI]** | **p-value** | **APR [95% CI]** | **p-value** | **APR [95% CI]** | **p-value** | **APR [95% CI]** | **p-value** | **APR [95% CI]** | **p-value** |
| **Age (Continuous)** | 20 (19-20) | 20 (19-20) | 0.97 [0.77-1.22] | 0.774 |  |  |  |  |  |  |  |  |  |  |  |  |
| **Racial identity** |  |  |  |  |  |  |  |  |  |  |  |  |  |  |  |  |
| *White* | 85% (115/136) | 15% (21/136) | 1.00 [ref] |  | 1.00 [ref] |  | 1.00 [ref] |  |  |  |  |  |  |  |  |  |
| *Asian/Asian American* | 87% (41/47) | 13% (6/47) | 0.83 [0.36-1.92] | 0.659 | 0.81 [0.37-1.77] | 0.605 | 0.82 [0.38-1.79] | 0.617 |  |  |  |  |  |  |  |  |
| *Other/Multiracial* | 72% (23/32) | 28% (9/32) | 1.82 [0.92-3.59] | 0.084 | 1.34 [0.68-2.65] | 0.404 | 1.34 [0.67-2.67] | 0.402 |  |  |  |  |  |  |  |  |
| **Hispanic ethnicity** |  |  |  |  |  |  |  |  |  |  |  |  |  |  |  |  |
| *No* | 84% (160/190) | 16% (30/190) | 1.00 [ref] |  |  |  |  |  |  |  |  |  |  |  |  |  |
| *Yes* | 77% (20/26) | 23% (6/26) | 1.46 [0.67-3.17] | 0.337 |  |  |  |  |  |  |  |  |  |  |  |  |
| **School year** |  |  |  |  |  |  |  |  |  |  |  |  |  |  |  |  |
| *First-year* | 82% (55/67) | 18% (12/67) | 1.00 [ref] |  |  |  |  |  |  |  |  |  |  |  |  |  |
| *Sophomore* | 85% (39/46) | 15% (7/46) | 0.85 [0.36-1.99] | 0.708 |  |  |  |  |  |  |  |  |  |  |  |  |
| *Junior* | 83% (60/72) | 17% (12/72) | 0.93 [0.45-1.93] | 0.846 |  |  |  |  |  |  |  |  |  |  |  |  |
| *Senior* | 84% (31/37) | 16% (6/37) | 0.91 [0.37-2.21] | 0.828 |  |  |  |  |  |  |  |  |  |  |  |  |
| **Gender Identity** |  |  |  |  |  |  |  |  |  |  |  |  |  |  |  |  |
| *Cisgender woman* | 84% (176/210) | 16% (34/210) | 1.00 [ref] |  |  |  |  |  |  |  |  |  |  |  |  |  |
| *Transgender/Gender non-conforming* | 75% (9/12) | 25% (3/12) | 1.54 [0.55-4.31] | 0.407 |  |  |  |  |  |  |  |  |  |  |  |  |
| **Received need-based financial aid** |  |  |  |  |  |  |  |  |  |  |  |  |  |  |  |  |
| *No* | 91% (112/123) | 9% (11/123) | 1.00 [ref] |  | 1.00 [ref] |  | 1.00 [ref] |  | 1.00 [ref] |  | 1.00 [ref] |  | 1.00 [ref] |  | 1.00 [ref] |  |
| *Yes* | 72% (65/90) | 28% (25/90) | 3.11 [1.61-5.98] | **<0.001** | 2.35 [1.30-4.22] | **0.004** | 2.29 [1.27-4.13] | **0.006** | 2.47 [1.33-4.57] | **0.004** | 2.74 [1.48-5.08] | **0.001** | 2.77 [1.51-5.11] | **0.001** | 2.73 [1.46-5.11] | **0.002** |
| **Lives with...** |  |  |  |  |  |  |  |  |  |  |  |  |  |  |  |  |
| *Family* | 86% (96/111) | 14% (15/111) | 1.00 [ref] |  |  |  |  |  |  |  |  |  |  |  |  |  |
| *Friends/Roommate(s)/Significant other* | 81% (75/93) | 19% (18/93) | 1.43 [0.76-2.68] | 0.262 |  |  |  |  |  |  |  |  |  |  |  |  |
| *Alone* | 77% (10/13) | 23% (3/13) | 1.71 [0.57-5.12] | 0.340 |  |  |  |  |  |  |  |  |  |  |  |  |
| **Location** |  |  |  |  |  |  |  |  |  |  |  |  |  |  |  |  |
| *Outside NYC metro area* | 90% (80/89) | 10% (9/89) | 1.00 [ref] |  | 1.00 [ref] |  | 1.00 [ref] |  | 1.00 [ref] |  | 1.00 [ref] |  | 1.00 [ref] |  |  |  |
| *Off-campus NYC metro area* | 81% (79/97) | 19% (18/97) | 1.84 [0.87-3.87] | 0.111 | 1.72 [0.78-3.83] | 0.181 | 1.65 [0.80-3.43] | 0.176 | 1.71 [0.82-3.57] | 0.156 | 1.61 [0.80-3.22] | 0.181 | 1.64 [0.82-3.25] | 0.161 |  |  |
| *On-campus housing* | 72% (26/36) | 28% (10/36) | 2.75 [1.22-6.19] | **0.015** | 1.96 [0.81-4.77] | 0.137 | 1.89 [0.83-4.29] | 0.130 | 2.26 [1.01-5.02] | **0.047** | 2.03 [0.94-4.38] | 0.071 | 1.89 [0.88-4.04] | 0.101 |  |  |
| **Home perceived unsafe** |  |  |  |  |  |  |  |  |  |  |  |  |  |  |  |  |
| *No* | 87% (148/170) | 13% (22/170) | 1.00 [ref] |  | 1.00 [ref] |  | 1.00 [ref] |  | 1.00 [ref] |  | 1.00 [ref] |  |  |  |  |  |
| *Yes* | 71% (35/49) | 29% (14/49) | 2.21 [1.22-3.98] | **0.009** | 1.42 [0.75-2.68] | 0.278 | 1.41 [0.75-2.66] | 0.284 | 1.45 [0.78-2.72] | 0.242 | 1.35 [0.71-2.54] | 0.356 |  |  |  |  |
| **Social group involvement** |  |  |  |  |  |  |  |  |  |  |  |  |  |  |  |  |
| *No* | 87% (116/133) | 13% (17/133) | 1.00 [ref] |  | 1.00 [ref] |  | 1.00 [ref] |  | 1.00 [ref] |  |  |  |  |  |  |  |
| *Yes* | 78% (67/86) | 22% (19/86) | 1.73 [0.95-3.14] | 0.072 | 1.42 [0.79-2.57] | 0.244 | 1.42 [0.79-2.55] | 0.247 | 1.34 [0.75-2.40] | 0.322 |  |  |  |  |  |  |
| **Sports involvement** |  |  |  |  |  |  |  |  |  |  |  |  |  |  |  |  |
| *No* | 83% (176/212) | 17% (36/212) | 1.00 [ref] |  |  |  |  |  |  |  |  |  |  |  |  |  |
| *Yes* | 90% (9/10) | 10% (1/10) | 0.59 [0.09-3.87] | 0.582 |  |  |  |  |  |  |  |  |  |  |  |  |
| **Relationship status** |  |  |  |  |  |  |  |  |  |  |  |  |  |  |  |  |
| *Single* | 82% (119/146) | 18% (27/146) | 1.00 [ref] |  |  |  |  |  |  |  |  |  |  |  |  |  |
| *In some form of relationship* | 87% (65/75) | 13% (10/75) | 0.72 [0.37-1.41] | 0.339 |  |  |  |  |  |  |  |  |  |  |  |  |
| **Experienced physical/verbal violence** |  |  |  |  |  |  |  |  |  |  |  |  |  |  |  |  |
| *No* | 85% (123/144) | 15% (21/144) | 1.00 [ref] |  |  |  |  |  |  |  |  |  |  |  |  |  |
| *Yes* | 82% (54/66) | 18% (12/66) | 1.25 [0.65-2.38] | 0.504 |  |  |  |  |  |  |  |  |  |  |  |  |
| **Condom use status** |  |  |  |  |  |  |  |  |  |  |  |  |  |  |  |  |
| *No sexual activity* | 83% (98/118) | 17% (20/118) | 1.00 [ref] |  |  |  |  |  |  |  |  |  |  |  |  |  |
| *No condoms* | 86% (30/35) | 14% (5/35) | 0.84 [0.34-2.08] | 0.711 |  |  |  |  |  |  |  |  |  |  |  |  |
| *Used condoms* | 89% (48/54) | 11% (6/54) | 0.66 [0.28-1.54] | 0.332 |  |  |  |  |  |  |  |  |  |  |  |  |
| **Currently smokes/vapes** |  |  |  |  |  |  |  |  |  |  |  |  |  |  |  |  |
| *No* | 85% (168/198) | 15% (30/198) | 1.00 [ref] |  | 1.00 [ref] |  |  |  |  |  |  |  |  |  |  |  |
| *Yes* | 71% (15/21) | 29% (6/21) | 1.89 [0.89-4.00] | 0.098 | 1.34 [0.58-3.09] | 0.496 |  |  |  |  |  |  |  |  |  |  |
| **Frequency of alcohol use** |  |  |  |  |  |  |  |  |  |  |  |  |  |  |  |  |
| *Never/Rare* | 81% (112/138) | 19% (26/138) | 1.00 [ref] |  |  |  |  |  |  |  |  |  |  |  |  |  |
| *Moderate/High* | 87% (73/84) | 13% (11/84) | 0.70 [0.36-1.33] | 0.273 |  |  |  |  |  |  |  |  |  |  |  |  |
| **Frequency of drug use** |  |  |  |  |  |  |  |  |  |  |  |  |  |  |  |  |
| *Never/Rare* | 86% (150/174) | 14% (24/174) | 1.00 [ref] |  | 1.00 [ref] |  | 1.00 [ref] |  | 1.00 [ref] |  | 1.00 [ref] |  | 1.00 [ref] |  | 1.00 [ref] |  |
| *Monthly/Weekly/Daily* | 72% (34/47) | 28% (13/47) | 2.01 [1.11-3.63] | **0.021** | 1.64 [0.88-3.05] | 0.121 | 1.82 [1.00-3.32] | **0.049** | 2.08 [1.18-3.65] | **0.011** | 2.00 [1.16-3.44] | **0.013** | 1.99 [1.16-3.39] | **0.012** | 2.00 [1.15-3.48] | **0.014** |
| **Since 3 months ago, feel...** |  |  |  |  |  |  |  |  |  |  |  |  |  |  |  |  |
| *Less lonely* | 86% (107/124) | 14% (17/124) | 1.00 [ref] |  |  |  |  |  |  |  |  |  |  |  |  |  |
| *Same* | 83% (38/46) | 17% (8/46) | 1.27 [0.59-2.74] | 0.544 |  |  |  |  |  |  |  |  |  |  |  |  |
| *Lonelier* | 77% (40/52) | 23% (12/52) | 1.68 [0.87-3.27] | 0.124 |  |  |  |  |  |  |  |  |  |  |  |  |
| **Had a social support network** |  |  |  |  |  |  |  |  |  |  |  |  |  |  |  |  |
| *Yes* | 88% (151/172) | 12% (21/172) | 1.00 [ref] |  | 1.00 [ref] |  | 1.00 [ref] |  | 1.00 [ref] |  | 1.00 [ref] |  | 1.00 [ref] |  | 1.00 [ref] |  |
| *Uncertain* | 71% (17/24) | 29% (7/24) | 2.39 [1.14-5.01] | **0.021** | 1.70 [0.70-4.16] | 0.241 | 1.72 [0.70-4.20] | 0.233 | 1.84 [0.84-4.04] | 0.125 | 1.98 [0.97-4.07] | 0.062 | 2.13 [1.07-4.24] | **0.032** | 2.07 [1.07-4.01] | **0.030** |
| *No* | 63% (15/24) | 38% (9/24) | 3.07 [1.60-5.91] | **<0.001** | 1.83 [0.89-3.76] | 0.101 | 1.89 [0.91-3.92] | 0.089 | 1.86 [0.91-3.78] | 0.088 | 1.97 [0.95-4.11] | 0.069 | 2.32 [1.26-4.30] | **0.007** | 2.56 [1.35-4.85] | **0.004** |
| **Moderate-severe psychological distress** |  |  |  |  |  |  |  |  |  |  |  |  |  |  |  |  |
| *No* | 91% (92/101) | 9% (9/101) | 1.00 [ref] |  | 1.00 [ref] |  | 1.00 [ref] |  | 1.00 [ref] |  | 1.00 [ref] |  | 1.00 [ref] |  | 1.00 [ref] |  |
| *Yes* | 76% (91/119) | 24% (28/119) | 2.64 [1.31-5.33] | **0.007** | 1.83 [0.81-4.15] | 0.147 | 1.93 [0.87-4.30] | 0.108 | 1.81 [0.87-3.76] | 0.111 | 1.97 [0.97-4.01] | 0.061 | 2.15 [1.07-4.34] | **0.033** | 2.12 [1.04-4.33] | **0.038** |
| **Currently using hormones** |  |  |  |  |  |  |  |  |  |  |  |  |  |  |  |  |
| *No* | 81% (108/133) | 19% (25/133) | 1.00 [ref] |  |  |  |  |  |  |  |  |  |  |  |  |  |
| *Yes* | 87% (77/89) | 13% (12/89) | 0.72 [0.38-1.35] | 0.304 |  |  |  |  |  |  |  |  |  |  |  |  |
| **Sought care for COVID-19 symptoms** |  |  |  |  |  |  |  |  |  |  |  |  |  |  |  |  |
| *No symptoms* | 84% (168/200) | 16% (32/200) | † |  |  |  |  |  |  |  |  |  |  |  |  |  |
| *Sought healthcare* | 100% (9/9) | 0% (0/9) | † |  |  |  |  |  |  |  |  |  |  |  |  |  |
| *Self-isolated* | 0% (0/3) | 100% (3/3) | † |  |  |  |  |  |  |  |  |  |  |  |  |  |
| *No healthcare/isolation* | 80% (8/10) | 20% (2/10) | † |  |  |  |  |  |  |  |  |  |  |  |  |  |

PAM = Preliminary Adjusted Model, PR = Prevalence Ratio, APR = Adjusted Prevalence Ratio

Green coloring used to highlight factors with p≤0.05

† Unadjusted model not fitted due to presence of cells with 0 values

# Supplemental Material 11. All performed analyses to identify correlates of food insecurity at T4

|  | **Food insecure** | | **Unadjusted** | | **Without violence fitted in model** | | | | | | | | **With violence  fitted in model^‡^** | |
| --- | --- | --- | --- | --- | --- | --- | --- | --- | --- | --- | --- | --- | --- | --- |
|  |  |  |  |  | **PAM 1** | | **PAM 2** | | **PAM 3** | | **Final model** | | **PAM 1** | |
| **Characteristic** | **No, N = 135** | **Yes, N = 32** | **PR [95% CI]** | **p-value** | **APR [95% CI]** | **p-value** | **APR [95% CI]** | **p-value** | **APR [95% CI]** | **p-value** | **APR [95% CI]** | **p-value** | **APR [95% CI]** | **p-value** |
| **Age (Continuous)** | 19 (19-20) | 20 (19-21) | 1.16 [0.90-1.50] | 0.260 |  |  |  |  |  |  |  |  |  |  |
| **Racial identity** |  |  |  |  |  |  |  |  |  |  |  |  |  |  |
| *White* | 80% (84/105) | 20% (21/105) | 1.00 [ref] |  |  |  |  |  |  |  |  |  |  |  |
| *Asian/Asian American* | 81% (30/37) | 19% (7/37) | 0.95 [0.44-2.04] | 0.887 |  |  |  |  |  |  |  |  |  |  |
| *Other/Multiracial* | 85% (17/20) | 15% (3/20) | 0.75 [0.25-2.28] | 0.612 |  |  |  |  |  |  |  |  |  |  |
| **Hispanic ethnicity** |  |  |  |  |  |  |  |  |  |  |  |  |  |  |
| *No* | 80% (115/143) | 20% (28/143) | 1.00 [ref] |  |  |  |  |  |  |  |  |  |  |  |
| *Yes* | 81% (17/21) | 19% (4/21) | 0.97 [0.38-2.50] | 0.954 |  |  |  |  |  |  |  |  |  |  |
| **School year** |  |  |  |  |  |  |  |  |  |  |  |  |  |  |
| *First-year* | 88% (45/51) | 12% (6/51) | 1.00 [ref] |  | 1.00 [ref] |  | 1.00 [ref] |  | 1.00 [ref] |  |  |  | 1.00 [ref] |  |
| *Sophomore* | 79% (30/38) | 21% (8/38) | 1.79 [0.68-4.73] | 0.240 | 1.84 [0.69-4.86] | 0.22 | 1.45 [0.56-3.74] | 0.44 | 1.52 [0.59-3.92] | 0.382 |  |  | 1.64 [0.56-4.84] | 0.367 |
| *Junior* | 83% (44/53) | 17% (9/53) | 1.44 [0.55-3.77] | 0.453 | 1.64 [0.69-3.85] | 0.26 | 1.34 [0.58-3.09] | 0.489 | 1.32 [0.56-3.11] | 0.525 |  |  | 1.37 [0.52-3.65] | 0.524 |
| *Senior* | 64% (16/25) | 36% (9/25) | 3.06 [1.23-7.64] | **0.017** | 9.60 [0.55-168] | 0.121 | 2.56 [0.87-7.52] | 0.086 | 2.46 [0.89-6.82] | 0.084 |  |  | 6.61 [0.38-114] | 0.194 |
| **Gender Identity** |  |  |  |  |  |  |  |  |  |  |  |  |  |  |
| *Cisgender woman* | 83% (130/156) | 17% (26/156) | 1.00 [ref] |  | 1.00 [ref] |  | 1.00 [ref] |  | 1.00 [ref] |  | 1.00 [ref] |  | 1.00 [ref] |  |
| *Transgender/Gender non-conforming* | 45% (5/11) | 55% (6/11) | 3.27 [1.72-6.23] | **<0.001** | 1.55 [0.85-2.81] | 0.154 | 1.57 [0.82-3.00] | 0.172 | 1.90 [1.05-3.45] | **0.034** | 2.29 [1.35-3.88] | **0.002** | 1.62 [0.80-3.27] | 0.18 |
| **Received need-based financial aid** |  |  |  |  |  |  |  |  |  |  |  |  |  |  |
| *No* | 91% (81/89) | 9% (8/89) | 1.00 [ref] |  | 1.00 [ref] |  | 1.00 [ref] |  | 1.00 [ref] |  | 1.00 [ref] |  | 1.00 [ref] |  |
| *Yes* | 68% (46/68) | 32% (22/68) | 3.60 [1.71-7.58] | **<0.001** | 2.09 [0.96-4.55] | 0.062 | 2.87 [1.34-6.13] | **0.006** | 2.82 [1.29-6.15] | **0.009** | 3.06 [1.45-6.44] | **0.003** | 1.98 [0.80-4.90] | 0.138 |
| **Lives with...** |  |  |  |  |  |  |  |  |  |  |  |  |  |  |
| *Family* | 83% (10/12) | 17% (2/12) | 1.00 [ref] |  |  |  |  |  |  |  |  |  |  |  |
| *Friends/Roommate(s)/Significant other* | 83% (99/120) | 18% (21/120) | 1.05 [0.28-3.94] | 0.942 |  |  |  |  |  |  |  |  |  |  |
| *Alone* | 70% (19/27) | 30% (8/27) | 1.78 [0.44-7.15] | 0.418 |  |  |  |  |  |  |  |  |  |  |
| **Location** |  |  |  |  |  |  |  |  |  |  |  |  |  |  |
| *Outside NYC metro area* | 55% (6/11) | 45% (5/11) | 1.00 [ref] |  | 1.00 [ref] |  |  |  |  |  |  |  | 1.00 [ref] |  |
| *Off-campus NYC metro area* | 90% (44/49) | 10% (5/49) | 0.22 [0.08-0.64] | **0.005** | 0.45 [0.08-2.51] | 0.360 |  |  |  |  |  |  | 0.50 [0.07-3.39] | 0.478 |
| *On-campus housing* | 79% (85/107) | 21% (22/107) | 0.45 [0.21-0.95] | **0.037** | 2.49 [0.12-53.5] | 0.560 |  |  |  |  |  |  | 2.02 [0.09-45.2] | 0.656 |
| **Home perceived unsafe** |  |  |  |  |  |  |  |  |  |  |  |  |  |  |
| *No* | 87% (108/124) | 13% (16/124) | 1.00 [ref] |  | 1.00 [ref] |  | 1.00 [ref] |  | 1.00 [ref] |  | 1.00 [ref] |  | 1.00 [ref] |  |
| *Yes* | 63% (27/43) | 37% (16/43) | 2.88 [1.58-5.25] | **<0.001** | 2.11 [1.17-3.80] | **0.013** | 2.15 [1.23-3.77] | **0.007** | 2.35 [1.33-4.17] | **0.003** | 2.32 [1.31-4.11] | **0.004** | 2.15 [1.10-4.18] | **0.025** |
| **Social group involvement** |  |  |  |  |  |  |  |  |  |  |  |  |  |  |
| *No* | 79% (52/66) | 21% (14/66) | 1.00 [ref] |  |  |  |  |  |  |  |  |  |  |  |
| *Yes* | 82% (82/100) | 18% (18/100) | 0.85 [0.45-1.59] | 0.607 |  |  |  |  |  |  |  |  |  |  |
| **Sports involvement** |  |  |  |  |  |  |  |  |  |  |  |  |  |  |
| *No* | 81% (124/154) | 19% (30/154) | 1.00 [ref] |  |  |  |  |  |  |  |  |  |  |  |
| *Yes* | 85% (11/13) | 15% (2/13) | 0.79 [0.21-2.94] | 0.725 |  |  |  |  |  |  |  |  |  |  |
| **Relationship status** |  |  |  |  |  |  |  |  |  |  |  |  |  |  |
| *Single* | 81% (90/111) | 19% (21/111) | 1.00 [ref] |  |  |  |  |  |  |  |  |  |  |  |
| *In some form of relationship* | 80% (45/56) | 20% (11/56) | 1.04 [0.54-2.00] | 0.911 |  |  |  |  |  |  |  |  |  |  |
| **Experienced physical/verbal violence** |  |  |  |  |  |  |  |  |  |  |  |  |  |  |
| *No* | 85% (103/121) | 15% (18/121) | 1.00 [ref] |  |  |  |  |  |  |  |  |  | 1.00 [ref] |  |
| *Yes* | 67% (14/21) | 33% (7/21) | 2.24 [1.07-4.70] | **0.033** |  |  |  |  |  |  |  |  | 1.04 [0.41-2.62] | 0.942 |
| **Condom use status** |  |  |  |  |  |  |  |  |  |  |  |  |  |  |
| *No sexual activity* | 84% (71/85) | 16% (14/85) | 1.00 [ref] |  |  |  |  |  |  |  |  |  |  |  |
| *No condoms* | 79% (22/28) | 21% (6/28) | 1.30 [0.55-3.06] | 0.547 |  |  |  |  |  |  |  |  |  |  |
| *Used condoms* | 83% (40/48) | 17% (8/48) | 1.01 [0.46-2.24] | 0.977 |  |  |  |  |  |  |  |  |  |  |
| **Currently smokes/vapes** |  |  |  |  |  |  |  |  |  |  |  |  |  |  |
| *No* | 82% (126/154) | 18% (28/154) | 1.00 [ref] |  |  |  |  |  |  |  |  |  |  |  |
| *Yes* | 64% (7/11) | 36% (4/11) | 2.00 [0.85-4.68] | 0.110 |  |  |  |  |  |  |  |  |  |  |
| **Frequency of alcohol use** |  |  |  |  |  |  |  |  |  |  |  |  |  |  |
| *Never/Rare* | 80% (82/103) | 20% (21/103) | 1.00 [ref] |  |  |  |  |  |  |  |  |  |  |  |
| *Moderate/High* | 83% (52/63) | 17% (11/63) | 0.86 [0.44-1.65] | 0.645 |  |  |  |  |  |  |  |  |  |  |
| **Frequency of drug use** |  |  |  |  |  |  |  |  |  |  |  |  |  |  |
| *Never/Rare* | 83% (107/129) | 17% (22/129) | 1.00 [ref] |  |  |  |  |  |  |  |  |  |  |  |
| *Monthly/Weekly/Daily* | 73% (27/37) | 27% (10/37) | 1.58 [0.83-3.04] | 0.166 |  |  |  |  |  |  |  |  |  |  |
| **Since 3 months ago, feel...** |  |  |  |  |  |  |  |  |  |  |  |  |  |  |
| *Less lonely* | 83% (88/106) | 17% (18/106) | 1.00 [ref] |  |  |  |  |  |  |  |  |  |  |  |
| *Same* | 82% (27/33) | 18% (6/33) | 1.07 [0.46-2.47] | 0.873 |  |  |  |  |  |  |  |  |  |  |
| *Lonelier* | 71% (20/28) | 29% (8/28) | 1.68 [0.82-3.46] | 0.157 |  |  |  |  |  |  |  |  |  |  |
| **Had a social support network** |  |  |  |  |  |  |  |  |  |  |  |  |  |  |
| *Yes* | 81% (110/136) | 19% (26/136) | 1.00 [ref] |  |  |  |  |  |  |  |  |  |  |  |
| *Uncertain* | 81% (17/21) | 19% (4/21) | 1.00 [0.39-2.57] | 0.994 |  |  |  |  |  |  |  |  |  |  |
| *No* | 88% (7/8) | 13% (1/8) | 0.65 [0.10-4.22] | 0.655 |  |  |  |  |  |  |  |  |  |  |
| **Moderate-severe psychological distress** |  |  |  |  |  |  |  |  |  |  |  |  |  |  |
| *No* | 89% (66/74) | 11% (8/74) | 1.00 [ref] |  | 1.00 [ref] |  | 1.00 [ref] |  |  |  |  |  | 1.00 [ref] |  |
| *Yes* | 76% (69/91) | 24% (22/91) | 2.24 [1.06-4.73] | **0.035** | 1.72 [0.78-3.78] | 0.178 | 1.70 [0.77-3.73] | 0.188 |  |  |  |  | 1.31 [0.54-3.15] | 0.551 |
| **Currently using hormones** |  |  |  |  |  |  |  |  |  |  |  |  |  |  |
| *No* | 81% (75/93) | 19% (18/93) | 1.00 [ref] |  |  |  |  |  |  |  |  |  |  |  |
| *Yes* | 81% (59/73) | 19% (14/73) | 0.99 [0.53-1.86] | 0.977 |  |  |  |  |  |  |  |  |  |  |
| **Sought care for COVID-19 symptoms** |  |  |  |  |  |  |  |  |  |  |  |  |  |  |
| *No symptoms* | 82% (102/125) | 18% (23/125) | † |  |  |  |  |  |  |  |  |  |  |  |
| *Sought healthcare* | 67% (10/15) | 33% (5/15) | † |  |  |  |  |  |  |  |  |  |  |  |
| *Self-isolated* | 70% (7/10) | 30% (3/10) | † |  |  |  |  |  |  |  |  |  |  |  |
| *No healthcare/isolation* | 100% (7/7) | 0% (0/7) | † |  |  |  |  |  |  |  |  |  |  |  |

PAM = Preliminary Adjusted Model, PR = Prevalence Ratio, APR = Adjusted Prevalence Ratio

Violence model was fitted separately because inclusion of the violence variable then restricts the underlying sample to those in a relationship or cohabitating with others (family or friends).

Green coloring used to highlight factors with p≤0.05

† Unadjusted model not fitted due to presence of cells with 0 values

‡ PAM with violence not fitted beyond 1 because violence would have been the first removed variable, making PAM 2 (with violence) identical to PAM 1 (without violence)

# Supplemental Material 12. Trajectory sample characteristics, over time

| **Characteristic** | **T1, N = 167** | **T2, N = 167** | **T3, N = 167** | **T4, N = 167** | **p-value** |
| --- | --- | --- | --- | --- | --- |
| **Age (Continuous)** | 20 (19-20) | 20 (19-20) | 20 (19-20) | 20 (19-20) | >0.999 |
| **Racial identity** |  |  |  |  | >0.999 |
| *White* | 65% (105/162) | 65% (105/162) | 65% (105/162) | 65% (105/162) |  |
| *Asian/Asian American* | 23% (37/162) | 23% (37/162) | 23% (37/162) | 23% (37/162) |  |
| *Other/Multiracial* | 12% (20/162) | 12% (20/162) | 12% (20/162) | 12% (20/162) |  |
| **Hispanic ethnicity** |  |  |  |  | >0.999 |
| *No* | 87% (143/164) | 87% (143/164) | 87% (143/164) | 87% (143/164) |  |
| *Yes* | 13% (21/164) | 13% (21/164) | 13% (21/164) | 13% (21/164) |  |
| **School year** |  |  |  |  | >0.999 |
| *First-year* | 31% (51/167) | 31% (51/167) | 31% (51/167) | 31% (51/167) |  |
| *Sophomore* | 23% (38/167) | 23% (38/167) | 23% (38/167) | 23% (38/167) |  |
| *Junior* | 32% (53/167) | 32% (53/167) | 32% (53/167) | 32% (53/167) |  |
| *Senior* | 15% (25/167) | 15% (25/167) | 15% (25/167) | 15% (25/167) |  |
| **Gender Identity** |  |  |  |  | 0.549 |
| *Cisgender woman* | 96% (161/167) | 95% (159/167) | 93% (156/167) | 93% (156/167) |  |
| *Transgender/Gender non-conforming* | 4% (6/167) | 5% (8/167) | 7% (11/167) | 7% (11/167) |  |
| **Received need-based financial aid** |  |  |  |  | 0.955 |
| *No* | 60% (96/161) | 59% (93/158) | 59% (94/159) | 57% (89/157) |  |
| *Yes* | 40% (65/161) | 41% (65/158) | 41% (65/159) | 43% (68/157) |  |
| **Food insecure** |  |  |  |  | 0.496 |
| *No* | 86% (144/167) | 83% (138/167) | 86% (143/167) | 81% (135/167) |  |
| *Yes* | 14% (23/167) | 17% (29/167) | 14% (24/167) | 19% (32/167) |  |
| **Lives with...** |  |  |  |  | **<0.001** |
| *Family* | 61% (102/167) | 23% (38/167) | 54% (88/164) | 8% (12/159) |  |
| *Friends/Roommate(s)/Significant other* | 34% (57/167) | 72% (121/167) | 41% (68/164) | 75% (120/159) |  |
| *Alone* | 5% (8/167) | 5% (8/167) | 5% (8/164) | 17% (27/159) |  |
| **Location** |  |  |  |  | **<0.001** |
| *Outside NYC metro area* | 51% (85/167) | 20% (33/167) | 42% (70/167) | 7% (11/167) |  |
| *Off-campus NYC metro area* | 46% (77/167) | 40% (67/167) | 43% (71/167) | 29% (49/167) |  |
| *On-campus housing* | 3% (5/167) | 40% (67/167) | 16% (26/167) | 64% (107/167) |  |
| **Home perceived unsafe** |  |  |  |  | 0.736 |
| *No* | 75% (125/166) | 72% (120/167) | 77% (128/166) | 74% (124/167) |  |
| *Yes* | 25% (41/166) | 28% (47/167) | 23% (38/166) | 26% (43/167) |  |
| **Social group involvement** |  |  |  |  | **0.018** |
| *No* | 38% (64/167) | 45% (74/166) | 54% (89/165) | 40% (66/166) |  |
| *Yes* | 62% (103/167) | 55% (92/166) | 46% (76/165) | 60% (100/166) |  |
| **Sports involvement** |  |  |  |  | 0.217 |
| *No* | 91% (152/167) | 95% (159/167) | 96% (160/167) | 92% (154/167) |  |
| *Yes* | 9% (15/167) | 5% (8/167) | 4% (7/167) | 8% (13/167) |  |
| **Relationship status** |  |  |  |  | 0.889 |
| *Single* | 69% (114/166) | 68% (114/167) | 65% (108/166) | 66% (111/167) |  |
| *In some form of relationship* | 31% (52/166) | 32% (53/167) | 35% (58/166) | 34% (56/167) |  |
| **Experienced physical/verbal violence** |  |  |  |  | **<0.001** |
| *No* | 54% (87/160) | 70% (115/164) | 68% (108/160) | 85% (121/142) |  |
| *Yes* | 46% (73/160) | 30% (49/164) | 33% (52/160) | 15% (21/142) |  |
| **Condom use status** |  |  |  |  | **0.035** |
| *No sexual activity* | 70% (113/161) | 66% (108/164) | 56% (88/156) | 53% (85/161) |  |
| *No condoms* | 12% (19/161) | 13% (22/164) | 15% (24/156) | 17% (28/161) |  |
| *Used condoms* | 18% (29/161) | 21% (34/164) | 28% (44/156) | 30% (48/161) |  |
| **Currently smokes/vapes** |  |  |  |  | 0.289 |
| *No* | 96% (160/166) | 95% (158/167) | 91% (150/164) | 93% (154/165) |  |
| *Yes* | 4% (6/166) | 5% (9/167) | 9% (14/164) | 7% (11/165) |  |
| **Frequency of alcohol use** |  |  |  |  | 0.084 |
| *Never/Rare* | 72% (121/167) | 73% (122/166) | 66% (111/167) | 62% (103/166) |  |
| *Moderate/High* | 28% (46/167) | 27% (44/166) | 34% (56/167) | 38% (63/166) |  |
| **Frequency of drug use** |  |  |  |  | 0.246 |
| *Never/Rare* | 83% (139/167) | 86% (142/166) | 80% (132/166) | 78% (129/166) |  |
| *Monthly/Weekly/Daily* | 17% (28/167) | 14% (24/166) | 20% (34/166) | 22% (37/166) |  |
| **Since 3 months ago, feel...** |  |  |  |  | **<0.001** |
| *Less lonely* | 43% (72/166) | 46% (77/167) | 56% (93/167) | 63% (106/167) |  |
| *Same* | 20% (34/166) | 22% (36/167) | 23% (39/167) | 20% (33/167) |  |
| *Lonelier* | 36% (60/166) | 32% (54/167) | 21% (35/167) | 17% (28/167) |  |
| **Had a social support network** |  |  |  |  | 0.141 |
| *Yes* | 73% (121/166) | 77% (129/167) | 81% (134/165) | 82% (136/165) |  |
| *No/Uncertain* | 27% (45/166) | 23% (38/167) | 19% (31/165) | 18% (29/165) |  |
| **Moderate-severe psychological distress** |  |  |  |  | **0.007** |
| *No* | 34% (57/166) | 36% (60/167) | 51% (84/166) | 45% (74/165) |  |
| *Yes* | 66% (109/166) | 64% (107/167) | 49% (82/166) | 55% (91/165) |  |
| **Currently using hormones** |  |  |  |  | 0.318 |
| *No* | 65% (108/167) | 61% (101/166) | 56% (94/167) | 56% (93/166) |  |
| *Yes* | 35% (59/167) | 39% (65/166) | 44% (73/167) | 44% (73/166) |  |
| **Sought care for COVID-19 symptoms** |  |  |  |  | 0.080 |
| *No symptoms* | 83% (138/167) | 90% (150/167) | 90% (151/167) | 80% (125/157) |  |
| *Sought healthcare* | 7% (12/167) | 6% (10/167) | 4% (6/167) | 10% (15/157) |  |
| *Self-isolated* | 6% (10/167) | 3% (5/167) | 2% (3/167) | 6% (10/157) |  |
| *No healthcare/isolation* | 4% (7/167) | 1% (2/167) | 4% (7/167) | 4% (7/157) |  |

Green coloring used to highlight factors with p≤0.05

# Supplemental Material 13. Characteristics of Food Insecurity Trajectories, by Time

|  | **T1 (N=167)** | | | | | **T2 (N=167)** | | | | | **T3 (N=167)** | | | | | **T4 (N=167)** | | | | |
| --- | --- | --- | --- | --- | --- | --- | --- | --- | --- | --- | --- | --- | --- | --- | --- | --- | --- | --- | --- | --- |
| **Characteristic** | **Never T.**  **N = 115** | **Rare T.**  **N = 21** | **Frequent T.**  **N = 23** | **Persistent T.**  **N = 8** | **p-value** | **Never T.**  **N = 115** | **Rare T.**  **N = 21** | **Frequent T.**  **N = 23** | **Persistent T.**  **N = 8** | **p-value** | **Never T.**  **N = 115** | **Rare T.**  **N = 21** | **Frequent T.**  **N = 23** | **Persistent T.**  **N = 8** | **p-value** | **Never T.**  **N = 115** | **Rare T.**  **N = 21** | **Frequent T.**  **N = 23** | **Persistent T.**  **N = 8** | **p-value** |
| **Age (Continuous)** | 20 (19-20) | 19 (18-21) | 20 (19-21) | 20 (19-20) | 0.901 |  |  |  |  |  |  |  |  |  |  |  |  |  |  |  |
| **Racial identity** |  |  |  |  | 0.214 |  |  |  |  |  |  |  |  |  |  |  |  |  |  |  |
| *White* | 65% (72) | 76% (16) | 48% (11) | 86% (6) |  |  |  |  |  |  |  |  |  |  |  |  |  |  |  |  |
| *Asian/Asian American* | 24% (27) | 19% (4) | 26% (6) | 0% (0) |  |  |  |  |  |  |  |  |  |  |  |  |  |  |  |  |
| *Other/Multiracial* | 11% (12) | 5% (1) | 26% (6) | 14% (1) |  |  |  |  |  |  |  |  |  |  |  |  |  |  |  |  |
| **Hispanic ethnicity** |  |  |  |  | 0.227 |  |  |  |  |  |  |  |  |  |  |  |  |  |  |  |
| *No* | 88% (98) | 90% (19) | 91% (21) | 63% (5) |  |  |  |  |  |  |  |  |  |  |  |  |  |  |  |  |
| *Yes* | 13% (14) | 10% (2) | 9% (2) | 38% (3) |  |  |  |  |  |  |  |  |  |  |  |  |  |  |  |  |
| **School year** |  |  |  |  | 0.531 |  |  |  |  |  |  |  |  |  |  |  |  |  |  |  |
| *First-year* | 30% (35) | 38% (8) | 30% (7) | 13% (1) |  |  |  |  |  |  |  |  |  |  |  |  |  |  |  |  |
| *Sophomore* | 23% (27) | 19% (4) | 22% (5) | 25% (2) |  |  |  |  |  |  |  |  |  |  |  |  |  |  |  |  |
| *Junior* | 34% (39) | 14% (3) | 35% (8) | 38% (3) |  |  |  |  |  |  |  |  |  |  |  |  |  |  |  |  |
| *Senior* | 12% (14) | 29% (6) | 13% (3) | 25% (2) |  |  |  |  |  |  |  |  |  |  |  |  |  |  |  |  |
| **Gender Identity** |  |  |  |  | **0.003** |  |  |  |  | **0.009** |  |  |  |  | **0.015** |  |  |  |  | **0.006** |
| *Cisgender woman* | 98% (113) | 100% (21) | 96% (22) | 63% (5) |  | 97% (111) | 100% (21) | 96% (22) | 63% (5) |  | 95% (109) | 100% (21) | 91% (21) | 63% (5) |  | 96% (110) | 100% (21) | 87% (20) | 63% (5) |  |
| *Transgender/Gender non-conforming* | 2% (2) | 0% (0) | 4% (1) | 38% (3) |  | 3% (4) | 0% (0) | 4% (1) | 38% (3) |  | 5% (6) | 0% (0) | 9% (2) | 38% (3) |  | 4% (5) | 0% (0) | 13% (3) | 38% (3) |  |
| **Received need-based financial aid** |  |  |  |  | **<0.001** |  |  |  |  | **<0.001** |  |  |  |  | **<0.001** |  |  |  |  | **<0.001** |
| *No* | 70% (78) | 65% (13) | 23% (5) | 0% (0) |  | 68% (75) | 74% (14) | 19% (4) | 0% (0) |  | 66% (74) | 78% (14) | 29% (6) | 0% (0) |  | 64% (69) | 74% (14) | 26% (6) | 0% (0) |  |
| *Yes* | 30% (33) | 35% (7) | 77% (17) | 100% (8) |  | 32% (35) | 26% (5) | 81% (17) | 100% (8) |  | 34% (38) | 22% (4) | 71% (15) | 100% (8) |  | 36% (38) | 26% (5) | 74% (17) | 100% (8) |  |
| **Food insecure** |  |  |  |  | **<0.001** |  |  |  |  | **<0.001** |  |  |  |  | **<0.001** |  |  |  |  | **<0.001** |
| *No* | 100% (115) | 90% (19) | 43% (10) | 0% (0) |  | 100% (115) | 76% (16) | 30% (7) | 0% (0) |  | 100% (115) | 76% (16) | 52% (12) | 0% (0) |  | 100% (115) | 57% (12) | 35% (8) | 0% (0) |  |
| *Yes* | 0% (0) | 10% (2) | 57% (13) | 100% (8) |  | 0% (0) | 24% (5) | 70% (16) | 100% (8) |  | 0% (0) | 24% (5) | 48% (11) | 100% (8) |  | 0% (0) | 43% (9) | 65% (15) | 100% (8) |  |
| **Lives with...** |  |  |  |  | 0.768 |  |  |  |  | 0.924 |  |  |  |  | 0.479 |  |  |  |  | 0.286 |
| *Family* | 64% (74) | 48% (10) | 57% (13) | 63% (5) |  | 24% (28) | 19% (4) | 22% (5) | 13% (1) |  | 57% (65) | 52% (11) | 43% (9) | 38% (3) |  | 7% (8) | 10% (2) | 0% (0) | 25% (2) |  |
| *Friends/Roommate(s)/Significant other* | 30% (35) | 48% (10) | 39% (9) | 38% (3) |  | 71% (82) | 71% (15) | 74% (17) | 88% (7) |  | 38% (43) | 43% (9) | 57% (12) | 50% (4) |  | 77% (85) | 75% (15) | 71% (15) | 63% (5) |  |
| *Alone* | 5% (6) | 5% (1) | 4% (1) | 0% (0) |  | 4% (5) | 10% (2) | 4% (1) | 0% (0) |  | 5% (6) | 5% (1) | 0% (0) | 13% (1) |  | 15% (17) | 15% (3) | 29% (6) | 13% (1) |  |
| **Location** |  |  |  |  | 0.063 |  |  |  |  | 0.223 |  |  |  |  | **0.015** |  |  |  |  | 0.136 |
| *Outside NYC metro area* | 58% (67) | 33% (7) | 35% (8) | 38% (3) |  | 23% (26) | 5% (1) | 22% (5) | 13% (1) |  | 50% (57) | 33% (7) | 17% (4) | 25% (2) |  | 5% (6) | 14% (3) | 0% (0) | 25% (2) |  |
| *Off-campus NYC metro area* | 40% (46) | 62% (13) | 57% (13) | 63% (5) |  | 36% (41) | 67% (14) | 39% (9) | 38% (3) |  | 37% (42) | 62% (13) | 52% (12) | 50% (4) |  | 32% (37) | 29% (6) | 22% (5) | 13% (1) |  |
| *On-campus housing* | 2% (2) | 5% (1) | 9% (2) | 0% (0) |  | 42% (48) | 29% (6) | 39% (9) | 50% (4) |  | 14% (16) | 5% (1) | 30% (7) | 25% (2) |  | 63% (72) | 57% (12) | 78% (18) | 63% (5) |  |
| **Home perceived unsafe** |  |  |  |  | **0.004** |  |  |  |  | 0.100 |  |  |  |  | 0.738 |  |  |  |  | 0.468 |
| *No* | 77% (89) | 90% (19) | 68% (15) | 25% (2) |  | 70% (81) | 90% (19) | 70% (16) | 50% (4) |  | 77% (88) | 86% (18) | 74% (17) | 71% (5) |  | 77% (89) | 71% (15) | 65% (15) | 63% (5) |  |
| *Yes* | 23% (26) | 10% (2) | 32% (7) | 75% (6) |  | 30% (34) | 10% (2) | 30% (7) | 50% (4) |  | 23% (27) | 14% (3) | 26% (6) | 29% (2) |  | 23% (26) | 29% (6) | 35% (8) | 38% (3) |  |
| **Social group involvement** |  |  |  |  | 0.220 |  |  |  |  | 0.665 |  |  |  |  | 0.185 |  |  |  |  | 0.314 |
| *No* | 39% (45) | 52% (11) | 30% (7) | 13% (1) |  | 46% (53) | 48% (10) | 39% (9) | 25% (2) |  | 56% (64) | 65% (13) | 43% (10) | 25% (2) |  | 39% (45) | 52% (11) | 26% (6) | 50% (4) |  |
| *Yes* | 61% (70) | 48% (10) | 70% (16) | 88% (7) |  | 54% (61) | 52% (11) | 61% (14) | 75% (6) |  | 44% (50) | 35% (7) | 57% (13) | 75% (6) |  | 61% (69) | 48% (10) | 74% (17) | 50% (4) |  |
| **Sports involvement** |  |  |  |  | 0.093 |  |  |  |  | 0.893 |  |  |  |  | 0.883 |  |  |  |  | 0.773 |
| *No* | 88% (101) | 100% (21) | 100% (23) | 88% (7) |  | 94% (108) | 100% (21) | 96% (22) | 100% (8) |  | 95% (109) | 100% (21) | 96% (22) | 100% (8) |  | 91% (105) | 95% (20) | 96% (22) | 88% (7) |  |
| *Yes* | 12% (14) | 0% (0) | 0% (0) | 13% (1) |  | 6% (7) | 0% (0) | 4% (1) | 0% (0) |  | 5% (6) | 0% (0) | 4% (1) | 0% (0) |  | 9% (10) | 5% (1) | 4% (1) | 13% (1) |  |
| **Relationship status** |  |  |  |  | 0.659 |  |  |  |  | **0.026** |  |  |  |  | 0.202 |  |  |  |  | 0.066 |
| *Single* | 70% (81) | 57% (12) | 68% (15) | 75% (6) |  | 71% (82) | 62% (13) | 48% (11) | 100% (8) |  | 67% (76) | 67% (14) | 48% (11) | 88% (7) |  | 69% (79) | 71% (15) | 43% (10) | 88% (7) |  |
| *In some form of relationship* | 30% (34) | 43% (9) | 32% (7) | 25% (2) |  | 29% (33) | 38% (8) | 52% (12) | 0% (0) |  | 33% (38) | 33% (7) | 52% (12) | 13% (1) |  | 31% (36) | 29% (6) | 57% (13) | 13% (1) |  |
| **Experienced physical/verbal violence** |  |  |  |  | 0.313 |  |  |  |  | 0.651 |  |  |  |  | 0.290 |  |  |  |  | **0.041** |
| *No* | 53% (58) | 71% (15) | 50% (11) | 38% (3) |  | 72% (81) | 75% (15) | 61% (14) | 63% (5) |  | 65% (71) | 85% (17) | 70% (16) | 57% (4) |  | 89% (88) | 89% (16) | 72% (13) | 57% (4) |  |
| *Yes* | 47% (51) | 29% (6) | 50% (11) | 63% (5) |  | 28% (32) | 25% (5) | 39% (9) | 38% (3) |  | 35% (39) | 15% (3) | 30% (7) | 43% (3) |  | 11% (11) | 11% (2) | 28% (5) | 43% (3) |  |
| **Condom use status** |  |  |  |  | 0.726 |  |  |  |  | 0.106 |  |  |  |  | 0.414 |  |  |  |  | 0.877 |
| *No sexual activity* | 71% (80) | 58% (11) | 71% (15) | 88% (7) |  | 69% (79) | 57% (12) | 48% (10) | 88% (7) |  | 60% (66) | 45% (9) | 45% (9) | 67% (4) |  | 55% (63) | 47% (8) | 48% (10) | 50% (4) |  |
| *No condoms* | 13% (15) | 11% (2) | 10% (2) | 0% (0) |  | 10% (11) | 19% (4) | 33% (7) | 0% (0) |  | 13% (14) | 20% (4) | 30% (6) | 0% (0) |  | 15% (17) | 24% (4) | 24% (5) | 25% (2) |  |
| *Used condoms* | 16% (18) | 32% (6) | 19% (4) | 13% (1) |  | 21% (24) | 24% (5) | 19% (4) | 13% (1) |  | 27% (30) | 35% (7) | 25% (5) | 33% (2) |  | 30% (35) | 29% (5) | 29% (6) | 25% (2) |  |
| **Currently smokes/vapes** |  |  |  |  | **0.002** |  |  |  |  | **0.039** |  |  |  |  | 0.559 |  |  |  |  | 0.082 |
| *No* | 99% (114) | 100% (20) | 87% (20) | 75% (6) |  | 97% (112) | 90% (19) | 87% (20) | 88% (7) |  | 93% (105) | 90% (18) | 87% (20) | 88% (7) |  | 96% (109) | 95% (19) | 83% (19) | 88% (7) |  |
| *Yes* | 1% (1) | 0% (0) | 13% (3) | 25% (2) |  | 3% (3) | 10% (2) | 13% (3) | 13% (1) |  | 7% (8) | 10% (2) | 13% (3) | 13% (1) |  | 4% (5) | 5% (1) | 17% (4) | 13% (1) |  |
| **Frequency of alcohol use** |  |  |  |  | 0.567 |  |  |  |  | 0.292 |  |  |  |  | 0.749 |  |  |  |  | 0.539 |
| *Never/Rare* | 72% (83) | 67% (14) | 83% (19) | 63% (5) |  | 73% (83) | 62% (13) | 87% (20) | 75% (6) |  | 63% (73) | 71% (15) | 74% (17) | 75% (6) |  | 59% (68) | 60% (12) | 74% (17) | 75% (6) |  |
| *Moderate/High* | 28% (32) | 33% (7) | 17% (4) | 38% (3) |  | 27% (31) | 38% (8) | 13% (3) | 25% (2) |  | 37% (42) | 29% (6) | 26% (6) | 25% (2) |  | 41% (47) | 40% (8) | 26% (6) | 25% (2) |  |
| **Frequency of drug use** |  |  |  |  | 0.145 |  |  |  |  | 0.406 |  |  |  |  | 0.650 |  |  |  |  | **0.022** |
| *Never/Rare* | 87% (100) | 81% (17) | 70% (16) | 75% (6) |  | 85% (97) | 95% (20) | 83% (19) | 75% (6) |  | 81% (92) | 81% (17) | 78% (18) | 63% (5) |  | 81% (93) | 90% (18) | 61% (14) | 50% (4) |  |
| *Monthly/Weekly/Daily* | 13% (15) | 19% (4) | 30% (7) | 25% (2) |  | 15% (17) | 5% (1) | 17% (4) | 25% (2) |  | 19% (22) | 19% (4) | 22% (5) | 38% (3) |  | 19% (22) | 10% (2) | 39% (9) | 50% (4) |  |
|  |  |  |  |  |  |  |  |  |  |  |  |  |  |  |  |  |  |  |  |  |
|  |  |  |  |  |  |  |  |  |  |  |  |  |  |  |  |  |  |  |  |  |
|  |  |  |  |  |  |  |  |  |  |  |  |  |  |  |  |  |  |  |  |  |
|  |  |  |  |  |  |  |  |  |  |  |  |  |  |  |  |  |  |  |  |  |
| **Since 3 months ago, feel...** |  |  |  |  | 0.359 |  |  |  |  | 0.766 |  |  |  |  | 0.246 |  |  |  |  | 0.835 |
| *Less lonely* | 46% (52) | 43% (9) | 35% (8) | 38% (3) |  | 48% (55) | 43% (9) | 43% (10) | 38% (3) |  | 57% (66) | 57% (12) | 61% (14) | 13% (1) |  | 63% (73) | 62% (13) | 70% (16) | 50% (4) |  |
| *Same* | 22% (25) | 19% (4) | 9% (2) | 38% (3) |  | 18% (21) | 24% (5) | 30% (7) | 38% (3) |  | 23% (27) | 24% (5) | 17% (4) | 38% (3) |  | 20% (23) | 24% (5) | 17% (4) | 13% (1) |  |
| *Lonelier* | 32% (37) | 38% (8) | 57% (13) | 25% (2) |  | 34% (39) | 33% (7) | 26% (6) | 25% (2) |  | 19% (22) | 19% (4) | 22% (5) | 50% (4) |  | 17% (19) | 14% (3) | 13% (3) | 38% (3) |  |
| **Had a social support network** |  |  |  |  | **0.001** |  |  |  |  | **0.035** |  |  |  |  | **<0.001** |  |  |  |  | 0.692 |
| *Yes* | 82% (93) | 62% (13) | 48% (11) | 50% (4) |  | 81% (93) | 86% (18) | 61% (14) | 50% (4) |  | 88% (100) | 81% (17) | 52% (12) | 63% (5) |  | 83% (95) | 86% (18) | 74% (17) | 86% (6) |  |
| *No/Uncertain* | 18% (21) | 38% (8) | 52% (12) | 50% (4) |  | 19% (22) | 14% (3) | 39% (9) | 50% (4) |  | 12% (13) | 19% (4) | 48% (11) | 38% (3) |  | 17% (19) | 14% (3) | 26% (6) | 14% (1) |  |
| **Moderate-severe psychological distress** |  |  |  |  | **0.003** |  |  |  |  | 0.079 |  |  |  |  | 0.081 |  |  |  |  | 0.168 |
| *No* | 41% (47) | 38% (8) | 9% (2) | 0% (0) |  | 39% (45) | 43% (9) | 13% (3) | 38% (3) |  | 57% (65) | 43% (9) | 30% (7) | 38% (3) |  | 50% (57) | 45% (9) | 26% (6) | 29% (2) |  |
| *Yes* | 59% (68) | 62% (13) | 91% (20) | 100% (8) |  | 61% (70) | 57% (12) | 87% (20) | 63% (5) |  | 43% (49) | 57% (12) | 70% (16) | 63% (5) |  | 50% (58) | 55% (11) | 74% (17) | 71% (5) |  |
| **Currently using hormones** |  |  |  |  | 0.431 |  |  |  |  | 0.533 |  |  |  |  | 0.841 |  |  |  |  | 0.785 |
| *No* | 63% (72) | 57% (12) | 78% (18) | 75% (6) |  | 58% (67) | 57% (12) | 73% (16) | 75% (6) |  | 55% (63) | 57% (12) | 65% (15) | 50% (4) |  | 57% (66) | 45% (9) | 57% (13) | 63% (5) |  |
| *Yes* | 37% (43) | 43% (9) | 22% (5) | 25% (2) |  | 42% (48) | 43% (9) | 27% (6) | 25% (2) |  | 45% (52) | 43% (9) | 35% (8) | 50% (4) |  | 43% (49) | 55% (11) | 43% (10) | 38% (3) |  |
| **Sought care for COVID-19 symptoms** |  |  |  |  | 0.322 |  |  |  |  | 0.052 |  |  |  |  | 0.103 |  |  |  |  | **0.048** |
| *No symptoms* | 83% (96) | 81% (17) | 83% (19) | 75% (6) |  | 92% (106) | 67% (14) | 96% (22) | 100% (8) |  | 92% (106) | 76% (16) | 91% (21) | 100% (8) |  | 85% (92) | 60% (12) | 73% (16) | 71% (5) |  |
| *Sought healthcare* | 8% (9) | 0% (0) | 9% (2) | 13% (1) |  | 3% (4) | 24% (5) | 4% (1) | 0% (0) |  | 4% (5) | 5% (1) | 0% (0) | 0% (0) |  | 6% (7) | 15% (3) | 14% (3) | 29% (2) |  |
| *Self-isolated* | 5% (6) | 14% (3) | 0% (0) | 13% (1) |  | 3% (3) | 10% (2) | 0% (0) | 0% (0) |  | 0% (0) | 10% (2) | 4% (1) | 0% (0) |  | 4% (4) | 15% (3) | 14% (3) | 0% (0) |  |
| *No healthcare/isolation* | 3% (4) | 5% (1) | 9% (2) | 0% (0) |  | 2% (2) | 0% (0) | 0% (0) | 0% (0) |  | 3% (4) | 10% (2) | 4% (1) | 0% (0) |  | 5% (5) | 10% (2) | 0% (0) | 0% (0) |  |

T. = Trajectory

Green coloring used to highlight factors with p≤0.05

# Supplemental Material 14. Characteristics of Food Insecurity Trajectories, across Time

|  | **Never Trajectory (N=115)** | | | | | **Rare Trajectory (N=84)** | | | | | **Frequent Trajectory (N=23)** | | | | | **Persistent Trajectory (N=8)** | | | | |
| --- | --- | --- | --- | --- | --- | --- | --- | --- | --- | --- | --- | --- | --- | --- | --- | --- | --- | --- | --- | --- |
| **Characteristic** | **T1** | **T2** | **T3** | **T4** | **p-value** | **T1** | **T2** | **T3** | **T4** | **p-value** | **T1** | **T2** | **T3** | **T4** | **p-value** | **T1** | **T2** | **T3** | **T4** | **p-value** |
| **Age (Continuous)** | 20 (19-20) |  |  |  |  | 19 (18-21) |  |  |  |  | 20 (19-21) |  |  |  |  | 20 (19-20) |  |  |  |  |
| **Racial identity** |  |  |  |  |  |  |  |  |  |  |  |  |  |  |  |  |  |  |  |  |
| *White* | 65% (72) |  |  |  |  | 76% (16) |  |  |  |  | 48% (11) |  |  |  |  | 86% (6) |  |  |  |  |
| *Asian/Asian American* | 24% (27) |  |  |  |  | 19% (4) |  |  |  |  | 26% (6) |  |  |  |  | 0% (0) |  |  |  |  |
| *Other/Multiracial* | 11% (12) |  |  |  |  | 5% (1) |  |  |  |  | 26% (6) |  |  |  |  | 14% (1) |  |  |  |  |
| **Hispanic ethnicity** |  |  |  |  |  |  |  |  |  |  |  |  |  |  |  |  |  |  |  |  |
| *No* | 88% (98) |  |  |  |  | 90% (19) |  |  |  |  | 91% (21) |  |  |  |  | 63% (5) |  |  |  |  |
| *Yes* | 13% (14) |  |  |  |  | 10% (2) |  |  |  |  | 9% (2) |  |  |  |  | 38% (3) |  |  |  |  |
| **School year** |  |  |  |  |  |  |  |  |  |  |  |  |  |  |  |  |  |  |  |  |
| *First-year* | 30% (35) |  |  |  |  | 38% (8) |  |  |  |  | 30% (7) |  |  |  |  | 13% (1) |  |  |  |  |
| *Sophomore* | 23% (27) |  |  |  |  | 19% (4) |  |  |  |  | 22% (5) |  |  |  |  | 25% (2) |  |  |  |  |
| *Junior* | 34% (39) |  |  |  |  | 14% (3) |  |  |  |  | 35% (8) |  |  |  |  | 38% (3) |  |  |  |  |
| *Senior* | 12% (14) |  |  |  |  | 29% (6) |  |  |  |  | 13% (3) |  |  |  |  | 25% (2) |  |  |  |  |
| **Gender Identity** |  |  |  |  | 0.612 |  |  |  |  | >0.999 |  |  |  |  | 0.830 |  |  |  |  | >0.999 |
| *Cisgender woman* | 98% (113) | 97% (111) | 95% (109) | 96% (110) |  | 100% (21) | 100% (21) | 100% (21) | 100% (21) |  | 96% (22) | 96% (22) | 91% (21) | 87% (20) |  | 63% (5) | 63% (5) | 63% (5) | 63% (5) |  |
| *Transgender/Gender non-conforming* | 2% (2) | 3% (4) | 5% (6) | 4% (5) |  | 0% (0) | 0% (0) | 0% (0) | 0% (0) |  | 4% (1) | 4% (1) | 9% (2) | 13% (3) |  | 38% (3) | 38% (3) | 38% (3) | 38% (3) |  |
| **Received need-based financial aid** |  |  |  |  | 0.815 |  |  |  |  | 0.858 |  |  |  |  | 0.898 |  |  |  |  | >0.999 |
| *No* | 70% (78) | 68% (75) | 66% (74) | 64% (69) |  | 65% (13) | 74% (14) | 78% (14) | 74% (14) |  | 23% (5) | 19% (4) | 29% (6) | 26% (6) |  | 0% (0) | 0% (0) | 0% (0) | 0% (0) |  |
| *Yes* | 30% (33) | 32% (35) | 34% (38) | 36% (38) |  | 35% (7) | 26% (5) | 22% (4) | 26% (5) |  | 77% (17) | 81% (17) | 71% (15) | 74% (17) |  | 100% (8) | 100% (8) | 100% (8) | 100% (8) |  |
| **Food insecure** |  |  |  |  | >0.999 |  |  |  |  | 0.099 |  |  |  |  | 0.446 |  |  |  |  | >0.999 |
| *No* | 100% (115) | 100% (115) | 100% (115) | 100% (115) |  | 90% (19) | 76% (16) | 76% (16) | 57% (12) |  | 43% (10) | 30% (7) | 52% (12) | 35% (8) |  | 0% (0) | 0% (0) | 0% (0) | 0% (0) |  |
| *Yes* | 0% (0) | 0% (0) | 0% (0) | 0% (0) |  | 10% (2) | 24% (5) | 24% (5) | 43% (9) |  | 57% (13) | 70% (16) | 48% (11) | 65% (15) |  | 100% (8) | 100% (8) | 100% (8) | 100% (8) |  |
| **Lives with...** |  |  |  |  | **<0.001** |  |  |  |  | **0.027** |  |  |  |  | **<0.001** |  |  |  |  | 0.268 |
| *Family* | 64% (74) | 24% (28) | 57% (65) | 7% (8) |  | 48% (10) | 19% (4) | 52% (11) | 10% (2) |  | 57% (13) | 22% (5) | 43% (9) | 0% (0) |  | 63% (5) | 13% (1) | 38% (3) | 25% (2) |  |
| *Friends/Roommate(s)/Significant other* | 30% (35) | 71% (82) | 38% (43) | 77% (85) |  | 48% (10) | 71% (15) | 43% (9) | 75% (15) |  | 39% (9) | 74% (17) | 57% (12) | 71% (15) |  | 38% (3) | 88% (7) | 50% (4) | 63% (5) |  |
| *Alone* | 5% (6) | 4% (5) | 5% (6) | 15% (17) |  | 5% (1) | 10% (2) | 5% (1) | 15% (3) |  | 4% (1) | 4% (1) | 0% (0) | 29% (6) |  | 0% (0) | 0% (0) | 13% (1) | 13% (1) |  |
| **Location** |  |  |  |  | **<0.001** |  |  |  |  | **<0.001** |  |  |  |  | **<0.001** |  |  |  |  | 0.136 |
| *Outside NYC metro area* | 58% (67) | 23% (26) | 50% (57) | 5% (6) |  | 33% (7) | 5% (1) | 33% (7) | 14% (3) |  | 35% (8) | 22% (5) | 17% (4) | 0% (0) |  | 38% (3) | 13% (1) | 25% (2) | 25% (2) |  |
| *Off-campus NYC metro area* | 40% (46) | 36% (41) | 37% (42) | 32% (37) |  | 62% (13) | 67% (14) | 62% (13) | 29% (6) |  | 57% (13) | 39% (9) | 52% (12) | 22% (5) |  | 63% (5) | 38% (3) | 50% (4) | 13% (1) |  |
| *On-campus housing* | 2% (2) | 42% (48) | 14% (16) | 63% (72) |  | 5% (1) | 29% (6) | 5% (1) | 57% (12) |  | 9% (2) | 39% (9) | 30% (7) | 78% (18) |  | 0% (0) | 50% (4) | 25% (2) | 63% (5) |  |
| **Home perceived unsafe** |  |  |  |  | 0.552 |  |  |  |  | 0.311 |  |  |  |  | 0.935 |  |  |  |  | 0.342 |
| *No* | 77% (89) | 70% (81) | 77% (88) | 77% (89) |  | 90% (19) | 90% (19) | 86% (18) | 71% (15) |  | 68% (15) | 70% (16) | 74% (17) | 65% (15) |  | 25% (2) | 50% (4) | 71% (5) | 63% (5) |  |
| *Yes* | 23% (26) | 30% (34) | 23% (27) | 23% (26) |  | 10% (2) | 10% (2) | 14% (3) | 29% (6) |  | 32% (7) | 30% (7) | 26% (6) | 35% (8) |  | 75% (6) | 50% (4) | 29% (2) | 38% (3) |  |
| **Social group involvement** |  |  |  |  | **0.032** |  |  |  |  | 0.714 |  |  |  |  | 0.590 |  |  |  |  | 0.524 |
| *No* | 39% (45) | 46% (53) | 56% (64) | 39% (45) |  | 52% (11) | 48% (10) | 65% (13) | 52% (11) |  | 30% (7) | 39% (9) | 43% (10) | 26% (6) |  | 13% (1) | 25% (2) | 25% (2) | 50% (4) |  |
| *Yes* | 61% (70) | 54% (61) | 44% (50) | 61% (69) |  | 48% (10) | 52% (11) | 35% (7) | 48% (10) |  | 70% (16) | 61% (14) | 57% (13) | 74% (17) |  | 88% (7) | 75% (6) | 75% (6) | 50% (4) |  |
| **Sports involvement** |  |  |  |  | 0.207 |  |  |  |  | >0.999 |  |  |  |  | >0.999 |  |  |  |  | >0.999 |
| *No* | 88% (101) | 94% (108) | 95% (109) | 91% (105) |  | 100% (21) | 100% (21) | 100% (21) | 95% (20) |  | 100% (23) | 96% (22) | 96% (22) | 96% (22) |  | 88% (7) | 100% (8) | 100% (8) | 88% (7) |  |
| *Yes* | 12% (14) | 6% (7) | 5% (6) | 9% (10) |  | 0% (0) | 0% (0) | 0% (0) | 5% (1) |  | 0% (0) | 4% (1) | 4% (1) | 4% (1) |  | 13% (1) | 0% (0) | 0% (0) | 13% (1) |  |
| **Relationship status** |  |  |  |  | 0.878 |  |  |  |  | 0.792 |  |  |  |  | 0.349 |  |  |  |  | 0.886 |
| *Single* | 70% (81) | 71% (82) | 67% (76) | 69% (79) |  | 57% (12) | 62% (13) | 67% (14) | 71% (15) |  | 68% (15) | 48% (11) | 48% (11) | 43% (10) |  | 75% (6) | 100% (8) | 88% (7) | 88% (7) |  |
| *In some form of relationship* | 30% (34) | 29% (33) | 33% (38) | 31% (36) |  | 43% (9) | 38% (8) | 33% (7) | 29% (6) |  | 32% (7) | 52% (12) | 52% (12) | 57% (13) |  | 25% (2) | 0% (0) | 13% (1) | 13% (1) |  |
| **Experienced physical/verbal violence** |  |  |  |  | **<0.001** |  |  |  |  | 0.518 |  |  |  |  | 0.438 |  |  |  |  | 0.827 |
| *No* | 53% (58) | 72% (81) | 65% (71) | 89% (88) |  | 71% (15) | 75% (15) | 85% (17) | 89% (16) |  | 50% (11) | 61% (14) | 70% (16) | 72% (13) |  | 38% (3) | 63% (5) | 57% (4) | 57% (4) |  |
| *Yes* | 47% (51) | 28% (32) | 35% (39) | 11% (11) |  | 29% (6) | 25% (5) | 15% (3) | 11% (2) |  | 50% (11) | 39% (9) | 30% (7) | 28% (5) |  | 63% (5) | 38% (3) | 43% (3) | 43% (3) |  |
| **Condom use status** |  |  |  |  | 0.109 |  |  |  |  | 0.935 |  |  |  |  | 0.495 |  |  |  |  | 0.436 |
| *No sexual activity* | 71% (80) | 69% (79) | 60% (66) | 55% (63) |  | 58% (11) | 57% (12) | 45% (9) | 47% (8) |  | 71% (15) | 48% (10) | 45% (9) | 48% (10) |  | 88% (7) | 88% (7) | 67% (4) | 50% (4) |  |
| *No condoms* | 13% (15) | 10% (11) | 13% (14) | 15% (17) |  | 11% (2) | 19% (4) | 20% (4) | 24% (4) |  | 10% (2) | 33% (7) | 30% (6) | 24% (5) |  | 0% (0) | 0% (0) | 0% (0) | 25% (2) |  |
| *Used condoms* | 16% (18) | 21% (24) | 27% (30) | 30% (35) |  | 32% (6) | 24% (5) | 35% (7) | 29% (5) |  | 19% (4) | 19% (4) | 25% (5) | 29% (6) |  | 13% (1) | 13% (1) | 33% (2) | 25% (2) |  |
| **Currently smokes/vapes** |  |  |  |  | 0.071 |  |  |  |  | 0.748 |  |  |  |  | >0.999 |  |  |  |  | >0.999 |
| *No* | 99% (114) | 97% (112) | 93% (105) | 96% (109) |  | 100% (20) | 90% (19) | 90% (18) | 95% (19) |  | 87% (20) | 87% (20) | 87% (20) | 83% (19) |  | 75% (6) | 88% (7) | 88% (7) | 88% (7) |  |
| *Yes* | 1% (1) | 3% (3) | 7% (8) | 4% (5) |  | 0% (0) | 10% (2) | 10% (2) | 5% (1) |  | 13% (3) | 13% (3) | 13% (3) | 17% (4) |  | 25% (2) | 13% (1) | 13% (1) | 13% (1) |  |
| **Frequency of alcohol use** |  |  |  |  | 0.072 |  |  |  |  | 0.869 |  |  |  |  | 0.665 |  |  |  |  | >0.999 |
| *Never/Rare* | 72% (83) | 73% (83) | 63% (73) | 59% (68) |  | 67% (14) | 62% (13) | 71% (15) | 60% (12) |  | 83% (19) | 87% (20) | 74% (17) | 74% (17) |  | 63% (5) | 75% (6) | 75% (6) | 75% (6) |  |
| *Moderate/High* | 28% (32) | 27% (31) | 37% (42) | 41% (47) |  | 33% (7) | 38% (8) | 29% (6) | 40% (8) |  | 17% (4) | 13% (3) | 26% (6) | 26% (6) |  | 38% (3) | 25% (2) | 25% (2) | 25% (2) |  |
| **Frequency of drug use** |  |  |  |  | 0.490 |  |  |  |  | 0.455 |  |  |  |  | 0.356 |  |  |  |  | 0.848 |
| *Never/Rare* | 87% (100) | 85% (97) | 81% (92) | 81% (93) |  | 81% (17) | 95% (20) | 81% (17) | 90% (18) |  | 70% (16) | 83% (19) | 78% (18) | 61% (14) |  | 75% (6) | 75% (6) | 63% (5) | 50% (4) |  |
| *Monthly/Weekly/Daily* | 13% (15) | 15% (17) | 19% (22) | 19% (22) |  | 19% (4) | 5% (1) | 19% (4) | 10% (2) |  | 30% (7) | 17% (4) | 22% (5) | 39% (9) |  | 25% (2) | 25% (2) | 38% (3) | 50% (4) |  |
| **Since 3 months ago, feel...** |  |  |  |  | **0.013** |  |  |  |  | 0.629 |  |  |  |  | **0.033** |  |  |  |  | 0.706 |
| *Less lonely* | 46% (52) | 48% (55) | 57% (66) | 63% (73) |  | 43% (9) | 43% (9) | 57% (12) | 62% (13) |  | 35% (8) | 43% (10) | 61% (14) | 70% (16) |  | 38% (3) | 38% (3) | 13% (1) | 50% (4) |  |
| *Same* | 22% (25) | 18% (21) | 23% (27) | 20% (23) |  | 19% (4) | 24% (5) | 24% (5) | 24% (5) |  | 9% (2) | 30% (7) | 17% (4) | 17% (4) |  | 38% (3) | 38% (3) | 38% (3) | 13% (1) |  |
| *Lonelier* | 32% (37) | 34% (39) | 19% (22) | 17% (19) |  | 38% (8) | 33% (7) | 19% (4) | 14% (3) |  | 57% (13) | 26% (6) | 22% (5) | 13% (3) |  | 25% (2) | 25% (2) | 50% (4) | 38% (3) |  |
| **Had a social support network** |  |  |  |  | 0.401 |  |  |  |  | 0.252 |  |  |  |  | 0.288 |  |  |  |  | 0.569 |
| *Yes* | 82% (93) | 81% (93) | 88% (100) | 83% (95) |  | 62% (13) | 86% (18) | 81% (17) | 86% (18) |  | 48% (11) | 61% (14) | 52% (12) | 74% (17) |  | 50% (4) | 50% (4) | 63% (5) | 86% (6) |  |
| *No/Uncertain* | 18% (21) | 19% (22) | 12% (13) | 17% (19) |  | 38% (8) | 14% (3) | 19% (4) | 14% (3) |  | 52% (12) | 39% (9) | 48% (11) | 26% (6) |  | 50% (4) | 50% (4) | 38% (3) | 14% (1) |  |
| **Moderate-severe psychological distress** |  |  |  |  | **0.024** |  |  |  |  | 0.975 |  |  |  |  | 0.239 |  |  |  |  | 0.274 |
| *No* | 41% (47) | 39% (45) | 57% (65) | 50% (57) |  | 38% (8) | 43% (9) | 43% (9) | 45% (9) |  | 9% (2) | 13% (3) | 30% (7) | 26% (6) |  | 0% (0) | 38% (3) | 38% (3) | 29% (2) |  |
| *Yes* | 59% (68) | 61% (70) | 43% (49) | 50% (58) |  | 62% (13) | 57% (12) | 57% (12) | 55% (11) |  | 91% (20) | 87% (20) | 70% (16) | 74% (17) |  | 100% (8) | 63% (5) | 63% (5) | 71% (5) |  |
| **Currently using hormones** |  |  |  |  | 0.682 |  |  |  |  | 0.825 |  |  |  |  | 0.420 |  |  |  |  | 0.848 |
| *No* | 63% (72) | 58% (67) | 55% (63) | 57% (66) |  | 57% (12) | 57% (12) | 57% (12) | 45% (9) |  | 78% (18) | 73% (16) | 65% (15) | 57% (13) |  | 75% (6) | 75% (6) | 50% (4) | 63% (5) |  |
| *Yes* | 37% (43) | 42% (48) | 45% (52) | 43% (49) |  | 43% (9) | 43% (9) | 43% (9) | 55% (11) |  | 22% (5) | 27% (6) | 35% (8) | 43% (10) |  | 25% (2) | 25% (2) | 50% (4) | 38% (3) |  |
| **Sought care for COVID-19 symptoms** |  |  |  |  | 0.238 |  |  |  |  | 0.320 |  |  |  |  | 0.084 |  |  |  |  | 0.149 |
| *No symptoms* | 83% (96) | 92% (106) | 92% (106) | 85% (92) |  | 81% (17) | 67% (14) | 76% (16) | 60% (12) |  | 83% (19) | 96% (22) | 91% (21) | 73% (16) |  | 75% (6) | 100% (8) | 100% (8) | 71% (5) |  |
| *Sought healthcare* | 8% (9) | 3% (4) | 4% (5) | 6% (7) |  | 0% (0) | 24% (5) | 5% (1) | 15% (3) |  | 9% (2) | 4% (1) | 0% (0) | 14% (3) |  | 13% (1) | 0% (0) | 0% (0) | 29% (2) |  |
| *Self-isolated* | 5% (6) | 3% (3) | 0% (0) | 4% (4) |  | 14% (3) | 10% (2) | 10% (2) | 15% (3) |  | 0% (0) | 0% (0) | 4% (1) | 14% (3) |  | 13% (1) | 0% (0) | 0% (0) | 0% (0) |  |
| *No healthcare/isolation* | 3% (4) | 2% (2) | 3% (4) | 5% (5) |  | 5% (1) | 0% (0) | 10% (2) | 10% (2) |  | 9% (2) | 0% (0) | 4% (1) | 0% (0) |  | 0% (0) | 0% (0) | 0% (0) | 0% (0) |  |

Green coloring used to highlight factors with p≤0.05

# Supplemental Material 15. Lost-to-follow-up (LTFU) analyses

|  | **GEE results** | | | | **LTFU-GEE** | | **LTFU-Traj** | |
| --- | --- | --- | --- | --- | --- | --- | --- | --- |
|  | **N=167** | | | | **N=556** | | **N=556** | |
| **Characteristic** | **FI vs. Not** | | | | **Lost vs. Included** | | **Excluded vs. Included in Trajectory Sample** | |
|  | **Unadjusted** | | **Adjusted** | | **Unadjusted** | | **Unadjusted** | |
|  | **RR [95% CI]** | **p-value** | **ARR [95% CI]** | **p-value** | **RR [95% CI]** | **p-value** | **PR [95% CI]** | **p-value** |
| **Associated with FI and Missingness (LTFU)** | | | | | | | | |
| **Lives with...** |  |  |  |  |  |  |  |  |
| *Alone* | 1.00 [ref] |  |  |  | 1.00 [ref] |  | 1.00 [ref] |  |
| *Friends / Roommate(s) / Significant other* | 0.63 [0.40-0.99] | **0.045** |  |  | 1.04 [0.82-1.31] | 0.739 | 1.13 [1.01-1.26] | **0.038** |
| *Family* | 0.43 [0.24-0.77] | **0.004** |  |  | 0.93 [0.73-1.17] | 0.525 | 1.11 [0.88-1.40] | 0.370 |
| **Associated with Missingness (LTFU)** | | | | | | | | |
| **Age (Continuous)** | 1.05 [0.86-1.29] | 0.606 |  |  | 1.05 [1.01-1.09] | **0.024** | 1.05 [1.01-1.09] | **0.027** |
| **Racial identity** |  |  |  |  |  |  |  |  |
| *White* | 1.00 [ref] |  |  |  | 1.00 [ref] |  | 1.00 [ref] |  |
| *Asian/Asian American* | 0.80 [0.40-1.59] | 0.519 |  |  | 0.96 [0.82-1.12] | 0.574 | 0.96 [0.82-1.12] | 0.581 |
| *Other/Multiracial* | 1.59 [0.79-3.18] | 0.195 |  |  | 1.13 [0.99-1.29] | 0.062 | 1.14 [1.00-1.30] | **0.048** |
| **School year** |  |  |  |  |  |  |  |  |
| *First-year* | 1.00 [ref] |  |  |  | 1.00 [ref] |  | 1.00 [ref] |  |
| *Sophomore* | 1.13 [0.52-2.45] | 0.761 |  |  | 1.08 [0.91-1.28] | 0.390 | 1.06 [0.90-1.26] | 0.485 |
| *Junior* | 1.16 [0.57-2.37] | 0.674 |  |  | 1.00 [0.85-1.18] | 0.996 | 1.00 [0.85-1.19] | 0.956 |
| *Senior* | 1.55 [0.73-3.30] | 0.250 |  |  | 1.27 [1.10-1.47] | **0.001** | 1.26 [1.09-1.45] | **0.002** |
| **Condom use status** |  |  |  |  |  |  |  |  |
| *No sexual activity* | 1.00 [ref] |  |  |  | 1.00 [ref] |  | 1.00 [ref] |  |
| *No condoms* | 1.53 [0.92-2.56] | 0.100 |  |  | 1.14 [0.99-1.31] | 0.069 | 1.15 [1.00-1.32] | **0.049** |
| *Used condoms* | 0.99 [0.55-1.79] | 0.984 |  |  | 1.07 [0.93-1.23] | 0.347 | 1.07 [0.93-1.23] | 0.348 |
| **Currently smokes/vapes** |  |  |  |  |  |  |  |  |
| *No* | 1.00 [ref] |  |  |  | 1.00 [ref] |  | 1.00 [ref] |  |
| *Yes* | 1.09 [0.46-2.57] | 0.843 |  |  | 1.24 [1.08-1.42] | **0.002** | 1.25 [1.09-1.43] | **0.002** |
| **Frequency of drug use** |  |  |  |  |  |  |  |  |
| *Never/Rare* | 1.00 [ref] |  |  |  | 1.00 [ref] |  | 1.00 [ref] |  |
| *Monthly/Weekly/Daily* | 1.05 [0.70-1.58] | 0.824 |  |  | 1.13 [1.00-1.27] | **0.049** | 1.12 [1.00-1.26] | 0.058 |
| **Moderate-severe psychological distress** |  |  |  |  |  |  |  |  |
| *No* | 1.00 [ref] |  |  |  | 1.00 [ref] |  | 1.00 [ref] |  |
| *Yes* | 1.30 [0.89-1.90] | 0.176 |  |  | 1.20 [1.04-1.38] | **0.014** | 1.21 [1.05-1.39] | **0.010** |
| **Sought care for COVID-19 symptoms** |  |  |  |  |  |  |  |  |
| *No symptoms* | 1.00 [ref] |  |  |  | 1.00 [ref] |  | 1.00 [ref] |  |
| *Sought healthcare* | 0.75 [0.36-1.55] | 0.434 |  |  | 1.19 [1.03-1.37] | **0.017** | 1.18 [1.02-1.35] | **0.023** |
| *Self-isolated* | 1.84 [0.86-3.94] | 0.117 |  |  | 1.09 [0.90-1.33] | 0.356 | 1.10 [0.91-1.33] | 0.333 |
| *No healthcare/isolation* | 0.56 [0.19-1.66] | 0.295 |  |  | 0.94 [0.66-1.33] | 0.714 | 0.93 [0.65-1.31] | 0.662 |
| **Sports involvement** |  |  |  |  |  |  |  |  |
| *No* | 1.00 [ref] |  |  |  | 1.00 [ref] |  | 1.00 [ref] |  |
| *Yes* | 0.98 [0.49-1.96] | 0.963 |  |  | 1.15 [1.01-1.31] | **0.038** | 1.16 [1.02-1.32] | **0.027** |
| **Food insecure** |  |  |  |  |  |  |  |  |
| *No* |  |  |  |  | 1.00 [ref] |  | 1.00 [ref] |  |
| *Yes* |  |  |  |  | 1.21 [1.08-1.35] | **<0.001** | 1.20 [1.07-1.34] | **0.001** |
| **Associated with FI** | | | | | | | | |
| **Gender Identity** |  |  |  |  |  |  |  |  |
| *Cisgender woman* | 1.00 [ref] |  | 1.00 [ref] |  | 1.00 [ref] |  | 1.00 [ref] |  |
| *Transgender/Gender non-conforming* | 2.36 [1.19-4.70] | **0.014** | 2.04 [1.19-3.48] | **0.009** | 0.97 [0.71-1.33] | 0.873 | 0.98 [0.72-1.33] | 0.884 |
|  |  |  |  |  |  |  |  |  |
|  |  |  |  |  |  |  |  |  |
|  |  |  |  |  |  |  |  |  |
|  |  |  |  |  |  |  |  |  |
| **Received need-based financial aid** |  |  |  |  |  |  |  |  |
| *No* | 1.00 [ref] |  | 1.00 [ref] |  | 1.00 [ref] |  | 1.00 [ref] |  |
| *Yes* | 3.99 [2.31-6.87] | **<0.001** | 3.48 [2.02-5.99] | **<0.001** | 1.06 [0.95-1.19] | 0.288 | 1.06 [0.95-1.19] | 0.268 |
| **Home perceived unsafe** |  |  |  |  |  |  |  |  |
| *No* | 1.00 [ref] |  | 1.00 [ref] |  | 1.00 [ref] |  | 1.00 [ref] |  |
| *Yes* | 2.09 [1.50-2.92] | **<0.001** | 1.87 [1.32-2.65] | **<0.001** | 0.98 [0.86-1.11] | 0.725 | 0.98 [0.86-1.12] | 0.765 |
| **Not associated with FI or Missingness (LTFU)** | | | | | | | | |
| **Hispanic ethnicity** |  |  |  |  |  |  |  |  |
| *No* | 1.00 [ref] |  |  |  | 1.00 [ref] |  | 1.00 [ref] |  |
| *Yes* | 1.40 [0.65-3.00] | 0.385 |  |  | 1.06 [0.91-1.23] | 0.474 | 1.05 [0.90-1.21] | 0.561 |
| **Location** |  |  |  |  |  |  |  |  |
| *Outside NYC metro area* | 1.00 [ref] |  |  |  | 1.00 [ref] |  | 1.00 [ref] |  |
| *Off-campus NYC metro area* | 1.13 [0.69-1.86] | 0.629 |  |  | 1.10 [0.99-1.24] | 0.089 | 1.11 [0.99-1.24] | 0.067 |
| *On-campus housing* | 1.37 [0.93-2.02] | 0.112 |  |  | 0.95 [0.64-1.43] | 0.816 | 0.97 [0.65-1.45] | 0.890 |
| **Social group involvement** |  |  |  |  |  |  |  |  |
| *No* | 1.00 [ref] |  |  |  | 1.00 [ref] |  | 1.00 [ref] |  |
| *Yes* | 1.29 [0.85-1.95] | 0.231 |  |  | 1.00 [0.90-1.12] | 0.951 | 0.99 [0.88-1.11] | 0.850 |
| **Relationship status** |  |  |  |  |  |  |  |  |
| *Single* | 1.00 [ref] |  |  |  | 1.00 [ref] |  | 1.00 [ref] |  |
| *In some form of relationship* | 1.42 [0.94-2.14] | 0.092 |  |  | 0.97 [0.86-1.10] | 0.612 | 0.97 [0.86-1.10] | 0.634 |
| **Experienced physical/verbal violence** |  |  |  |  |  |  |  |  |
| *No* | 1.00 [ref] |  |  |  | 1.00 [ref] |  | 1.00 [ref] |  |
| *Yes* | 0.99 [0.74-1.32] | 0.940 |  |  | 1.12 [1.00-1.25] | 0.059 | 1.11 [0.99-1.24] | 0.082 |
| **Frequency of alcohol use** |  |  |  |  |  |  |  |  |
| *Never/Rare* | 1.00 [ref] |  |  |  | 1.00 [ref] |  | 1.00 [ref] |  |
| *Moderate/High* | 0.64 [0.38-1.07] | 0.089 |  |  | 1.07 [0.96-1.20] | 0.218 | 1.08 [0.97-1.21] | 0.177 |
| **Since 3 months ago, feel...** |  |  |  |  |  |  |  |  |
| *Less lonely* | 1.00 [ref] |  |  |  | 1.00 [ref] |  | 1.00 [ref] |  |
| *Same* | 1.20 [0.81-1.76] | 0.366 |  |  | 1.05 [0.90-1.23] | 0.537 | 1.04 [0.89-1.22] | 0.616 |
| *Lonelier* | 1.07 [0.75-1.52] | 0.696 |  |  | 1.11 [0.98-1.26] | 0.094 | 1.10 [0.98-1.25] | 0.115 |
| **Had a social support network** |  |  |  |  |  |  |  |  |
| *Yes* | 1.00 [ref] |  |  |  | 1.00 [ref] |  | 1.00 [ref] |  |
| *Uncertain* | 0.98 [0.62-1.53] | 0.919 |  |  | 0.92 [0.77-1.11] | 0.397 | 0.93 [0.77-1.12] | 0.432 |
| *No* | 1.03 [0.53-1.98] | 0.938 |  |  | 1.00 [0.85-1.17] | >0.999 | 1.02 [0.87-1.19] | 0.802 |
| **Currently using hormones** |  |  |  |  |  |  |  |  |
| *No* | 1.00 [ref] |  |  |  | 1.00 [ref] |  | 1.00 [ref] |  |
| *Yes* | 1.39 [0.89-2.17] | 0.153 |  |  | 0.99 [0.88-1.11] | 0.888 | 0.99 [0.88-1.11] | 0.858 |

FI = Food Insecure, RR = Risk Ratio, ARR = Adjusted Risk Ratio, PR = Prevalence Ratio

# References

1. United States Department of Agriculture. U.S. Adult Food Security Survey Module: three-stage design, with screeners. 2012.

2. Bickel G, Nord M, Price C, Hamilton W, Cook J. Guide to measuring household food security. United States Department of Agriculture; 2000.

3. Brescia SA, Cuite CL. Underestimating college student food insecurity: marginally food secure students may not be food secure. Nutrients. 2022;14(15):3142.

4. Kessler RC, Andrews G, Colpe LJ, Hiripi E, Mroczek DK, Normand S-L, et al. Short screening scales to monitor population prevalences and trends in non-specific psychological distress. Psychological medicine. 2002;32(6):959-76.

5. Kessler RC, Galea S, Gruber MJ, Sampson NA, Ursano RJ, Wessely S. Trends in mental illness and suicidality after Hurricane Katrina. Molecular psychiatry. 2008;13(4):374-84.
